# Genome-Wide Identification and Expression Profiling of the *PDI* Gene Family Reveals Their Probable Involvement in Abiotic Stress Tolerance in Tomato (*Solanum lycopersicum* L.)

**DOI:** 10.3390/genes12010023

**Published:** 2020-12-25

**Authors:** Antt Htet Wai, Muhammad Waseem, A B M Mahbub Morshed Khan, Ujjal Kumar Nath, Do Jin Lee, Sang Tae Kim, Chang Kil Kim, Mi Young Chung

**Affiliations:** 1 Department of Agricultural Education, Sunchon National University, 413 Jungangno, Suncheon, Jeonnam 540-950, Korea; antthtetwai@mu.edu.mm (A.H.W.); djlee@scnu.ac.kr (D.J.L.); 2 Department of Botany, University of Mandalay, Mandalay 05032, Myanmar; 3 College of Horticulture, South China Agricultural University, Guangzhou 510640, China; m.waseem.botanist@gmail.com; 4 Department of Agricultural Botany, Faculty of Agriculture, Patuakhali Science and Technology University, Patuakhali 8602, Bangladesh; morshed@pstu.ac.bd; 5 Department of Genetics and Plant Breeding, Bangladesh Agricultural University, Mymensingh 2202, Bangladesh; ujjalnath@gmail.com; 6 Department of Medical and Biological Sciences, The Catholic University of Korea, Bucheon, Gyeonggi-do 14662, Korea; stkim@catholic.ac.kr; 7 Department of Horticulture, Kyungpook National University, Daegu 41566, Korea; ckkim@knu.ac.kr

**Keywords:** *Solanum lycopersicum*, protein disulfide isomerases, genome-wide analysis, gene expression, abiotic stress, fruit development

## Abstract

Protein disulfide isomerases (PDI) and PDI-like proteins catalyze the formation and isomerization of protein disulfide bonds in the endoplasmic reticulum and prevent the buildup of misfolded proteins under abiotic stress conditions. In the present study, we conducted the first comprehensive genome-wide exploration of the *PDI* gene family in tomato (*Solanum lycopersicum* L.). We identified 19 tomato *PDI* genes that were unevenly distributed on 8 of the 12 tomato chromosomes, with segmental duplications detected for 3 paralogous gene pairs. Expression profiling of the *PDI* genes revealed that most of them were differentially expressed across different organs and developmental stages of the fruit. Furthermore, most of the *PDI* genes were highly induced by heat, salt, and abscisic acid (ABA) treatments, while relatively few of the genes were induced by cold and nutrient and water deficit (NWD) stresses. The predominant expression of *SlPDI1-1*, *SlPDI1-3*, *SlPDI1-4*, *SlPDI2-1*, *SlPDI4-1*, and *SlPDI5-1* in response to abiotic stress and ABA treatment suggested they play regulatory roles in abiotic stress tolerance in tomato in an ABA-dependent manner. Our results provide new insight into the structure and function of *PDI* genes and will be helpful for the selection of candidate genes involved in fruit development and abiotic stress tolerance in tomato.

## 1. Introduction

Protein disulfide isomerases (PDIs) are endoplasmic reticulum (ER)-resident thiodisulfide oxidoreductases whose primary function is to catalyze the formation, reduction, and rearrangement of disulfide bonds in newly synthesized proteins or target proteins. PDIs are thus of particular importance for proper folding and stability of proteins [1,2,3]. When plants are stressed, PDIs interact with misfolded or denatured proteins to prevent the accumulation of unfolded protein aggregates that would otherwise disrupt normal cellular metabolism, thereby contributing to plant tolerance of adverse environmental conditions [4,5]. The *PDI* gene family encodes PDI and PDI-like (PDIL) proteins that contain at least one redox-active thioredoxin domain responsible for the alteration of disulfide bonds, a feature that distinguishes the *PDI* family from other families in the thioredoxoin superfamily, such as glutaredoxins, ferredoxins, and peroxidoxins [6,7,8].

Although they are typically located in the ER, PDIs have also been found in other cellular locations, such as the nucleus, mitochondria, the cytoplasm, and the extracellular environment [9,10,11,12,13]. Many PDI proteins have been well investigated, particularly in mammals, including endoplasmic reticulum resident protein 57 (ERp57), PDIp, PDI-P5, ERp72, PDI-RELATED (PDIR), and PDI with D-domain (PDI-D). These proteins act as redox catalysts and isomerases in addition to having other functions, such as peptide binding, cell adhesion, and chaperone activities [14,15]. The modular structure of human PDI proteins typically consists of four thioredoxin-like domains (known as a, b, b’, and a’), a linker (x), and a C-terminal extension domain (c), arranged in the order abb’xa’c [16]. The a and a’ domains share homology to thioredoxin and each possesses a classic -Cys-Gly-His-Cys- active site within a conserved arrangement of α-helices and β-strands (β-α-β-α-β-α-β-β-α), which is important for isomerase and redox activity [17]. The middle b and b’ domains show similarity to the thioredoxin domain in their secondary structure but not in their primary sequence, and neither contains an active site, although the b’ domain serves as the primary substrate binding site during isomerization reactions [17,18]. The c domain, located in the C terminal region, is enriched in acidic residues characteristic of calcium-binding proteins and usually ends with a KDEL peptide motif critical for ER retention [19,20].

The completion of multiple genome projects in land plants has allowed the identification of each plant’s complement of *PDI* genes: 21 genes in Arabidopsis (*Arabidopsis thaliana*); 12 in rice (*Oryza sativa*), purple false brome (*Brachypodium distachyon*), and maize (*Zea mays*); 10 in grapevine (*Vitis vinifera*); 9 in wheat (*Triticum aestivum*); and 32 in field mustard (*Brassica rapa*) [21,22]. In Arabidopsis, *PDI* genes play roles in developmental processes such as the biogenesis of transitory starch granules in leaves, seed development through the regulation programmed cell death (PCD) during embryogenesis, and the development of the embryo sac [23,24,25]. Analysis of the rice mutant *enclosed shorter panicle 2* (*esp2*) indicated that OsPDIL1-1 may regulate starch biosynthesis by mediating the segregation of proglutelin and prolamin polypeptides within the ER lumen during the early phase of seed development [26,27]. A role for *PDI* genes during endosperm development in wheat and maize has also been reported [28,29]. Soybean (*Glycine max*) *GmPDIL-1*, *GmPDIL-2*, *GmPDIL-3a*, and *GmPDIL-3b* were also reported to play a role in the proper folding and deposition of storage proteins [28,30].

Diverse environmental stresses, including salinity, heat, drought and cold, are detrimental to plant growth and development and lead to considerable yield loss in crops [31]. To mitigate damage to cellular structures, various abiotic stresses induce the expression of *PDI* genes to assist (re)folding misfolded or unfolded proteins to restore their biological functions, as shown in *B. distachyon*, wheat, Chinese cabbage (*B. rapa* spp. *pekinensis*), and Arabidopsis [22,23,32,33]. PDIs also participate in pathogen resistance, as evidenced by the higher expression of 14 *BrPDI* genes in response to *Fusarium oxysporum* f.sp. *conglutinans* infection in Chinese cabbage and the elevated resistance against powdery mildew conferred by the overexpression of PDI-V in susceptible wheat cultivars [22,34]. Likewise, Arabidopsis *PDI1* is up-regulated by diverse abiotic stresses and its overexpression increases the seed germination rate and promotes root growth under different abiotic stress conditions, underscoring the role of *PDI* genes in abiotic stress tolerance [35].

Tomato (*S. lycopersicum* L.) is a model fleshy fruit plant species whose yields are adversely influenced by several environmental stresses. Thus, tomato has been extensively studied to understand the molecular mechanisms governing fruit development and ripening processes, in addition to enhancing fruit yield under different environmental conditions. Although the role of PDI proteins has been documented in several plant species in the context of plant growth and development and abiotic stress tolerance [22,28,33,35], the tomato *PDI* family has yet to be described. Here, we report the first genome-wide characterization, phylogenetic analysis, and expression profiling of tomato *PDI* genes in various organs and under different abiotic stresses to gain a better understanding of the molecular structure and biological functions of *PDI* genes in tomato.

## 2. Methods

### 2.1. Identification and Sequence Analysis of Protein Disulfide Isomerase (PDI) Genes

We identified tomato *PDI* family members using the Sol Genomics Network (SGN) (http://solgenomics.net/) and the National Center for Biotechnological Information (NCBI) websites using the keyword “PDI”. Arabidopsis PDI protein sequences were retrieved from the Arabidopsis Information Resource (TAIR) database (https://www.arabidopsis.org) [36] and used as a query to perform basic local alignment search tool (BLAST) searches with default parameters at the Sol genomics database [37]. The resulting 19 non-redundant PDI sequences were validated for the presence of a thioredoxin domain, using the NCBI conserved domain (NCBI CDD) search [38] and SMART web tool [39]. We gathered information on tomato *PDI* genes, such as locus name, open reading frame (ORF) length, coding sequence (CDS), and chromosomal locations from SGN [37]. The physicochemical parameters of deduced tomato PDI proteins (number of amino acids, molecular weight [MW], and isoelectric point [pI]) were determined using ProtParam (https://web.expasy.org/protparam/) [40] (Table 1). ProtComp Version 9.0 from SoftBerry (http://linux1.softberry. com/berry.phtml) was used to predict the subcellular localization of tomato PDI proteins [41]. We performed a multi-protein alignment of PDI proteins using Clustal Omega [42]. The exon/intron structures of tomato *PDI* genes were analyzed using the Gene Structure Display Server-2.0 (GSDS-2.0) web server (http://gsds.cbi.pku.edu.cn/index.php) by aligning genomic and coding DNA sequences [43]. Sequence homology across the 19 PDI proteins was investigated using the web tool “Immunomedicine Group” (http://imed.med.ucm.es/Tools/sias.html). We analyzed promoter regions of ~1500 bp upstream of the initiation codon [ATG] to predict putative cis-acting elements present in *PDI* promoters using the PlantCare database (http://bioinformatics.psb.ugent.be/webtools/plantcare/html/) [44].

### 2.2. Phylogenetic and Conserved Motif Analysis of the PDI Gene Family in Tomato

The full length tomato PDI protein sequences were aligned with those of Arabidopsis, poplar (*Populus trichocarpa*), Chinese cabbage, maize, and *B. distachyon* L. using Clustal Omega, followed by phylogenetic analysis using the neighbor-joining (NJ) algorithm in MEGA 6.0 [45]. The deduced amino acid sequences of Chinese cabbage, maize, poplar, and *B. distachyon* L. PDIs were obtained from the literature [22,28]. The gene names and accession numbers used in constructing the phylogenetic tree are listed in Supplementary Table S3. To examine the diversity of functional protein motifs in the putative SlPDI proteins, we employed Multiple Em for Motif Elicitation (MEME) software (http://meme-suite.org/) to identify conserved protein motifs with the following parameters: maximum motif number of 10 and a motif length between six and 50 amino acids [46].

### 2.3. Chromosomal Locations, Gene Duplication, and Microsynteny Analysis of the PDI Gene Family

The start and end locations of *PDI* genes, including their sub-genome information, were collected from SGN [36]. *PDI* positions on chromosomes were analyzed with the online tool MapGene2Chromosome2 (http://mg2c.iask.in/mg2c_v2.1/) [47]. Gene duplications among tomato *PDI* genes were identified with the TBtools software [48] according to the criteria stated by Kong et al. (2013). Genes were considered to be segmentally duplicated when their identity and query coverage were >80% [49]. The synonymous (Ks) and non-synonymous (Ka) nucleotide substitution rates of duplicated *SlPDI* gene pairs were determined using the method of Nei and Gojobori (1986) with Mega 6.0 software [50]. The mode of selection was identified by determining the Ka/Ks ratio [51]. Divergence time (T) for each duplicated gene pair was computed with the formula T = Ks/2r Mya (millions of years), where Ks is the synonymous substitution rate per site and r is the constant for dicotyledonous plants of 1.5 × 10^−8^ substitutions per site per year [52]. The microsyntenic relationship of *PDI* genes between tomato, Arabidopsis, and rice was investigated using a reciprocal BLAST search approach against the entire genomes of these species. The results were visualized using TBtools software [48]. The sequences for the three plants species used for microsynteny analysis were retrieved from Phytozome [53].

### 2.4. Preparation of Plant Materials, Treatments, and Sampling

Tomato seeds of the cultivar Ailsa Craig were germinated on horticultural commercial soil for seedlings (Zeolite (4%), Perlite (7%), vermiculite (4%), cocopeat (68%), peatmoss (14.73%), Humectant (0.065%) and fertilizer (0.2%)) in a growth chamber. Seedlings were then allowed to grow in a controlled environment with a relative humidity ranging from 55% to 70%, adjusted temperature of 25 °C during the day and 20 °C at night, under a 16 h light 8 h dark photoperiod, and a light intensity of 300 μmol m^−2^s^−1^. Fresh leaves, stems, and roots were collected from 28-day-old plants for analysis of tissue-specific expression of *SlPDI* genes. The remaining plants were moved to a greenhouse maintained at a temperature of 18 ± 2 °C and 65–80% relative humidity until the reproductive stage in order to collect flower and fruit samples. Flower samples were taken at three different stages: floral bud, anthesis, and flower senescence. Fruit samples were harvested at six developmental stages: (i) young fruits ~14 days after pollination and about 1 cm in diameter, (ii) immature fruits ~34 days after pollination (IM fruits), (iii) mature green fruits ~45 days after pollination (MG fruits), (iv) fruits at the breaker stage when the green color of mature fruits turns to yellow-orange (B fruits), (v) fruits 3 days after the breaker stage (B3 fruits), and (vi) fruits 7 days after the breaker stage (B7 fruits) [54]. 

In parallel, we used 28-day-old plants with uniform growth since sowing to investigate the expression patterns of *SlPDI* genes in response to different stress treatments. We subjected tomato plants to five different treatments: heat, cold, salt (NaCl), nutrient and water deficit (NWD), and abscisic acid (ABA). Leaves were collected at various time points: 0, 1, 3, 6, 12, and 24 h after onset of treatment. Tomato plants were incubated in a growth cabinet at 40 °C (heat treatment) or 4 °C (cold treatment) for 24 h. NaCl stress treatment was imposed by submerging plant roots in a 200 mM NaCl solution for 24 h. For NWD stress, whole plants were gently pulled out from the soil, their root systems carefully cleaned with fresh water and subsequently placed on a dry paper towel for 24 h [54,55,56,57,58]. ABA treatment was applied by spraying plant leaves with 100 μM ABA [54]. Tomato plants grown in soil under normal conditions (25 °C) served as the 0 h controls for all stress conditions. The samples were collected from three biological replicates, frozen in liquid nitrogen, and stored at −80 °C until RNA extraction.

### 2.5. RNA Isolation and Quantitative Real-Time Polymerase Chain Reaction (PCR)

We extracted total RNA from collected samples using the RNeasy mini kit (QIAGEN, Hilden, Germany) following the manufacturer’s instructions. Trace genomic DNA contamination was removed using an RNase-free DNase I kit (Qiagen, Hilden, Germany). RNA concentration and quality was determined with a NanoDrop^®^ 1000 spectrophotometer (Wilmington, DE, USA). We used 1 µg total RNA per sample for first-strand cDNA synthesis using the Superscript^®^ III First-Strand cDNA synthesis kit (Invitrogen, Carlsbad, CA, USA) following the manufacturer’s protocol. For quantitative real-time polymerase chain reaction (RT-qPCR) analysis, gene-specific primers were designed for all *SlPDI* genes using Primer3 software (http://frodo.wi.mit.edu/primer3/input.htm) (Supplementary Table S4) [59]. The melting curve analysis was conducted to validate the specificity of the amplicon for each primer pair [60]. *18S rRNA* (F: AAAAGGTCGACGCGGGCT, R: CGACAGAAGGGACGAGAC) from tomato was used as reference for normalization [61]. Real-time PCR analysis was conducted in optical 96-well plates using a Light cycler^®^ 96SW 1.1 instrument (Roche, Germany). Each reaction consisted of 10 μL iTaq™ from the SYBR^®^ Green PCR kit (Qiagen, Hilden, Germany), 75−80 ng/μL cDNA, 2 μL each forward and reverse primer, and 7 μL double-distilled water, for a total volume of 20 µL. The conditions for qPCR were: initial denaturation at 95 °C for 5 min, followed by 40 cycles at 94 °C for 10 s, annealing at 58 °C for 10 s, and extension at 72 °C for 15 s. Relative expression ratios for each gene were determined by the 2^−ΔΔCt^ method and normalized to leaf sample values for organ-specific expression; for stress treatments, relative expression values were normalized to leaf sample values harvested at 0 h [62]. Significant differences in the relative expression data were analyzed with Sigmaplot 12.1. (SYSTAT and MYSTAT Products, United States, and Canada) using two-tailed Student’s *t*-tests.

## 3. Results

### 3.1. In Silico Identification of PDI Genes in Tomato 

We identified 19 non-redundant tomato genes that encoded proteins with similarity to PDI, which we designated *SlPDI1-1*-*SlPDI11-3*. The ORFs of *SlPDI* genes showed significant variation in length, ranging from 450 bp (*SlPDI6-1*) to 1743 bp (*SlPDI2-3*), with a mean of 1291 bp. Similarly, the lengths of the predicted PDI proteins varied from 149 to 580 amino acid (aa) for *SlPDI6-1* and *SlPDI2-3*, respectively, with a mean of 451 aa. In addition, the predicted molecular weights (MW) vary from 16.9 to 64.6 kDa. The computed isoelectric points of these proteins ranged from 4.49 to 8.74, indicating that PDIs may be acidic or basic, depending on the SlPDI under consideration. Additional information on *SlPDI* family members, such as locus name and predicted subcellular location, are given in Table 1.

### 3.2. Phylogenetic and Domain Analysis

**Figure 1:**
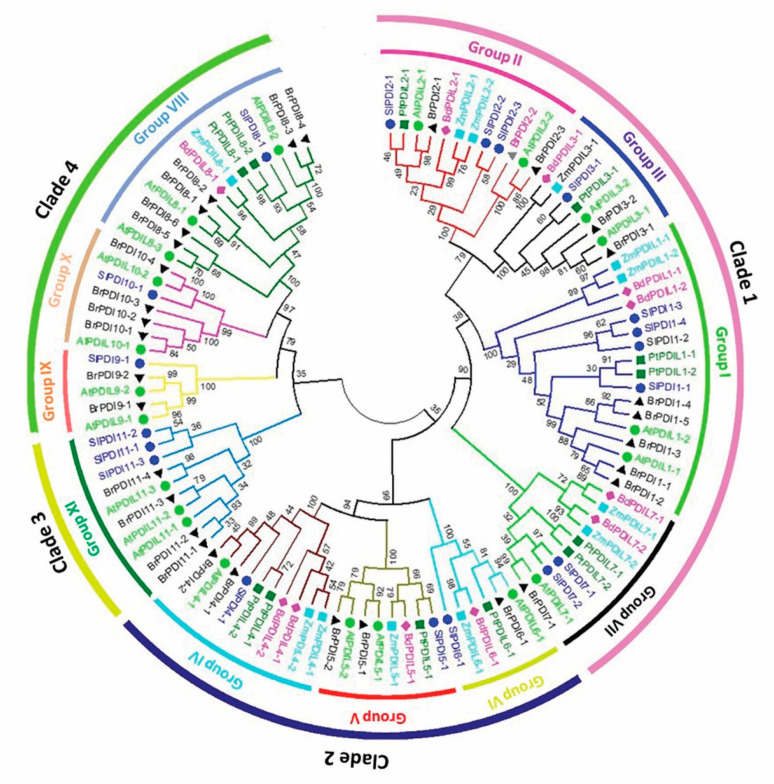
Phylogenetic relationships of protein disulfide isomerase (PDI) proteins identified in tomato (*Solanum lycopersicum*), poplar (*P. trichocarpa*), field mustard (*Brassica rapa*), Arabidopsis (*A. thaliana*), purple false brome (*B. distachyon* L.), and maize (*Z. mays*). The phylogenetic tree was constructed using MEGA6 software by the neighbor-joining method with 1000 bootstrap replicates based on 107 full-length PDI protein sequences from the above-mentioned plant species. The proteins were clustered into 11 groups (I–XI) belonging to four clades. The amino acid sequences used in the phylogenetic analysis are listed in Supplementary Table S3, along with their accession numbers.

**Figure 2:**
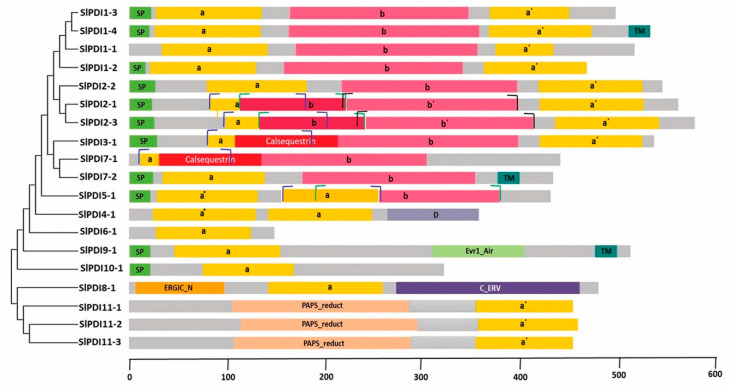
Schematic representation of the domain composition of tomato PDI proteins. The putative signal peptides (SP), the a and b thioredoxin-like domains, the N-terminal calcium binding domain similar to calsequestrin, the D domain (Erp29c), the ERGIC_N domain, the C_ERV (COPII-coated ERV) domain, the Evr1_Air domain, the PAPS_reduct domain, and the transmembrane domain (TM) are displayed. The start and end boundaries of the a, b, and b’ domains, where two of the domain regions overlap, are marked by blue, green, and black hooks, respectively.

We compared the tomato PDI proteins and investigated their evolutionary relationships with respect to other members of the PDI family from various plant species. Accordingly, we generated a phylogenetic tree from the alignment of PDI and PDI-like protein sequences from tomato (with the Sl prefix in the tree, 19 proteins), Arabidopsis (At, 21 proteins)**, maize (Zm, 12 proteins), *B. distachyon* L. (Bd, 11 proteins), poplar (*P. trichocarpa* [Pt], 12 proteins), and *B. rapa* (Br, 32 proteins) (Figure 1). PDI family members clearly clustered into four clades, which were further divided into 11 groups. Tomato PDI members were distributed across all 11 groups, with the largest number of SlPDI proteins belonging to group I. We designated the tomato PDI proteins PDI1-1 to PDI11-3 on the basis of their phylogenetic relationships with other plant PDIs. Of the four clades, clade 1 was the largest clade and comprised groups I, II, III, and VII, of which groups I, II, and III consisted of PDI proteins with two active thioredoxin domains situated at the N-terminus and C-terminal tail. By contrast, group VII included PDI proteins with a single active thioredoxin domain at their N terminus (Figure 2). Based on their structural similarity, PDI proteins belonging to groups I, II, and III may have originated from the duplication of a common ancestral gene, whereas group VII may have arisen by degeneration of one of the two active thioredoxin domains encoded by the ancestral gene or one of its duplicated copies, offering a rational explanation for the close phylogenetic relationship between members of group VII and those of groups I, II, and III. Clade 3 was the smallest and was composed solely of group XI, whose members contained a phosphoadenosine phosphosulfate (PAPS) reductase domain, followed by a single active thioredoxin domain at their C-terminal end. Clade 2 comprised groups IV and V, which contained proteins with two active domains in tandem at their N terminus, as well as group VI, whose constituent proteins contained a single N-terminal active domain. The close phylogenetic relationship between the members of group VI and those of groups IV and V may indicate that group VI genes may have evolved from the degeneration of a gene from group IV or V, resulting in the loss of one active domain, thus paralleling our hypothesis of the evolution of Clade 1 genes. Clade 4 consisted of groups VIII, IX, and X, whose member proteins contained one active thioredoxin domain. Altogether these results indicated that *PDI* genes have diverged greatly over the course of evolution.

**Figure 3:**
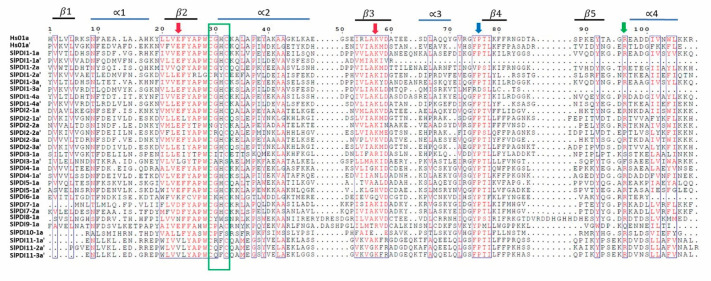
Multiple sequence alignment of a-type domains of tomato PDI proteins alongside those of a classic human PDI. The thioredoxin-like domains in tomato PDIs were annotated by the SMART webtool, and aligned using Clustal Omega. The elements of the secondary structure are indicated by blue (α-helices) and black (β-sheets) bars above the alignment. The red arrows indicate the Glu-Lys charged pair located near the active site, a green arrow indicates the conserved Arg (R) between β5 and α4 of each catalytic domain, and a blue arrow indicates the *cis* Pro (P) near each active site. The –CXXC– catalytic sites are marked by a green box.

All but two of the tomato SlPDI proteins had at least one active thioredoxin domain with the canonical –CXXC– catalytic tetrad for isomerase and redox activities. The exceptions were SlPDI8-1 and SlPDI10-1, which had non-characteristic active sites (CYWS and CPFS, respectively; Table 2). Eleven out of the 19 tomato PDI proteins possessed a predicted N-terminal signal peptide (SP) necessary for polypeptide translocation, whereas only three had a clear transmembrane domain and six had a C-terminal KDEL or RDEL signal for ER retention (Figure 2 and Table 2). A conserved Arg residue, which modulates the redox potential of the active site by regulating the pKa of the Cys residues in the catalytic tetrad, was present in most PDI family members, while some of them possessed the conserved Glu-Lys pair, which is responsible for proton transfer reactions that are critical for the catalytic function of the thioredoxin domain. In addition to the a-type or b-type thioredoxin-like domains, we identified other domains in the tomato PDIs: a calcium-binding domain similar to that of calsequestrin, an Erp29c domain, an ER-Golgi Intermediate Compartment (ERGIC)_N domain, a C_ERV (Endogenous RetroVirus) domain, an Evr1_Air domain, and a PAPS_reduct domain (Figure 2 and Table 2). Alignment of the a-type domains of the predicted tomato PDI proteins and a typical human PDI protein revealed that these domains comprised four β-sheets sandwiched between three α-helices with the –CXXC– catalytic tetrad (Figure 3).

### 3.3. Exon and Intron Distribution and Conserved Motif Analysis

We also investigated the exon-intron structure of *SlPDI* genes (Figure S1) and observed a high degree of structural divergence in the family. For instance, *SlPDI* genes in most of the phylogenetic groups (I, II, III, IV, V, VIII, and IX) contained more introns, ranging from 8 to 14 with a mean of 10, than members in the remaining groups (groups VI, VII, X and XI), which had only 3 or 4 introns. Most *SlPDI* genes falling within the same phylogenetic group displayed almost identical exon-intron organizations, both in terms of intron numbers and exon lengths. For instance, *PDI* members within group I contained 9 or 10 introns, while all group II members (*SlPDI2-1*, *SlPDI2-2*, and *SlPDI2-3*) all had 11 introns. In addition, genes within each gene pair in group VII shared the same exon-intron structural organization. Among all the *SlPDI* members, *SlPDI6-1* and *SlPDI10-1* had the fewest introns, with 3, while *SlPDI8-1* had the most, with 14. 

**Figure 4:**
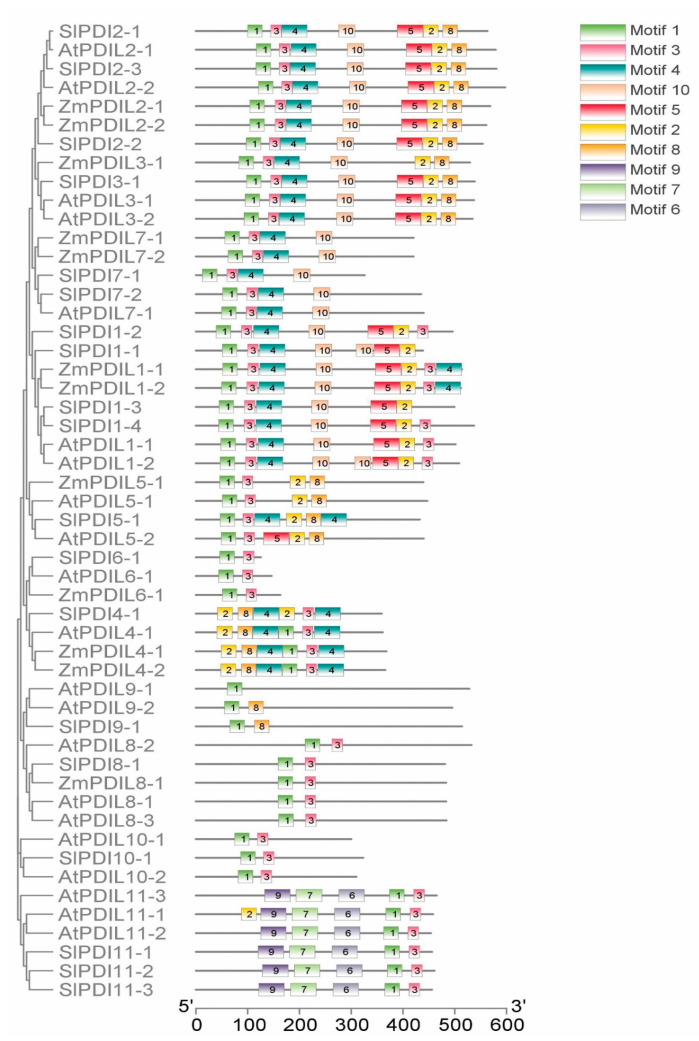
Schematic representation of 10 conserved motifs in PDI proteins from tomato, Arabidopsis, and maize as analyzed by Multiple Em for Motif Elicitation (MEME) software. The a and a’ domains homologous to the thioredoxin (TRX) domains harbor three types of motifs (1, 2, and 3). The –CXXC– catalytic tetrad is included in Motifs 1 and 2, and Motif 3 is closely linked with Motifs 1 and 2. Motif 3 contains the *cis* Pro (P) near the active site that is important for the catalytic activity of thioredoxin. Detailed sequence logos of the motifs are shown in Figure S5.

To characterize the structural diversity of the tomato PDI proteins further, we examined the composition and organization of conserved motifs in SlPDI proteins using the MEME online suite (Figure 4 and Figure S2). Among the 10 conserved motifs we identified, the –CXXC– catalytic triad critical for isomerase and redox activity was detected as Motif 1 and 2. Notably, Motif 1 was common to all phylogenetic groups, except SlPDI4-1. Similarly, Motif 3 was present in all groups, with the exception of group IX. Motif 2 was absent from PDI proteins belonging to groups VI, VII, VIII, IX, and XI. Likewise, Motif 4 was not detected in PDIs from groups V, VI, VIII, IX, X, and XI, with the exception of SlPDI5-1 from group V, which contained two copies of Motif 4. SlPD5-1 was the only protein in group V with this motif, suggesting that Motif 4 does not define group V. Motif 5 was largely restricted to groups I, II, and III, while Motifs 6, 7, and 9, located in the PAPS reductase domain, were unique to group XI. Finally, Motif 8 was specific to groups II, III, IV, and IX, whereas Motif 10 was mainly found in groups I, II, III, and VII from clade 1. PDI proteins from Arabidopsis, maize, and tomato that belonged to the same phylogenetic groups exhibited a similar domain composition and arrangement, suggesting functional conservation across these three plant species. We also noticed different motif distributions among various SlPDI groups, which is likely to drive evolution of the PDI protein family in tomato.

### 3.4. Chromosomal Position, Gene Duplication, and Microsynteny Analysis

**Figure 5:**
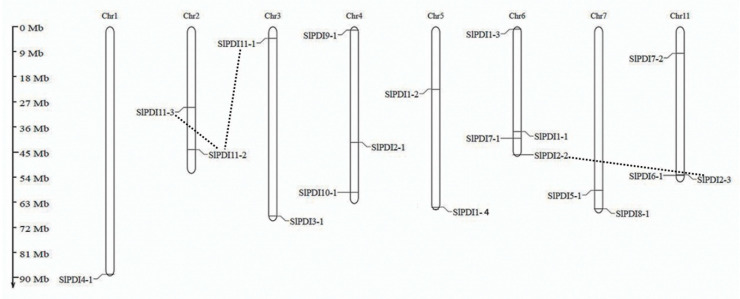
Distribution of the 19 *PDI* genes on the 12 tomato chromosomes. The duplicated genes in the genome are connected by the black dotted lines. Chromosome numbers are indicated at the top of each chromosome. Chromosome sizes and gene locations were estimated using the scale in Megabase pairs (Mbp) to the left of the figure.

We generated a map showing the chromosomal positions of all *SlPDI* genes (Figure 5): eight out of the 12 tomato chromosomes harbored at least one *PDI* gene, with most genes residing close to chromosome ends. Chromosomes 6 and 4 had the most *PDI* genes (4 and 3, respectively). Chromosomes 2, 3, 5, 7, and 11 each possessed 2 *PDI* genes, while chromosome 1 contained a single *PDI* gene. 

Gene duplications such as tandem duplications and segmental duplications play a role in the expansion of a gene family. Genes that show ≥80% sequence identity over at least 80% of their sequence are predicted to be duplicated genes. In addition, if the gene pair is separated by five or fewer genes residing within a 100 kbp window on the same chromosome, they may be considered as tandem-duplicated genes [50]. Based on these criteria, we analyzed the potential for gene duplication events among *PDI* genes and determined that three *SlPDI* pairs, *SlPDI2-2*/*SlPDI2-3*, *SlPDI11-1*/*SlPDI11-2*, and *SlPDI11-2*/*SlPDI11-3*, likely originated by segmental duplication. We did not detect clear evidence for tandem duplications. As expected, all segmentally duplicated gene pairs belonged to the same phylogenetic groups. Only the *SlPDI11-2*/*SlPDI11-3* pair was implicated in a regional duplication event within the same chromosome, while the segmentally duplicated genes involved in the remaining gene pairs (*SlPDI2-2*/*SlPDI2-3* and *SlPDI11-1*/*SlPDI11-2*) mapped to distinct chromosomes (Figure S3). 

**Figure 6:**
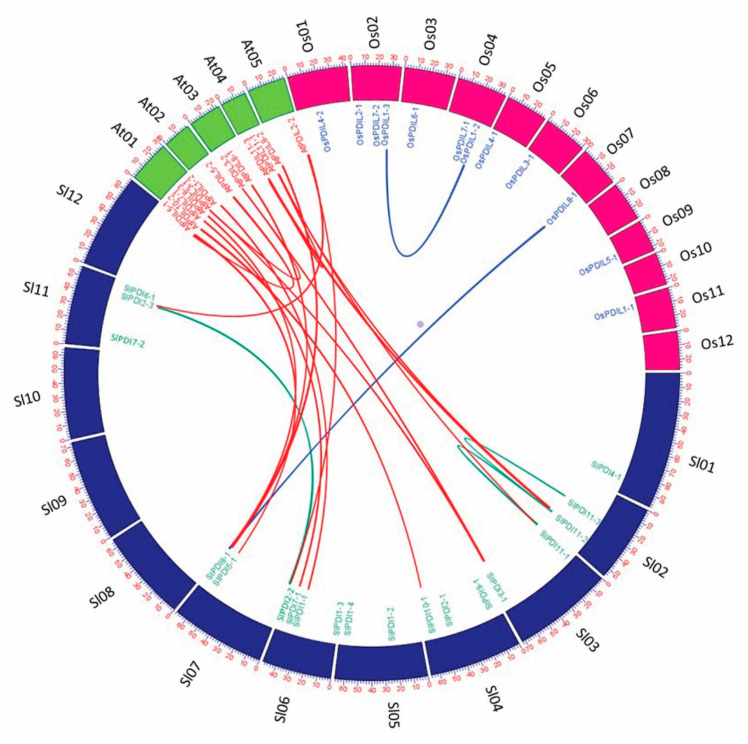
Microsynteny analysis of *PDI* genes among tomato, Arabidopsis, and rice. The chromosomes of the three species are represented in different colors: tomato, blue; Arabidopsis, green; and maize, pink. All chromosomes are drawn to scale (in Mbp).

SlPDI proteins displayed high sequence similarity within each group (Supplementary Table S1). To assess the extent and type of selective pressure imposed on the segmentally duplicated *SlPDI* gene family members, we calculated the Ka/Ks ratio for each pair of paralogous genes. We assigned a mode of selection based on Ka/Ks ratio values: a Ka/Ks ratio over 1 indicates accelerated evolution with positive selection; a Ka/Ks ratio of ~1 suggests neutral selection; a Ka/Ks ratio below 1 argues for the evolutionary constraints by negative or purifying selection. The Ka/Ks values of all three segmental duplicated gene pairs were below 1, suggesting that these genes experienced a strong purifying/negative selection, with slight variation after duplication. We estimated the divergence time for these paralogous gene pairs to have occurred 20.85 to 28.94 million years ago (MYA) (Table 3). In addition, we also constructed a comparative microsyntenic map to identify orthologous gene pairs of *PDI* genes between tomato, Arabidopsis, and rice to explore the evolutionary relationships across their genomes. We identified 14 pairs of orthologous between tomato and Arabidopsis, but only two pairs between tomato and rice, which agrees with the shorter evolutionary distance between tomato and the dicotyledonous model plant Arabidopsis than between tomato and the monocotyledonous model plant rice (Figure 6).

### 3.5. Analysis of Stress- and Hormone-Responsive Cis-Elements in the Promoter Regions of SlPDI Genes

*Cis*-regulatory elements in the upstream regions of genes play a pivotal role in the regulation of gene expression in response to various environmental stresses. Many stress responsive-genes have been reported to harbor a wide range of *cis*-acting elements in their promoter regions. Therefore, we utilized the web tool PlantCare database to identify putative phytohormone- and stress-responsive *cis*-regulatory elements in the *SlPDI* promoters. We observed various numbers of such *cis*-acting elements (Figure S5 and Supplementary Table S2), supporting the possible roles of *SlPDI* genes in abiotic stress tolerance. Of the abiotic stress-responsive *cis*-elements identified, ABA-responsive elements (ABRE) were present in the promoters of 14 genes, while TC-rich repeats with roles in defense and stress responses were detected in seven promoters. The low temperature responsive (LTR) element appeared in five promoters, while a MYB binding site (MBS) was found in four promoters. Finally, we identified the WUN-motif, which is involved in wound-responses, in one tomato *PDI* promoter. Despite having the putative cis-elements, some genes are not receptive to abiotic stress. For instance, five *PDI* genes, viz., *SlPDI1-2, SlPDI2-1, SlPDI5-1, SlPDI11-1* and *SlPDI11-3*, revealed the presence of the LTR element in their promoter regions, but *SlPDI2-1* did not exhibit the significant response to the cold stress application compared to the other four genes harboring the LTR element. Thus, these regulatory motifs present in the upstream of *PDI* genes should be validated experimentally to verify if they are functional regulatory elements.

### 3.6. Expression Analysis of Tomato PDI Genes in Various Organs

Analysis of organ-specific gene expression patterns can provide clues about the possible function of a gene over the course of development. Therefore, we investigated the expression pattern and relative levels of *PDI* transcripts across 12 tomato organs (i.e., leaves, stems, roots, flower buds, full blooming flowers, senescent flowers, 1 cm fruits, IM fruits, MG fruits, B fruits, B3 fruits, and B7 fruits) via RT-qPCR assay in the tomato cultivar Ailsa Craig. 

Relative to their expression levels in leaves, 16 *PDI* genes were expressed 2- to 25-fold more in stem tissues; similarly, 10 *PDI* genes were more highly expressed in 1 cm fruits (2- to 17-fold) and 11 showed a 2- to 12-fold increase in relative expression in breaker fruits. The expression of *SlPDI1-1*, *SlPDI1-2*, and *SlPDI1-3*, which all belonged to group I, was relatively high in stems, flower buds, and early stages of fruit development, such as the breaker stage, compared to other organs and later fruit ripening stages. By contrast, the mRNA transcripts of *SlPDI1-4* were more abundant in 1 cm fruits (~40-fold) in comparison with other organs. The expression of *SlPDI2-1* and *SlPDI2-3* was predominant in stems, 1 cm fruits, and breaker fruits, but their expression decreased at later ripening stages after the breaker stage. In contrast to its paralogous genes (*SlPDI2-1* and *SlPDI2-3*), *SlPDI2-2* showed flower-specific expression. We also determined that relative transcript levels of *SlPDI3-1* and *SlPDI5-1* reached their highest levels in 1 cm fruits, while transcripts of *SlPDI4-1*.

SlPDI6-1, and SlPDI11-3 were the most abundant in breaker fruits. The transcript levels of SlPDI7-1 and SlPDI8-1 were predominant in stems relative to other organs. SlPDI7-2 and SlPDI11-2 were preferentially expressed in floral buds and leaves, respectively.

When combined with our phylogenetic investigation, we noticed that several paralogous gene pairs belonging to the same phylogenetic groups shared similar expression patterns in the tested organs, while other paralogs exhibited different expression patterns. For example, *SlPDI2-1* and *SlPDI2-3* within group II were expressed to the highest levels in stems, whereas *SlPDI11-1, SlPDI11-2*, and *SlPDI11-3* from group XI were most highly expressed in distinct organs such as stems, leaves, and breaker fruits, respectively.

**Figure 7:**
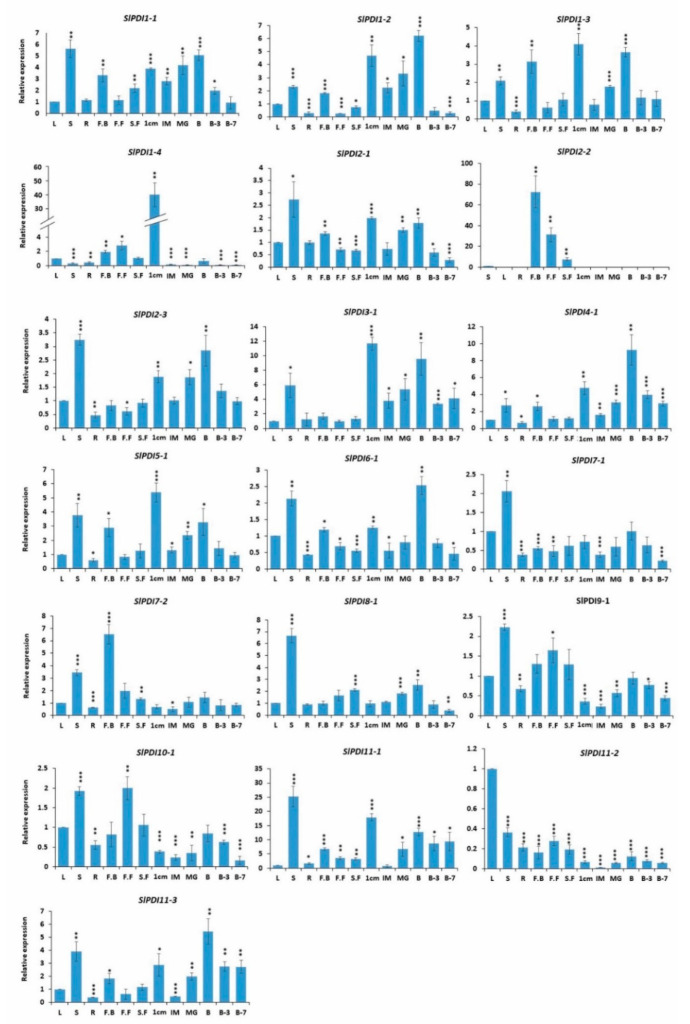
Quantitative real-time polymerase chain reaction (RT-qPCR) analysis of the expression profiles of *PDI* genes in 12 organs: leaves, roots, stems, flower buds (FB), full blooming flowers (FF), senescent flowers (SF), 1 cm fruits, immature fruits (IM), mature green fruits (MG), Breaker fruits (B), fruits 3 days after breaker stage (B3), and fruits 7 days after breaker stage (B7). *Le18S* (*18S rRNA*) expression levels were used as a reference. Error bars represent standard errors of the means of three replicates. The asterisk marks denote the significant difference as determined by *t*-test (* *p*-value ≤ 0.05, ** *p*-value ≤ 0.01 and *** *p*-value ≤ 0.001).

Broadly, in silico analysis of publicly available transcriptome deep-sequencing (RNA-seq) data from the tomato genome consortium [63] was largely consistent with the expression profiles generated by RT-qPCR in our study (Figure 7, Figures S4 and S5). Indeed, both analyses revealed that *SlPDI4-1* and *SlPDI5-1* reached their peak expression in breaker fruits and 1 cm fruits, respectively. Moreover, the transcript level of *SlPDI6-1* was highest in breaker fruits by RT-qPCR, whereas RNA-seq detected the highest level in roots. This variation in expression data could be in part due to the differences in data analysis approaches (Figure 7, Figures S4 and S5).

### 3.7. Expression Profiling of Tomato PDI Genes under Various Abiotic Stresses and Phytohormone Treatment

**Figure 8:**
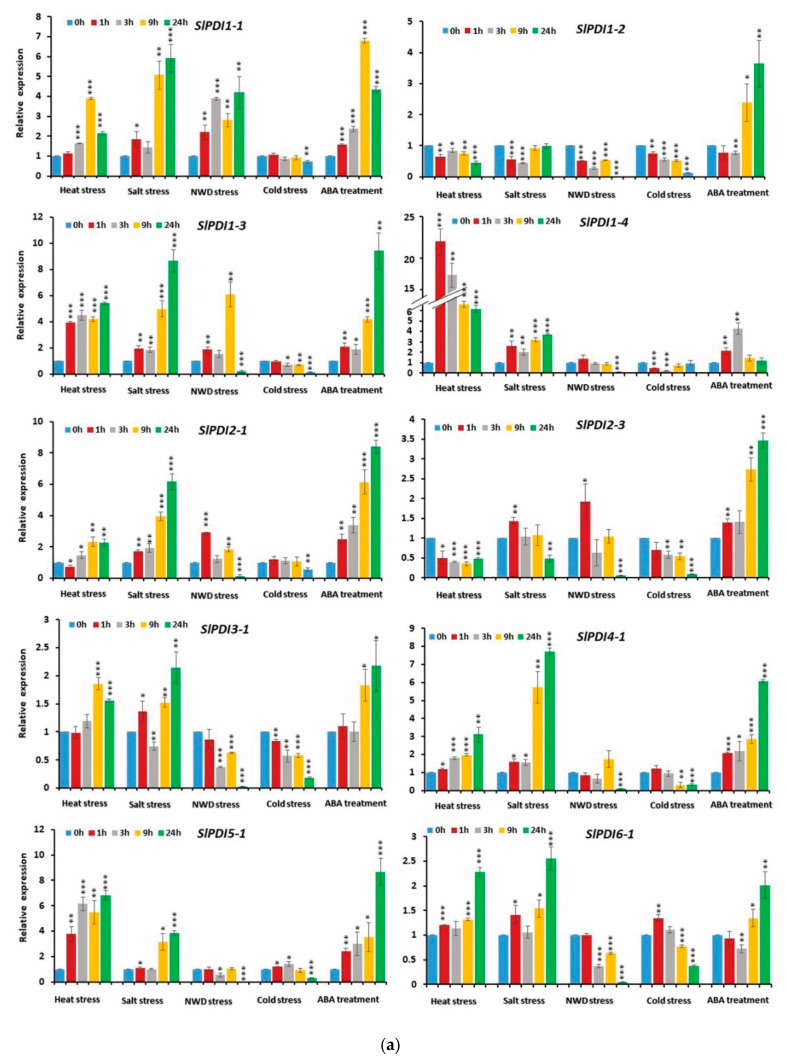

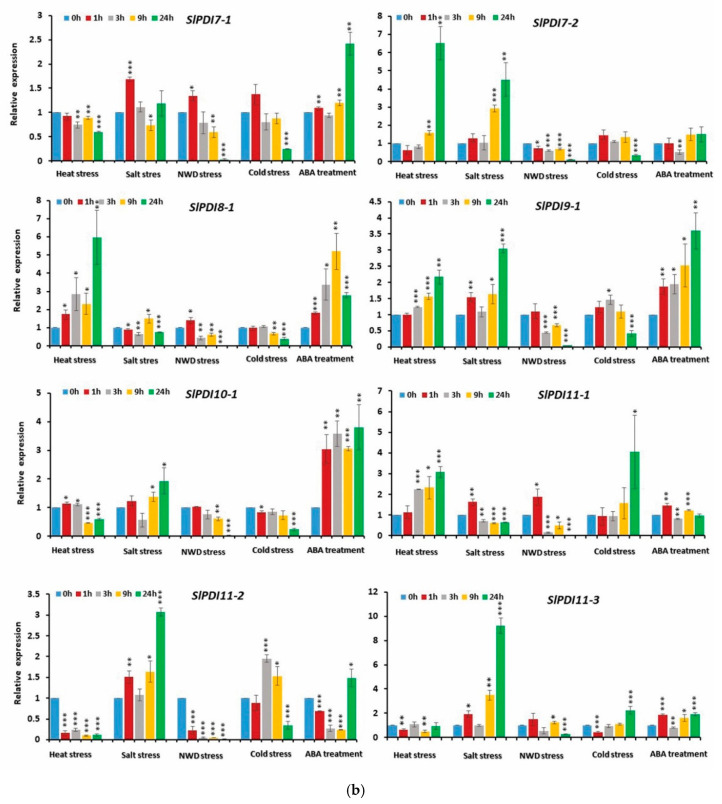
(**a**,**b**). Relative expression levels of *SlPDI* genes in response to various abiotic stresses, viz., heat stress, salt stress, nutrient and water deficit (NWD) stress and cold stress, and phytohormone treatment. Error bars indicate the standard errors of the means of three replicates. *, ** and *** represent the significant difference at *p*-value ≤ 0.05, ≤ 0.01 and ≤ 0.001, respectively.

To elucidate the putative function of tomato *PDI* gene family in response to abiotic stresses and ABA, we analyzed the expression patterns of *SlPDI* genes in leaves sampled at various time points (0 h, 1 h, 3 h, 9 h, and 24 h) after exposure to different abiotic stresses and ABA (Figure 8a,b and Figure S6a,b). The transcript level of *SlPDI2-2* remained constant in all samples. By contrast, the expression of many *PDI* genes responded strongly to these treatments at different time points (Figure 8a,b and Figure S6a,b).

Notably, 12 out of 19 *PDI* genes (*SlPDI1-1*, *SlPDI1-3*, *SlPDI1-4*, *SlPDI2-1*, *SlPDI3-1*, *SlPDI4-1*, *SlPDI5-1*, *SlPD6-1*, *SlPDI7-2*, *SlPDI8-1*, *SlPDI9-1*, and *SlPDI11-1*) were induced by heat treatment, with expression levels 1.8- to 22.5-fold higher than the control (0 h) at various time points (Figure 8a,b and Figure S6a,b). Of these, four genes (*SlPDI1-3*, *SlPDI1-4*, *SlPDI5-1*, and *SlPDI8-1*) were highly up-regulated throughout the stress period. The expression of another four genes (*SlPDI1-1*, *SlPDI2-1*, *SlPDI4-1*, and *SlPDI11-1*) initially showed no response 1 h after heat exposure compared to the control, but subsequently rose at later time points. We also observed that three genes (*SlPDI3-1*, *SlPDI7-2*, and *SlPDI9-1*) were up-regulated by >1.5- to 6.5-fold at later time points of heat treatment relative to the control. By contrast, a few *PDI* genes (*SlPDI1-2*, *SlPDI2-3*, *SlPDI10-1*, and *SlPDI11-2*) were down-regulated (>2- to >8-fold) in heat-treated samples compared to the control. Expression of *SlPDI7-1* and *SlPDI11-3* was almost unchanged in response of heat treatment at most time points, but decreased slightly (>1.6-fold) at 24 h and 9 h, respectively.

Salt treatment also caused remarkable changes in the expression of tomato *PDI* genes: 18 *PDI* genes were differentially expressed across several time points following exposure to salt stress (Figure 8a,b). Most *PDI* genes were up-regulated in response to salt stress. Of the up-regulated *PDI* genes, *SlPDI1-3*, *SlPDI1-4*, *SlPDI2-1*, and *SlPDI4-1* transcript levels were elevated at all time points. In addition, *SlPDI1-1*, *SlPDI9-1*, *SlPDI11-2* and *SlPDI11-3* were highly expressed at most time points. The expression levels of *SlPDI5-1* and *SlPDI7-2* initially did not change during early time points, but subsequently increased at later time points. *SlPDI3-1*, *SlPDI6-1*, and *SlPDI10-1* were up-regulated by >1.5- to 2.5-fold at later time points. *SlPDI7-1* and *SlPDI11-1* were more highly expressed (>1.6-fold over control) specifically 1 h after onset of treatment. By contrast, the expression levels of three genes (*SlPDI1-2*, *SlPDI2-3*, and *SlPDI8-1*) were minimally down-regulated from >1.5- to >2.2-fold upon exposure to salt stress. The transcript levels of *SlPDI8-1* decreased only slightly (1.5-fold relative to the control) 3 h after salt treatment. 

The expression levels of 18 (out of 19) *PDI* genes were altered by nutrient and water deficit (NWD) treatment, most *PDI* genes being repressed (Figure 8a,b and Figure S6a,b). Only five genes (*SlPDI1-1*, *SlPDI1-3*, *SlPDI2-1*, *SlPDI2-3*, and *SlPDI11-1*) out of these 18 displayed up-regulated expression levels (>1.8- to 9.5-fold), whereas transcript levels for the remaining 13 *PDI* genes declined (1.5- to 80-fold relative to the control) at various time points of NWD treatment. 

Cold stress lowered the expression of 14 *PDI* genes, particularly at the last time point following cold exposure (Figure 8a,b and Figure S6a,b). However, *SlPDI1-1* transcript levels did not respond to the cold treatment. Intriguingly, three paralogous genes (*SlPDI11-1*, *SlPDI111-2*, and *SlPDI11-3*) within group XI displayed higher expression levels in response to cold stress.

ABA treatment triggered the differential expression of *PDI* genes at various time points (Figure 8a,b and Figure S6a,b). Fourteen *PDI* genes were significantly induced by ABA, whereas four *PDI* genes (namely, *SlPDI7-2*, *SlPDI11-1*, *SlPDI11-2*, and *SlPDI11-3*) showed little change in response to ABA application. 

## 4. Discussion

In this study, we identified 19 *SlPDI* genes in tomato, clearly confirming that the size of the *PDI* gene family varies among different plant species. Tomato had more *PDI* genes than poplar (12), maize (12) or *B. distachyon* (11) but fewer than Chinese cabbage (32) or Arabidopsis (21) (Figure 1). As relatively small *PDI* gene families are found in plants with genomes that are both relatively small, such as *B*. *distachyon* (335 Mbp), and relatively large, such as maize (2300 Mbp), *PDI* gene family size and genome size do not appear to be correlated. From an evolutionary point of view, gene duplication events generate additional copies of genes that enable plants to adapt and survive in response to varying environmental conditions [64,65]. We detected a number of segmentally duplicated gene pairs in the tomato *PDI* gene family: *SlPDI2-2/SlPDI2-3*, *SlPDI11-1/SlPDI11-2*, and *SlPDI11-2/SlPDI11-3* (Figure 5 and Figure S3). These duplicated genes shared similar motif compositions and exon/intron organization, suggesting that the 19 *SlPDI* genes likely originated from an initial set of 14 ancestral genes. Additionally, the duplication events detected for *SlPDI* genes were group-specific, which indicated that segmental duplications rather than tandem duplications drove the expansion of the *SlPDI* gene family.

Variations in exon-intron structures are a hallmark of the evolution of many gene families and also contribute to their structural diversity [66]. We observed similar exon-intron structures and motif distributions among the more closely related *PDI* genes in the phylogenetic tree, while we detected multiple differences between members belonging to different phylogenetic groups (Figure S1 and Figure 4). These findings suggest functional similarity among *PDI* genes within the same phylogenetic group and a possible explanation for the functional diversification of *SlPDI* genes over the course of evolution.

The phylogenetic analysis validated the 11 groups of PDI proteins falling into 4 clades in tomato, which was in agreement with the findings of Kayum et al. (2017) (Figure 1) [22]. The high homology between the PDI proteins in tomato and those in poplar, Arabidopsis, and Chinese cabbage indicated their close evolutionary relationships as well as their functional similarities in dicotyledonous plants. In accordance with the phylogenetic classification, we observed that two distinct subgroups comprised PDI and PDIL members from dicots (tomato, poplar, Arabidopsis, and Chinese cabbage) and monocots (*B. distachyon* and maize), which is consistent with a previous report by Zhu et al. (2014) [33].

Variable abundance of transcripts across organs may help predict functional divergence of their corresponding genes. In tomato, as in other species, we established differential expression of *PDI* genes in various organs, suggesting that functional diversity may have emerged and that individual *PDI* genes may play distinct regulatory roles in growth and development (Figure 7 and Figure S4). Previous reports had detected the expression of *PDI* family members in all tissues examined, hinting at a lack of organ-specificity, which is in agreement with our results [28,33]. However, transcripts for several Chinese cabbage *PDI* genes were not detected in all organs tested, suggesting the potential for functional variation in *PDI* genes in different species [22]. Tomato *PDI* genes exhibited diverse transcript levels in different organs (Figure 7 and Figure S4). For instance, several *PDI* family members, such as *SlPDI7-1*, *SlPDI8-1*, and *SlPDI11-2*, were predominantly expressed in vegetative organs, while others, such as *SlPDI1-1*, *SlPDI1-2*, *SlPDI1-4*, *SlPDI2-2* and *SlPDI7-2*, were highly expressed in reproductive organs (i.e., fruits and flowers), suggesting their specific involvement in these organs and developmental stages in tomato. 

The stem supports the above-ground parts of the plant and mediates long-range transport of water and nutrients, thereby supporting plant growth under normal and adverse conditions. Many *PDI* genes, (*SlPDI1-1*, *SlPDI2-1*, *SlPDI2-3*, *SlPDI6-1*, *SlPDI8-1*, *SlPDI9-1*, *SlPDI10-1*, and *SlPDI11-1*) were predominantly expressed in the stem (Figure 7 and Figure S4), implying that they may be involved in stem development, long-distance translocation of water and nutrients, and/or stress tolerance. Intriguingly, we observed that only one gene (*SlPDI11-2*) was highly expressed in leaves, in contrast to its segmentally duplicated sister genes (*SlPDI11-1* and *SlPDI11-3*), which showed higher expression in stem and fruit, respectively. In addition, *SlPDI2-3* was highly expressed in the stem and breaker fruits, while its duplicated gene (*SlPDI2-2*) was expressed only in floral organs, especially in floral buds (Figure 7 and Figure S4), suggesting their functional diversification after gene duplication. The induction and development of floral buds marks the transition from the vegetative phase to the reproductive phase, which is a requisite for fruit set and a critical factor that defines crop yield [67]. The higher transcript levels of *SlPDI7-2* in flower buds and of *SlPDI10-1* in full blooming flowers suggest that their functions are related to floral bud formation and flower development (Figure 7 and Figure S4). 

Tomato, a model system for fleshy fruit plants, has been extensively studied with regard to the development and ripening of climacteric fruits. The development of tomato fruits occurs in two major steps: (1) fruit enlargement, which can be further divided into cell division phase and cell expansion phase, and (2) fruit ripening [68,69]. Many *PDI* genes were highly expressed in 1-cm fruits and during fruit-ripening stages (Figure 7 and Figure S4). *SlPDI1-3* and *SlPDI3-1* were preferentially expressed in both 1-cm fruits and at the fruit breaker stage (Figure 7 and Figure S4), indicating that they may be important for cell division and fruit ripening. The higher expression levels of *SlPDI1-4* and *SlPDI5-1* in 1-cm fruits also revealed its possible involvement in the cell division phase of tomato fruit development (Figure 7 and Figure S4). The predominant expression of *SlPDI2-3*, *SlPDI4-1*, *SlPDI6-1*, and *SlPDI11-3* during the breaker stage suggests they have active roles in the fruit-ripening process in tomato (Figure 7 and Figure S4). The role of *PDI* gene family members during fruit development has not previously been studied in any vegetable species: our study, therefore, constitutes the first analysis of *PDI* gene expression patterns at different fruit developmental stages. Our results suggest possible functions for tomato *PDI* genes during initial fruit development and ripening, and lay the foundation for further functional validation of this gene family in tomato.

Various environmental stresses disturb plant physiological processes. More specifically, multiple abiotic stresses result in the aggregation of unfolded or denatured proteins, resulting in the disruption of normal cellular function [70,71]. Thus, among the myriad mechanisms used by plants to adapt to adverse environmental conditions is the reconstitution of active proteins from denatured or misfolded proteins by PDIs. The involvement of PDIs in plant stress responses has been corroborated by a number of reports that revealed the up-regulation of *PDI* genes in various plant species, including Arabidopsis, *B. distachyon*, and wheat [23,32,33]. The role of *PDI* genes in abiotic stress tolerance has also been highlighted by the higher expression levels of four *PDI* genes in Chinese cabbage in a cold-tolerant cultivar during exposure to cold stress. The expression of many other *PDI* genes was also responsive to additional environmental stresses, such as salt, drought, and ABA treatment [22].

In support of previous findings, tomato *PDI* genes showed differential expression in response to various stress stimuli (Figure 8a,b and Figure S6a,b). Twelve *PDI* genes were markedly up-regulated following heat stress (Figure 8a,b and Figure S6a,b), suggesting that elevated temperatures may trigger the chaperone activity of PDI proteins or the coordinated expression of *PDI* genes with other chaperone-encoding genes to reactivate heat-denatured proteins and to protect cells from thermal damage. This finding fits well with a recent report whereby transgenic rice plants overexpressing the *MtPDI* gene from the extremophilic bacterium *Methanothermobacter thermautotrophicus* exhibited robust tolerance to heat stress [72]. In addition, differential display analysis of heat-inducible genes in the leaves of a semiarid grass (*Aneurolepidium chinense* [Trin.] kitag), which can tolerate heat, drought, and high salinity conditions, revealed *PDI* genes that were induced by heat stress [73]. The presence of two active thioredoxin motifs and a putative ER localization motif in the PDI proteins encoded by the heat-induced genes *SlPDI1-1*, *SlPDI1-3*, *SlPDI1-4*, *SlPDI2-1*, *SlPDI4-1*, and *SlPDI5-1* also suggest the active involvement of these proteins in correcting denatured proteins or protein misfolding in the ER during heat stress (Table 1 and Table 2 and Figure 8a and Figure S6a). 

The possible role of *PDI* genes in response to salt stress was previously suggested by transcript profiling in leaves of salt-tolerant grapevine rootstocks [74]. In support of this previous report, the expression of thirteen tomato *PDI* genes, including *SlPDI1-1, SlPDI1-3, SlPDI1-4*, *SlPDI2-1, SlPDI4-1*, and *SlPDI5-1*, were also sharply elevated by salt treatment (Figure 8a and Figure S6a). Thus, we conclude that these six genes may play a role in tomato tolerance to both heat and salt stresses. Besides these six genes, *SlPDI3-1*, *SlPDI6-1*, *SlPDI7-2*, and *SlPDI9-1* also showed increased expression in plants subjected to heat and salt stresses, underscoring their potential role in heat and salt tolerance (Figure 8a,b and Figure S6a,b).

Cold and nutrient and water deficit (NWD) stress conditions affected the expression of relatively few *PDI* genes, increasing transcript levels for three and five of the *PDI* genes, respectively (Figure 8a,b and Figure S6a,b). These results suggest the preferential function of most tomato *PDI* genes in adaptive responses to heat and salt stress rather than to NWD or cold. Intriguingly, the expression of both segmentally duplicated gene pairs (i.e., *SlPDI11-1*/*SlPDI11-2* and *SlPDI11-2*/*SlPDI11-3*) in group XI increased under cold stress, indicating their redundant functions in cold tolerance. In light of their expression profiles under various abiotic stresses, *SlPDI11-1* may be associated with low- and high-temperature stresses, whereas *SlPDI11-2* and *SlPDI11-3* may be involved in tolerance to cold and salt stresses (Figure 8a,b and Figure S6a,b). 

ABA is a stress phytohormone that plays vital regulatory roles in response to heat, salinity, and drought [75,76]. The function of *PDI* genes in abiotic stress tolerance may also be related to ABA signaling directly, as previously hinted at by the up-regulation of many *PDI* genes in Chinese cabbage and *B. distachyon* in response to ABA treatment [22,33]. In the present study, ABA treatment induced the expression of 14 tomato *PDI* genes (Figure 8a,b and Figure S6a,b), which is consistent with previous reports that suggested a role for tomato *PDI* genes in the regulation of abiotic stress tolerance through an ABA-dependent pathway.

We identified several stress-associated *cis*-elements in the promoters of tomato *PDI* genes, consistent with the stress-responsiveness of these genes (Figure S7 and Supplementary Table S2). These observations are also consistent with previous findings of stress-related *cis*-acting elements in the promoter regions of stress-responsive *PDI* genes in Chinese cabbage and *B. distachyon*. The evidence provided here lays the foundation for additional functional characterization of *SlPDI* genes to gain a better understanding of the molecular mechanisms governed by *PDI* genes during abiotic stress tolerance in tomato.

## 5. Conclusions

PDI proteins participate in plant development and stress tolerance by ensuring the proper folding of misfolded or denatured proteins. This study represents the first comprehensive characterization of *PDI* genes in tomato by genome-wide bioinformatics identification and expression analysis in different organs and under various abiotic stresses. The 19 *PDI* genes identified here clustered into 11 groups belonging to four clades, reflecting their domain organizations. Many *PDI* genes (*SlPDI1-1*, *SlPDI1-2*, *SlPDI1-3*, *SlPDI1-4*, *SlPDI2-3*, *SlPDI3-1*, *SlPDI4-1*, *SlPDI5-1*, *SlPDI6-1*, *SlPDI11-1*, and *SlPDI11-3*) were preferentially expressed in the stem and fruits, suggesting their possible involvement in the development of these organs. The predominant expression of *SlPDI2-2*, *SlPDI7-2* and *SlPDI11-2* in floral buds and leaves suggested their role in floral and leaf development, respectively. Additionally, transcript levels of tomato *PDI* genes were differentially regulated by exposure to abiotic stresses and to the stress phytohormone ABA. Most *PDI* genes, particularly *SlPDI1-1*, *SlPDI1-3*, *SlPDI1-4*, *SlPDI2-1*, *SlPDI4-1*, and *SlPDI5-1*, were induced by various abiotic stresses and ABA treatment, suggesting that they may function in the adaptation of tomato to these stresses via an ABA-dependent pathway. Together, the information obtained in this study will provide a better understanding of the structures and functions of *SlPDI* genes, many of which may be potential candidate genes for developing tomato cultivars with improved fruit quality and stress tolerance via marker-assisted back crossing (MAB) or transgenesis.

## Figures and Tables

**Table 1:** List of identified tomato *PDI* gene family members with their corresponding encoded protein information.

Gene Name	Locus Name	ORF (bp)	Chrom. (Strand)	No. of Introns	Proteins			Subcellular Localization
					Length (aa)	MW (kDa)	PI	
*SlPDI1-1*	Solyc06g060290	1557	C06 (−strand)	9	438	58.11	5.02	Endoplasmic reticulum
*SlPDI1-2*	Solyc05g018700	1185	C05 (−strand)	10	496	44.47	5.10	Endoplasmic reticulum
*SlPDI1-3*	Solyc06g005940	1500	C06 (−strand)	9	499	55.74	5.18	Endoplasmic reticulum
*SlPDI1-4*	Solyc05g056400	1590	C05 (+strand)	9	537	59.01	4.96	Endoplasmic reticulum
*SlPDI2-1*	Solyc04g049450	1692	C04 (+strand)	11	563	63.10	4.65	Endoplasmic reticulum
*SlPDI2-2*	Solyc06g075210	1644	C06 (+strand)	11	554	61.66	4.91	Endoplasmic reticulum
*SlPDI2-3*	Solyc11g069400	1743	C11 (−strand)	11	580	64.58	4.49	Endoplasmic reticulum
*SlPDI3-1*	Solyc03g120720	1617	C03 (+strand)	11	538	60.68	4.81	Endoplasmic reticulum
*SlPDI4-1*	Solyc01g100320	1080	C01 (−strand)	10	359	39.45	5.43	Endoplasmic reticulum
*SlPDI5-1*	Solyc07g049450	1299	C07 (−strand)	8	432	47.07	5.58	Extracellular
*SlPDI6-1*	Solyc11g069690	450	C11 (+strand)	3	125	16.92	4.83	Extracellular
*SlPDI7-1*	Solyc06g065320	1329	C06 (+strand)	4	326	49.69	5.20	Plasma membrane
*SlPDI7-2*	Solyc11g019920	1308	C11 (+strand)	4	435	49.30	5.09	Plasma membrane
*SlPDI8-1*	Solyc07g064250	1446	C07 (−strand)	14	481	53.98	6.62	Plasma membrane
*SlPDI9-1*	Solyc04g007610	1545	C04 (−strand)	11	514	57.66	7.76	Extracellular
*SlPDI10-1*	Solyc04g074240	972	C04 (−strand)	3	323	35.56	8.74	Plasma membrane
*SlPDI11-1*	Solyc03g031620	885	C03 (−strand)	4	456	50.82	6.78	Chloroplast
*SlPDI11-2*	Solyc02g080640	1044	C02 (+strand)	3	461	48.20	6.08	Chloroplast
*SlPDI11-3*	Solyc02g032860	660	C02 (+strand)	4	456	50.70	6.40	Chloroplast

Abbreviations: ORF, open reading frame; bp, base pair; aa, amino acid; MW, molecular weight; kDa, kilo Dalton; PI, iso-electric point.

**Table 2:** Structural and functional characteristics of tomato PDI proteins.

Name	Signal Peptide	Trans-Membrane	Domain Organization	Active Site Motif	Conserved Charge Pair Sequence	Conserved Arginine	C-Terminal Signal
SlPDI1-1	No	No	a-b-a’	CGHC, CGHC	E56-K90, E399-K432	R130	-RCYC
SlPDI1-2	1-17	No	a-b-a’	CGYC, CRYC	Q44-K78, E387-K420	R118, R457	-KDEL
SlPDI1-3	1-23	No	a-b-a’	CGHC, CGHC	E50-K84, E393-K426	R124	-FRGL
SlPDI1-4	1-21	512-534	a-b-a’-t	CGHC, CGHC	E49-K83, E392-K425	R123, R462	-ISCN
SlPDI2-1	1-24	No	a-b-b’-a’	CGHC, CGHC	E105-K137, E444-K477	R173, R515	-KDEL
SlPDI2-2	1-27	No	a-b-a’	NGYC, CRQC	E102-K134, E443-K476	R170, P514	-RDEL
SlPDI2-3	1-26	No	a-b-b’-a’	CGHC, CGHC	E121-K153, E460-K493	R189, R531	-KDEL
SlPDI3-1	1-29	No	a-c-b-a’	CARS, CITC	L103-K137, E444-R477	F173, S514	-RDEL
SlPDI4-1	No	No	a°-a-D	CGHC, CGHC	E47-K80, E166-N199	R118, R237	-ATFA
SlPDI5-1	1-22	No	a°-a-b	CGHC, CGHC	E52-A83, E180-H211	R120, R249	-KDEL
SlPDI6-1	No	No	a	CKHC	K51-Q84	R121	-TERY
SlPDI7-1	No	No	a-c-b	CGHC	D18-K52	R88	-TETY
SlPDI7-2	1-25	378-400	a-b-t	CGHC	D57-K91	R127	-EKID
SlPDI8-1	No	No	e-a-f	CYWS	N164-K203	R249	-GKNF
SlPDI9-1	1-22	478-500	a-g-t	CPAC	E71-R109	Q147	-RSWN
SlPDI10-1	1-22	No	a	CPFS	L92-I123	R159	-SSTH
SlPDI11-1	No	No	h-a’	CRFC	V370-R404	R443	-NALR
SlPDI11-2	No	No	h-a’	CQFC	V375-R409	R448	-NALR
SlPDI11-3	No	No	h-a’	CQFC	V370-R404	R443	-NALR

**Table 3:** Estimated Ka/Ks ratios of the segmentally duplicated *SlPDI* genes with their divergence time in tomato.

Duplicated Gene Pairs	Ka	Ks	Ka/Ks	Duplication Type	Types of Selection	Time (MYA)
*SlPDI2-2/SlPDI2-3*	0.197166	0.868192	0.227099	Segmental	Purifying selection	28.94
*SlPDI11-1/SlPDI11-2*	0.095269	0.62544	0.152323	Segmental	Purifying selection	20.85
*SlPDI11-2/SlPDI11-3*	0.072036	0.628726	0.114574	Segmental	Purifying selection	20.96

Ks the number of synonymous substitutions per synonymous site, Ka the number of non-synonymous substitutions per nonsynonymous site, MYA million years ago.

## Supplementary Materials

The following are available online at https://www.mdpi.com/2073-4425/12/1/23/s1, Figure S1. Schematic representation of the exon-intron organization of tomato *PDI* genes. Figure S2: Overview of conserved motifs of PDI proteins analysed by MEME online tool. Figure S3. Gene duplication analyses of *PDI* genes in the tomato genome. Figure S4. RT-qPCR analysis of the expression profiles of *PDI* genes in 12 organs. Figure S5. Relative expression levels of *SlPDI* genes based on RNA-seq data from the Tomato Genome Consortium (2012). Figure S6 (a,b). Relative expression levels of *SlPDI* genes in response to various abiotic stresses and phytohormone treatment. Figure S7. Predicted *cis*-regulatory elements in the promoters of tomato *PDI* genes. Table S1. Sequence identity among the 19 PDI proteins of tomato. Table S2. Putative *cis*-elements in tomato *PDI* genes identified by using PlantCARE database. Table S3. List of the PDI protein sequences in *Solanum lycopersicum*, *Arabidopsis thaliana*, *Brassica rapa*, *Populus trichocarpa*, *Zea mays* and *Brachypodium distachyon*. Table S4. Primers used for qRT-PCR assay to analyse the expression of the SlPDI genes.
