# Supplementary material for: Genome-Wide Identification and Expression Profiling of the PDI Gene Family Reveals Their Probable Involvement in Abiotic Stress Tolerance in Tomato (Solanum lycopersicum L.)
Source: Genes (Basel). 2020 Dec 25;12(1):23. doi: 10.3390/genes12010023 (PMC7824348; doi:10.3390/genes12010023)
Supplement: Supplementary file 1 [file genes-12-00023-s001.pdf]

Supplementary Materials: Genome-wide identification and expression profiling of the *PDI* gene family reveals their probable involvement in abiotic stress tolerance in tomato (*Solanum lycopersicum* L.)

Antt Htet Wai , Muhammad Waseem, A.B.M.M.M.Khan, Ujjal Kumar Nath, Do-jin Lee, Sang-Tae Kim, Chang-Kil Kim and Mi-Young Chung

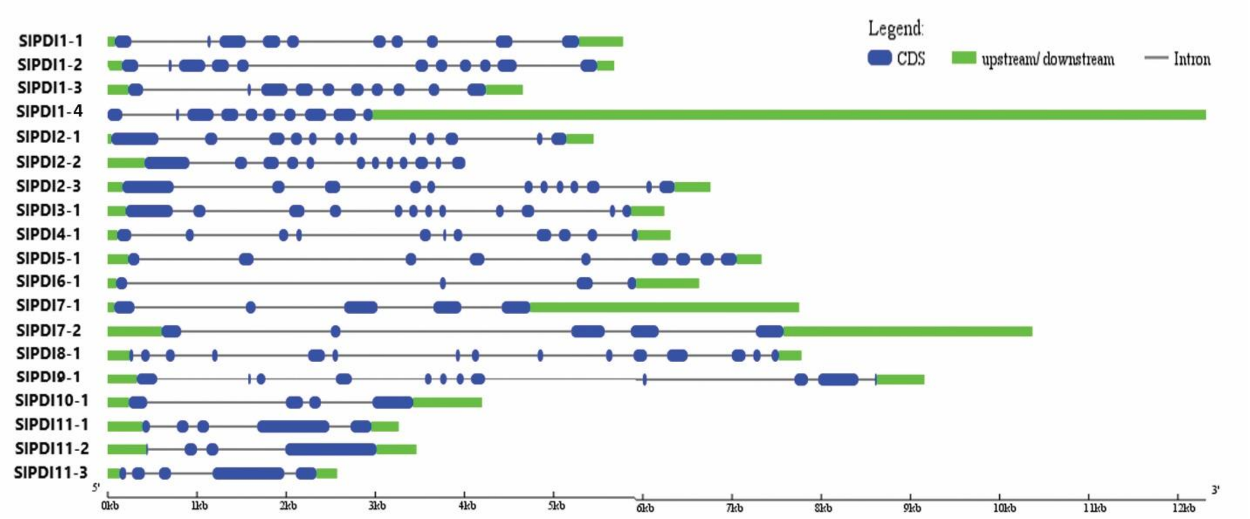

Figure S1. Schematic representation of the exon-intron organization of the tomato *PDI* genes. Blue boxes indicate exons, black lines indicate introns, and green boxes represent untranslated regions. The scale below can be used to estimate the lengths of exons/introns.

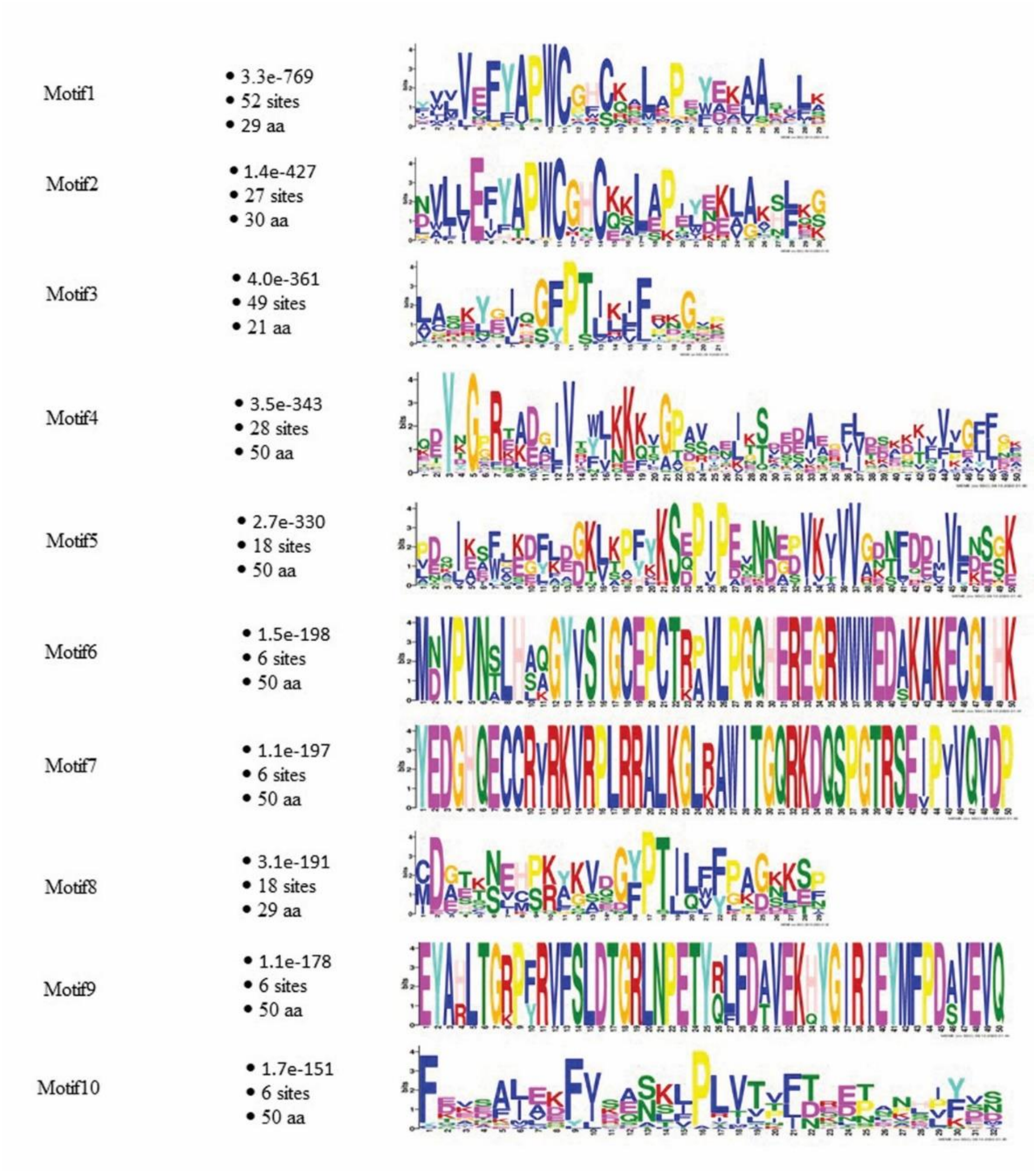

Figure S2. Overview of conserved motifs of SlPDI proteins analysed by MEME online tool

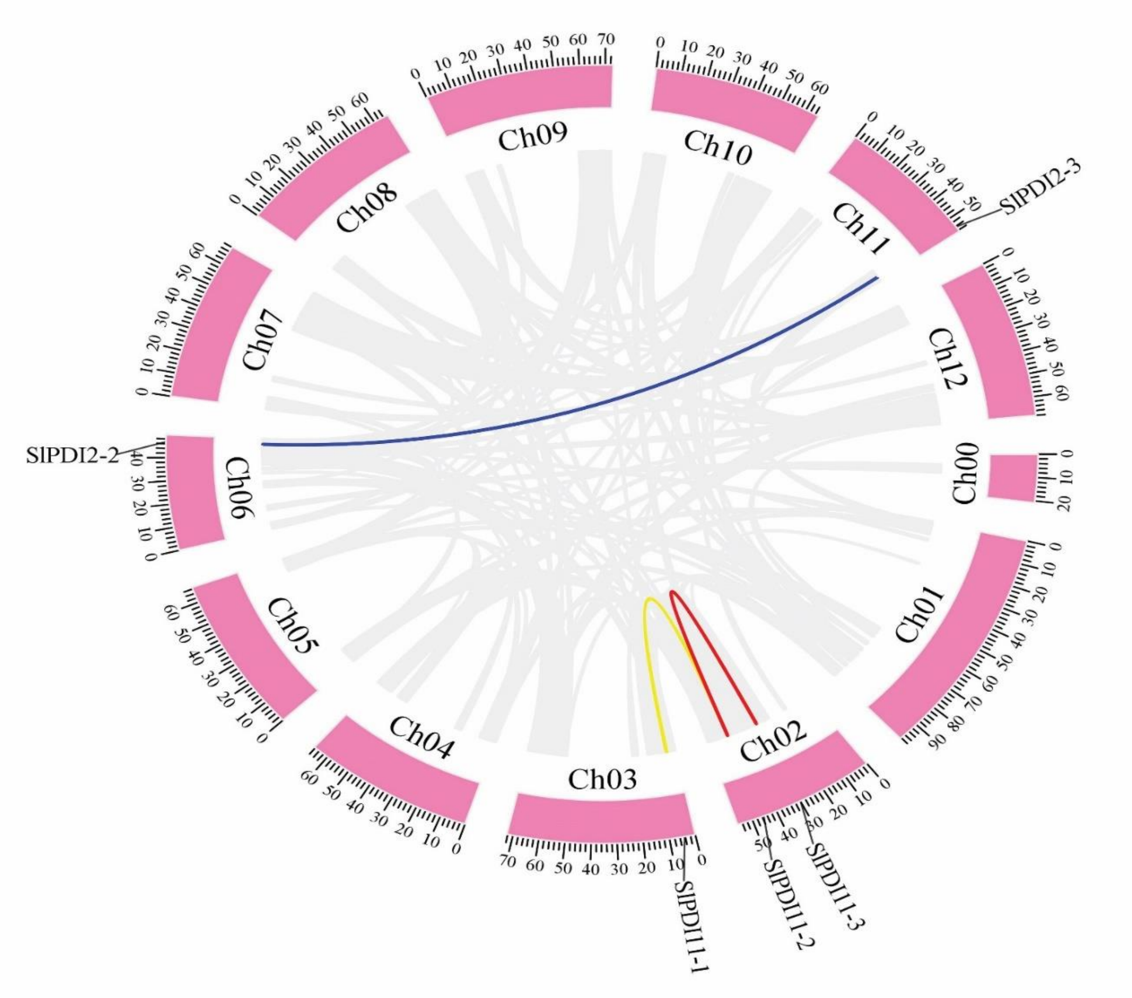

Figure S3. Gene duplication analysis of *PDI* genes in the tomato genome. Chromosome numbers and gene positions are indicated. The chromosome size and scale is in Megabase pairs (Mbp). Blue, yellow, and red lines represent segmentally duplicated *SlPDI* gene pairs, namely *SlPDI2-2*/*SlPDI2-3*, *SlPDI11-1*/*SlPDI11-2*, and *SlPDI11-2*/*SlPDI11-3*, respectively.

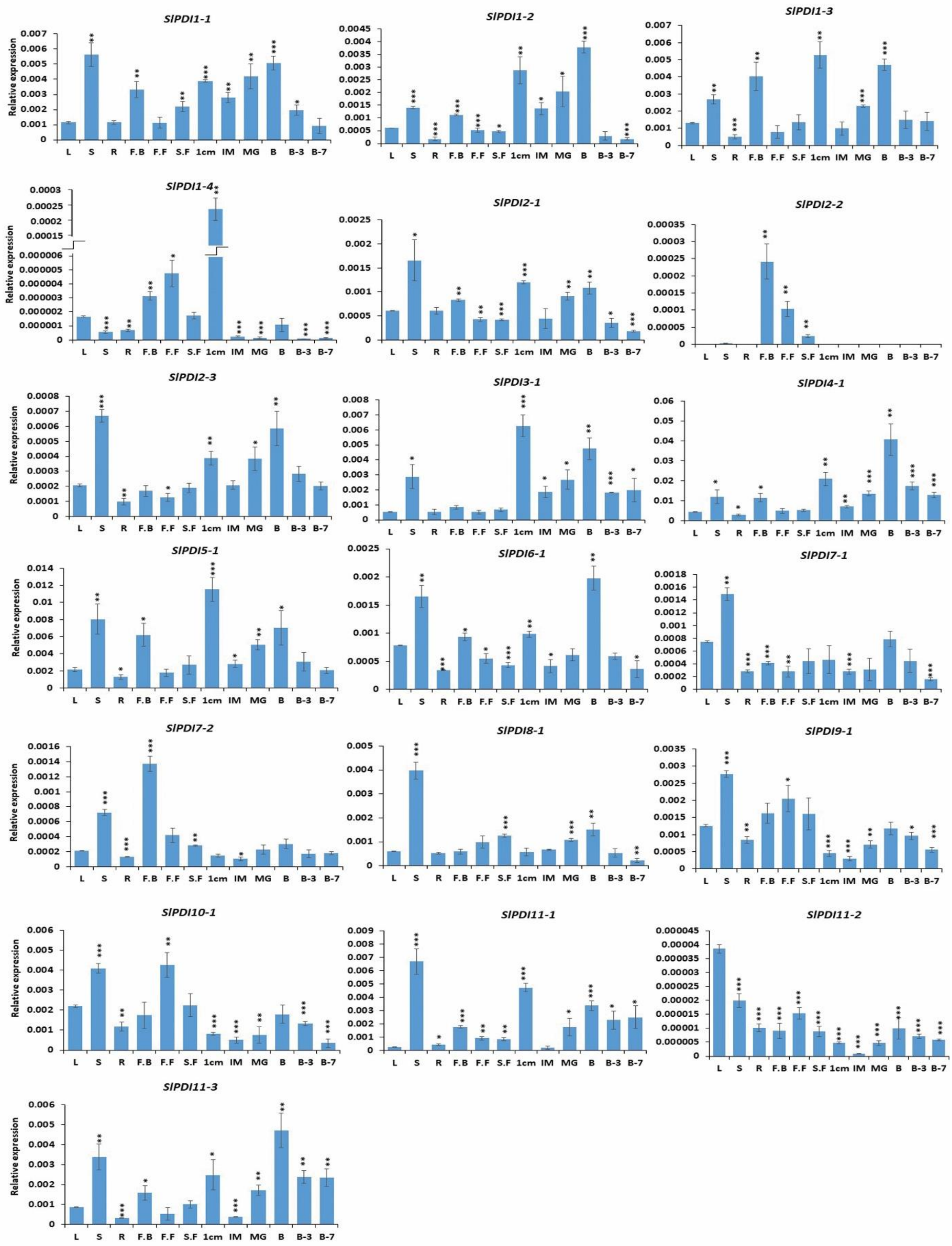

Figure S4. RT-qPCR analysis of the expression profiles of *PDI* genes in 12 organs: leaves, roots, stems, flower buds (FB), full blooming flowers (FF), senescent flowers (SF), 1 cm fruits, immature fruits (IM), mature green fruits (MG), Breaker fruits (B), fruits 3 days after breaker stage (B3), and fruits 7 days after breaker stage (B7). *Le18S* (<u>*18S rRNA*</u>) expression levels were used as a reference. Error bars represent standard errors of the means of three replicates. The asterisk marks denote the significant difference as determined by *t*-test (* P-value ≤ 0.05, ** P-value ≤ 0.01 and *** P-value ≤ 0.001).

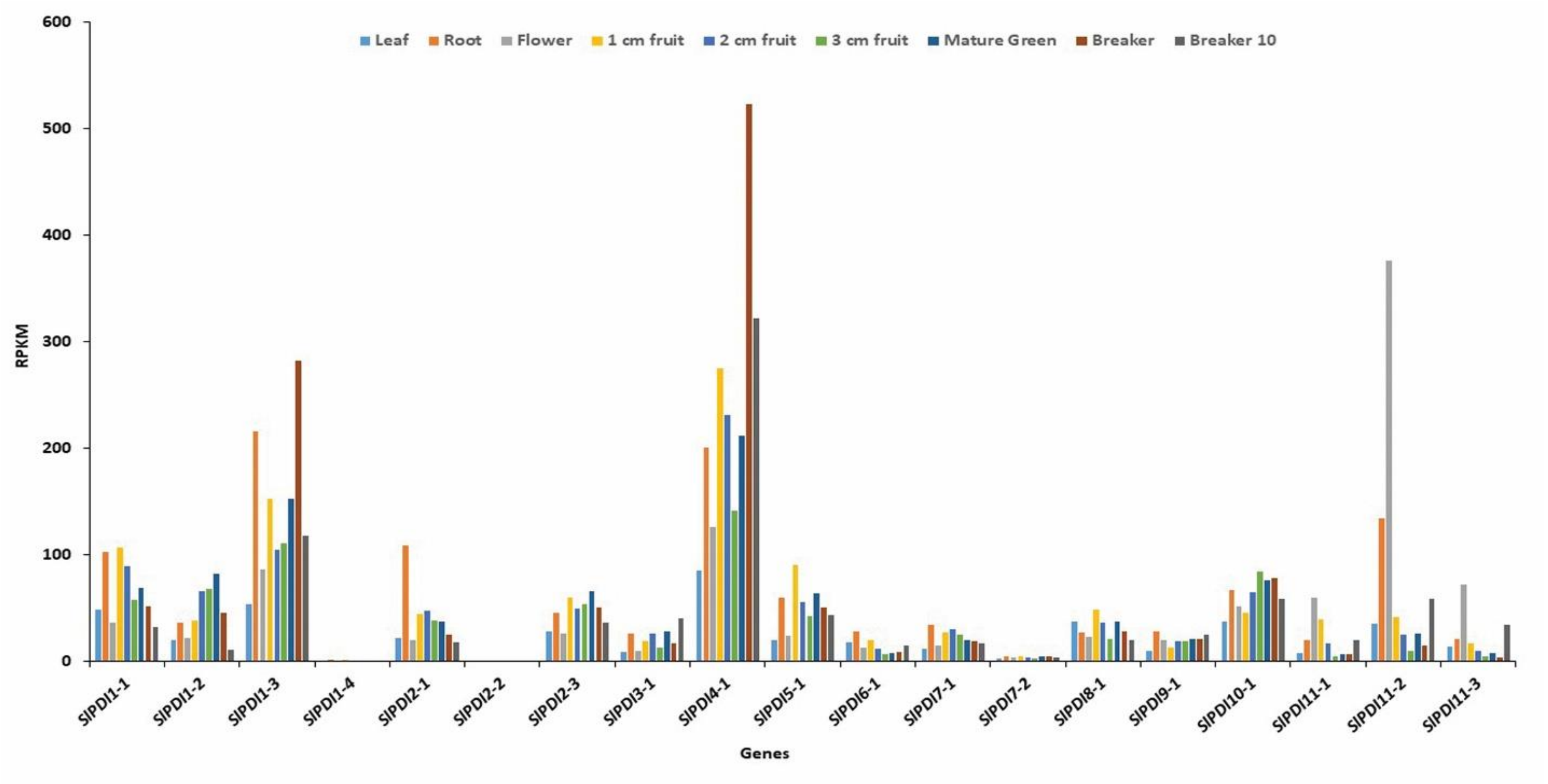

Figure S5**.** Relative expression levels of *SlPDI* genes based on RNA-seq data from the Tomato Genome Consortium (2012).

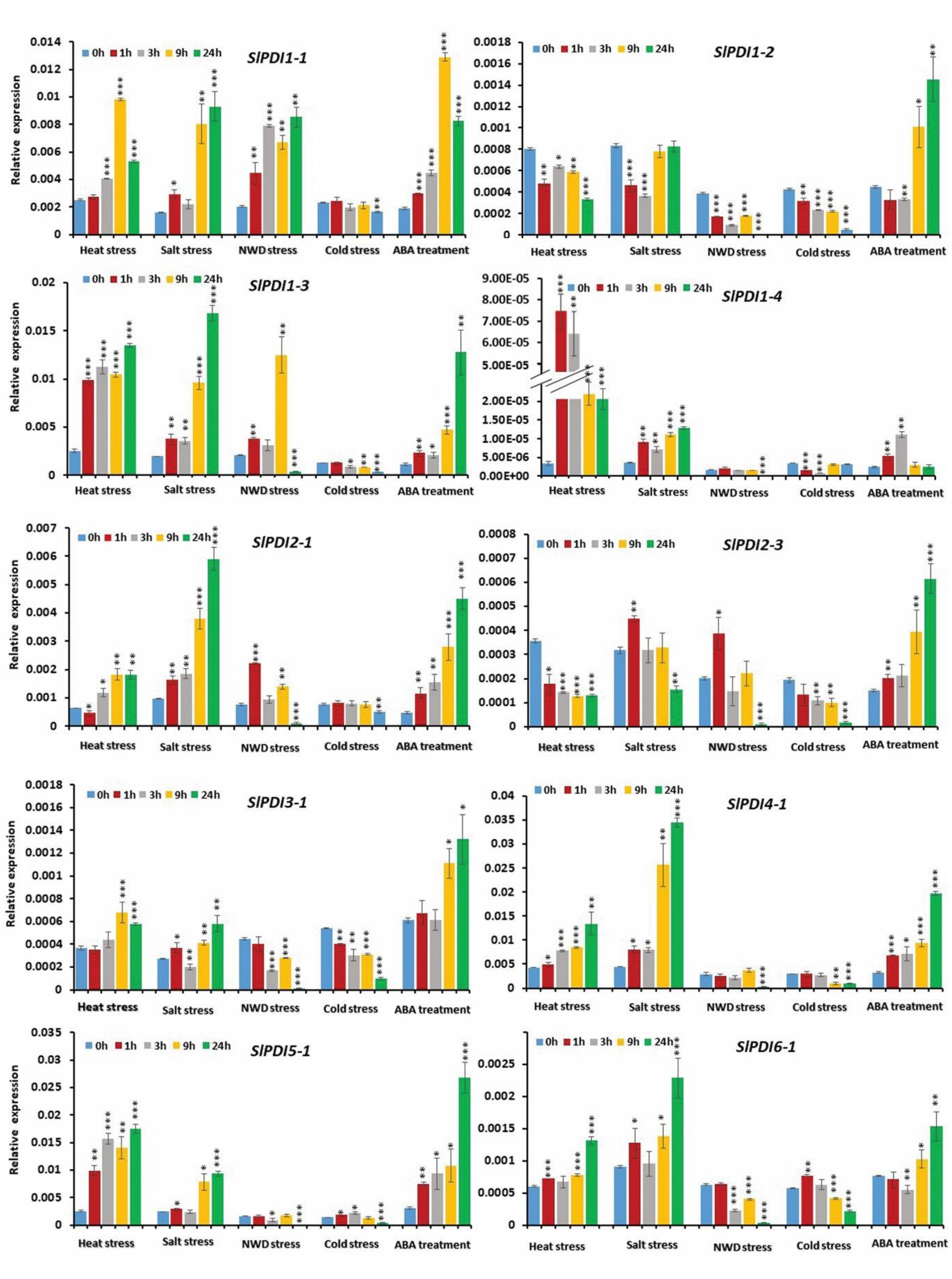

(a)

Figure S6. *Cont.*

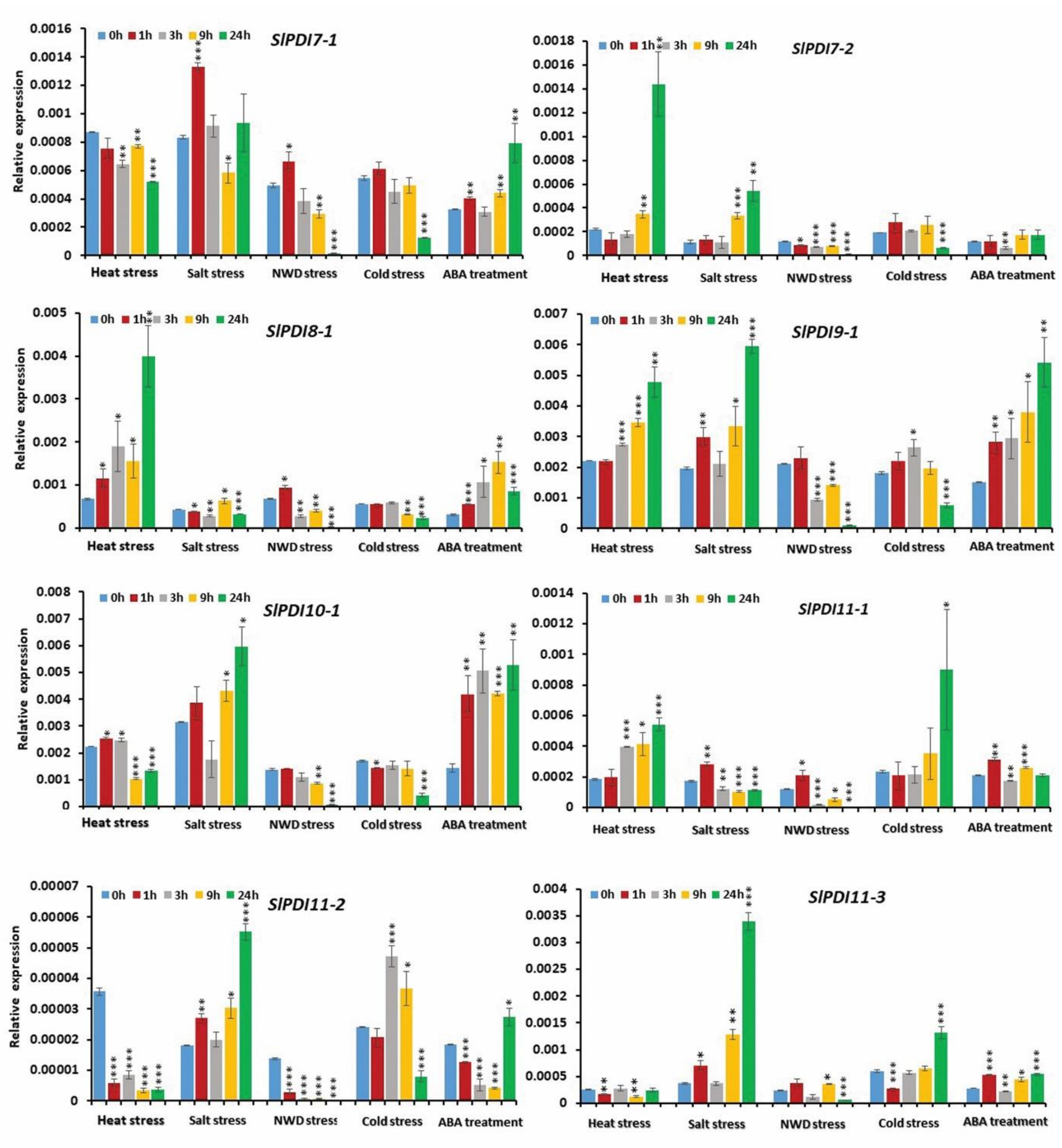

(b)

Figure S6 (a,b). Relative expression levels of *SlPDI* genes in response to various abiotic stresses, viz., heat stress, salt stress, nutrient and water deficit (NWD) stress and cold stress, and phytohormone treatment. Error bars indicate the standard errors of the means of three replicates. *, ** and *** represent the significant difference at P-value ≤ 0.05, ≤ 0.01 and ≤ 0.001, respectively.

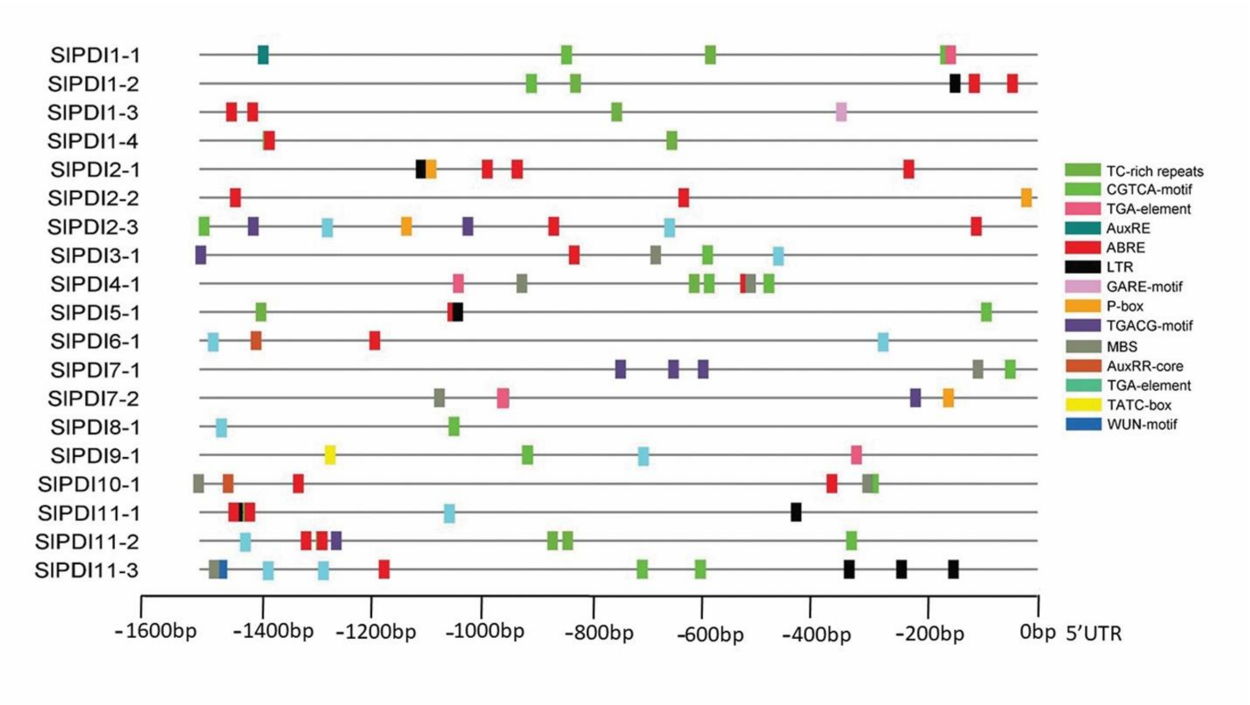

Figure S7. Predicted *cis*-regulatory elements in the promoters of tomato *PDI* genes. Elements shown are: a stress-responsive element (TC-rich repeats), jasmonic acid-responsive elements (TGACG-motif, CGTCA-motif), a salicylic acid-responsive element (TCA-element), auxin-responsive elements (AuxRE, TGA-element, AuxRR-core), a ABA-responsive element (ABRE), a low-temperature-responsive element (LTR), gibberellin-responsive elements (GARE-motif, P-box, TATC-box), a MYB binding site (MBS), and a wound-responsive element (WUN-motif). The scale indicates the position of each *cis*-regulatory element relative to the start codon (taken as +1 bp).

Table S1. Sequence identity among the 19 PDI proteins of tomato

	A	B	C	D	E	F	G	H	I	J	K	L	M	N	O	P	Q	R	S
SlPDI1-1(A)	100																		
SlPDI1-2(B)	51	100																	
SlPDI1-3(C)	62	49	100																
SlPDI1-4(D)	61	55	32	100															
SlPDI2-1(E)	32	28	31	30	100														
SlPDI2-2(F)	30	26	28	27	53	100													
SlPDI2-3(G)	33	29	31	32	62	66	100												
SlPDI3-1(H)	25	22	22	21	28	29	31	100											
SlPDI4-1(I)	22	18	22	23	21	21	24	19	100										
SlPDI5-1(J)	24	19	21	23	22	21	22	20	27	100									
SlPDI6-1(K)	23	19	21	22	23	25	24	18	29	24	100								
SlPDI7-1(L)	23	19	23	23	21	19	21	20	17	21	22	100							
SlPDI7-2(M)	22	17	21	21	20	18	19	19	17	17	26	69	100						
SlPDI8-1(N)	15	11	12	12	13	12	14	12	15	14	22	14	12	100					
SlPDI9-1(O)	16	13	13	13	15	13	14	10	16	13	19	14	13	13	100				
SlPDI10-1(P)	13	13	14	16	15	13	14	16	11	15	14	10	16	11	11	100			
SlPDI11-1(Q)	13	13	15	16	16	15	17	12	11	10	14	10	8	8	9	9	100		
SlPDI11-2(R)	11	13	14	14	15	14	17	12	9	9	11	9	8	8	8	7	**82**	100	
SlPDI11-3(S)	13	13	14	15	16	14	17	12	10	10	14	9	8	8	9	8	**80**	**82**	100

Table S2. Putative *cis*-elements of tomato *PDI* genes identified using PlantCARE database

Gene	Promoter	Consensus sequence	organism	Position	Strand	Function
SlPDI1-1	TC-rich repeats	ATTCTCTAAC	*Nicotiana tabacum*	576	+	cis-acting element involved in defense and stress responsiveness
	CGTCA-motif	CGTCA	*Hordeum vulgare*	154	-	cis-acting regulatory element involved in the MeJA-responsiveness
	CGTCA-motif	CGTCA	*Hordeum vulgare*	834	-	cis-acting regulatory element involved in the MeJA-responsiveness
	TGA-element	AACGAC	*Brassica oleracea*	145	-	auxin-responsive element
	AuxRE	TGTCTCAATAAG	*Glycine max*	1378	-	part of an auxin-responsive element
SlPDI1-2	ABRE	ACGTG	*Arabidopsis thaliana*	34	-	cis-acting element involved in the abscisic acid responsiveness
	CGTCA-motif	CGTCA	*Hordeum vulgare*	897	+	cis-acting regulatory element involved in the MeJA-responsiveness
	ABRE	CACGTG	*Arabidopsis thaliana*	102	+	cis-acting element involved in the abscisic acid responsiveness
	LTR	CCGAAA	*Hordeum vulgare*	137	+	cis-acting element involved in low-temperature responsiveness
	TC-rich repeats	ATTCTCTAAC	*Nicotiana tabacum*	818	+	cis-acting element involved in defense and stress responsiveness
SlPDI1-3	ABRE	ACGTG	*Arabidopsis thaliana*	1397	-	cis-acting element involved in the abscisic acid responsiveness
	ABRE	ACGTG	*Arabidopsis thaliana*	1435	-	cis-acting element involved in the abscisic acid responsiveness
	GARE-motif	TCTGTTG	*Brassica oleracea*	341	-	gibberellin-responsive element
	TC-rich repeats	GTTTTCTTAC	*Nicotiana tabacum*	744	-	cis-acting element involved in defense and stress responsiveness
SlPDI1-4	CGTCA-motif	CGTCA	*Hordeum vulgare*	1369	+	cis-acting regulatory element involved in the MeJA-responsiveness

	TC-rich repeats	ATTCTCTAAC	*Nicotiana tabacum*	645	+	cis-acting element involved in defense and stress responsiveness
	ABRE	ACGTG	*Arabidopsis thaliana*	1367	-	cis-acting element involved in the abscisic acid responsiveness
SlPDI2-1	ABRE	ACGTG	*Arabidopsis thaliana*	220	+	cis-acting element involved in the abscisic acid responsiveness
	ABRE	ACGTG	*Arabidopsis thaliana*	922	+	cis-acting element involved in the abscisic acid responsiveness
	ABRE	ACGTG	*Arabidopsis thaliana*	976	+	cis-acting element involved in the abscisic acid responsiveness
	LTR	CCGAAA	*Hordeum vulgare*	1095	+	cis-acting element involved in low-temperature responsiveness
	P-box	CCTTTTG	*Oryza sativa*	1077	+	gibberellin-responsive element
SlPDI2-2	ABRE	CACGTG	*Arabidopsis thaliana*	624	+	cis-acting element involved in the abscisic acid responsiveness
	ABRE	CACGTG	*Arabidopsis thaliana*	1428	-	cis-acting element involved in the abscisic acid responsiveness
	P-box	CCTTTTG	*Oryza sativa*	9	-	gibberellin-responsive element
SlPDI2-3	TGACG-motif	TGACG	*Hordeum vulgare*	1011	+	cis-acting regulatory element involved in the MeJA-responsiveness
	TGACG-motif	TGACG	*Hordeum vulgare*	1396	+	cis-acting regulatory element involved in the MeJA-responsiveness
	CGTCA-motif	CGTCA	*Hordeum vulgare*	1484	+	cis-acting regulatory element involved in the MeJA-responsiveness
	ABRE	ACGTG	*Arabidopsis thaliana*	857	+	cis-acting element involved in the abscisic acid responsiveness
	TCA-element	CCATCTTTTT	*Nicotiana tabacum*	652	+	cis-acting element involved in salicylic acid responsiveness
	ABRE	ACGTG	*Arabidopsis thaliana*	99	-	cis-acting element involved in the abscisic acid responsiveness
	P-box	CCTTTTG	*Oryza sativa*	1121	-	gibberellin-responsive element
	TCA-	CCATCTTTTT	*Nicotiana*	1266	-	cis-acting element involved in salicylic acid

	element		*tabacum*			responsiveness
SlPDI3-1	CGTCA-motif	CGTCA	*Hordeum vulgare*	581	+	cis-acting regulatory element involved in the MeJA-responsiveness
	TGACG-motif	TGACG	*Hordeum vulgare*	1490	+	cis-acting regulatory element involved in the MeJA-responsiveness
	ABRE	ACGTG	*Arabidopsis thaliana*	820	+	cis-acting element involved in the abscisic acid responsiveness
	MBS	CAACTG	*Arabidopsis thaliana*	674	+	MYB binding site involved in drought-inducibility
	TCA-element	TCAGAAGAGG	*Brassica oleracea*	457	+	cis-acting element involved in salicylic acid responsiveness
SlPDI4-1	CGTCA-motif	CGTCA	*Hordeum vulgare*	471	+	cis-acting regulatory element involved in the MeJA-responsiveness
	CGTCA-motif	CGTCA	*Hordeum vulgare*	578	+	cis-acting regulatory element involved in the MeJA-responsiveness
	MBS	CAACTG	*Arabidopsis thaliana*	914	+	MYB binding site involved in drought-inducibility
	TGA-element	AACGAC	*Brassica oleracea*	1028	+	auxin-responsive element
	TC-rich repeats	GTTTTCTTAC	*Nicotiana tabacum*	605	+	cis-acting element involved in defense and stress responsiveness
	ABRE	GCAACGTGTC	*Hordeum vulgare*	513	+	cis-acting element involved in the abscisic acid responsiveness
	MBS	CAACTG	*Arabidopsis thaliana*	504	-	MYB binding site involved in drought-inducibility
SlPDI5-1	CGTCA-motif	CGTCA	*Hordeum vulgare*	81	+	cis-acting regulatory element involved in the MeJA-responsiveness
	ABRE	ACGTG	*Arabidopsis thaliana*	1038	+	cis-acting element involved in the abscisic acid responsiveness
	TC-rich repeats	ATTCTCTAAC	*Nicotiana tabacum*	1382	+	cis-acting element involved in defense and stress responsiveness
	LTR	CCGAAA	*Hordeum*	1029	-	cis-acting element involved in low-temperature

			vulgare			responsiveness
SlPDI6-1	AuxRR-core	GGTCCAT	*Nicotiana tabacum*	1391	+	cis-acting regulatory element involved in auxin responsiveness
	TCA-element	CCATCTTTTT	*Nicotiana tabacum*	1471	+	cis-acting element involved in salicylic acid responsiveness
	ABRE	ACGTG	*Arabidopsis thaliana*	1178	-	cis-acting element involved in the abscisic acid responsiveness
	TCA-element	CCATCTTTTT	*Nicotiana tabacum*	269	-	cis-acting element involved in salicylic acid responsiveness
SlPDI7-1	CGTCA-motif	CGTCA	*Hordeum vulgare*	38	+	cis-acting regulatory element involved in the MeJA-responsiveness
	TGACG-motif	TGACG	*Hordeum vulgare*	589	+	cis-acting regulatory element involved in the MeJA-responsiveness
	TGACG-motif	TGACG	*Hordeum vulgare*	642	+	cis-acting regulatory element involved in the MeJA-responsiveness
	TGACG-motif	TGACG	*Hordeum vulgare*	737	+	cis-acting regulatory element involved in the MeJA-responsiveness
	MBS	CAACTG	*Arabidopsis thaliana*	96	+	MYB binding site involved in drought-inducibility
SlPDI7-2	TGACG-motif	TGACG	*Hordeum vulgare*	208	+	cis-acting regulatory element involved in the MeJA-responsiveness
	TGA-element	AACGAC	*Brassica oleracea*	946	+	auxin-responsive element
	MBS	CAACTG	*Arabidopsis thaliana*	1062	-	MYB binding site involved in drought-inducibility
	P-box	CCTTTTG	*Oryza sativa*	149	-	gibberellin-responsive element
SlPDI8-1	CGTCA-motif	CGTCA	*Hordeum vulgare*	1036	+	cis-acting regulatory element involved in the MeJA-responsiveness
	TCA-element	CCATCTTTTT	*Nicotiana tabacum*	1456	+	cis-acting element involved in salicylic acid responsiveness
SlPDI9-1	CGTCA-motif	CGTCA	*Hordeum vulgare*	904	+	cis-acting regulatory element involved in the MeJA-responsiveness

	TGA-element	AACGAC	*Brassica oleracea*	314	+	auxin-responsive element
	TATC-box	TATCCCA	*Oryza sativa*	1258	-	cis-acting element involved in gibberellin-responsiveness
	TCA-element	CCATCTTTTT	*Nicotiana tabacum*	699	-	cis-acting element involved in salicylic acid responsiveness
SlPDI10-1	ABRE	ACGTG	*Arabidopsis thaliana*	358	+	cis-acting element involved in the abscisic acid responsiveness
	ABRE	ACGTG	*Arabidopsis thaliana*	1315	+	cis-acting element involved in the abscisic acid responsiveness
	CGTCA-motif	CGTCA	*Hordeum vulgare*	284	+	cis-acting regulatory element involved in the MeJA-responsiveness
	MBS	CAACTG	*Arabidopsis thaliana*	1494	+	MYB binding site involved in drought-inducibility
	AuxRR-core	GGTCCAT	*Nicotiana tabacum*	1441	+	cis-acting regulatory element involved in auxin responsiveness
	MBS	CAACTG	*Arabidopsis thaliana*	294	-	MYB binding site involved in drought-inducibility
SlPDI11-1	CGTCA-motif	CGTCA	*Hordeum vulgare*	1404	+	cis-acting regulatory element involved in the MeJA-responsiveness
	LTR	CCGAAA	*Hordeum vulgare*	1424	+	cis-acting element involved in low-temperature responsiveness
	TCA-element	CCATCTTTTT	*Nicotiana tabacum*	1047	+	cis-acting element involved in salicylic acid responsiveness
	ABRE	ACGTG	*Arabidopsis thaliana*	1402	-	cis-acting element involved in the abscisic acid responsiveness
	ABRE	ACGTG	*Arabidopsis thaliana*	1431	-	cis-acting element involved in the abscisic acid responsiveness
	LTR	CCGAAA	*Hordeum vulgare*	422	-	cis-acting element involved in low-temperature responsiveness
SlPDI11-2	CGTCA-motif	CGTCA	*Hordeum vulgare*	323	+	cis-acting regulatory element involved in the MeJA-responsiveness

	CGTCA-motif	CGTCA	*Hordeum vulgare*	1274	+	cis-acting regulatory element involved in the MeJA-responsiveness
	TGACG-motif	TGACG	*Hordeum vulgare*	1247	+	cis-acting regulatory element involved in the MeJA-responsiveness
	TCA-element	CCATCTTTTT	*Nicotiana tabacum*	1413	+	cis-acting element involved in salicylic acid responsiveness
	TC-rich repeats	GTTTTCTTAC	*Nicotiana tabacum*	832	-	cis-acting element involved in defense and stress responsiveness
	TC-rich repeats	GTTTTCTTAC	*Nicotiana tabacum*	859	-	cis-acting element involved in defense and stress responsiveness
SlPDI11-3	CGTCA-motif	CGTCA	*Hordeum vulgare*	594	+	cis-acting regulatory element involved in the MeJA-responsiveness
	CGTCA-motif	CGTCA	*Hordeum vulgare*	698	+	cis-acting regulatory element involved in the MeJA-responsiveness
	LTR	CCGAAA	*Hordeum vulgare*	140	+	cis-acting element involved in low-temperature responsiveness
	LTR	CCGAAA	*Hordeum vulgare*	233	+	cis-acting element involved in low-temperature responsiveness
	LTR	CCGAAA	*Hordeum vulgare*	327	+	cis-acting element involved in low-temperature responsiveness
	TCA-element	CCATCTTTTT	*Nicotiana tabacum*	1372	+	cis-acting element involved in salicylic acid responsiveness
	WUN-motif	AAATTTCCT	*Brassica oleracea*	1452	+	wound-responsive element
	ABRE	ACGTG	*Arabidopsis thaliana*	1161	-	cis-acting element involved in the abscisic acid responsiveness
	MBS	CAACTG	*Arabidopsis thaliana*	1466	-	MYB binding site involved in drought-inducibility
	TCA-element	CCATCTTTTT	*Nicotiana tabacum*	1273	-	cis-acting element involved in salicylic acid responsiveness

Table S3. List of the PDI protein sequences used for phylogenetic analysis

Name	Accession	Sequence
SlPDI1-1	Solyc06g060290	MAIRVWISLFLCVFALLGSESYASENEDTQSKEFVVTLDHSNFSDFVGRHKFIVVEFYAPWCGHCK KLVPEYEKAAEILSQNDPPVVLAKVDANEEQNKALASEFDIKGFPTLKILRYGGSVVQDYKGPREA DGIVSYVKKQSGPASAEIKSSKDAEDFIDVNKIIIVGVFPEFSGEKFENFTAVAERLRADYDFGHTLD AKLLPRGDSSVSGPVVRLFKPFDELFVDFQVFDVDALAKLVEEATIPTVTVFNKDPNNHPFVVKFF NSPNAKAMLFVNFNIFDSFKSKYHEVAEQYKGNDISFLIGDVEASQGAFQYFGLKEDQTPLIIIQTNE GEKYLKTNVEPDHIASWVKEFKDGKVKPYKKSEPIPEVNNEPVKVVVADNFQDMVFNSGKNVLIE FYAPWCGHCKQLAPILDEVAVSFESDADVMIAKIVRCYC
SlPDI1-2	Solyc05g018700	MMKFLVLAALLAVTAFAAENEVVWTLDHTNYSQIISQHKMIVVQFYAPWCGYCQQLEPKYEEAA SVLSNHDPPVILAKMDTTILENAELARNFTINGVPSIKIFRNGGKTVHDYKGTRETEGIIAYLKKHGG PASLEIKTEKDAATFIDEKKIVVAGIFPQLSLEKIINFTTLAEDLRVDYDFVHTLDAKLLPRGGLVDK PTIRLLKPYDELYADFEDFQIEAMQNFIEETSVPIMAILDDNPENQQFVNHFLHSPGDKVFLFLNFST DLDAFKIKYYDLALSYKGKETNFLLGDAESGKKALQYFGLDTDQTPLIFVLTMGSTKYVQRHVQP DNLASWLKDCKDGKLKPYLKSQPIPEFNNETVKVVVAETLEDMVFNSGKDVLLEFYRLGCRYCEE FAPVLDEIAISFEKDPHVVIAKIDGTENDIPRDVFEVEGFPTLYLRSSTGSLSRFEGNKTKEAIIEFIQT NRSSPAFDFSISQTQTDQVKDELFSFAN
SlPDI1-3	Solyc06g005940	MAKSGILVIVSALVVLAVCGVFAEENEYVLTLDHSNLTETVAKHNFIVVEFYAPWCGHCKSLAPEY EKAASELSSHDPPIVLAKYDANDEANRELSKQYEIQGFPTIKILRDGGKKVQDYNGPREAAGIVSYL KKQVGPASAEIKSKEDATNLIDEKSIFVVGIFPDPSGEKFENYLTLAEKLRGEFDFAHTVDAKHLPR GGPVNKPTLRLLKPFDELFVDFEDFDVDAMEKFISESSIPVVTIFDNDPNNHPYVNKFFEGTNAKAL LFVNFSSEFDAFKSKYNDVAVIYKGDGVSFLLGDVEAGQGAFEYFGLKPEQAPVIIIMDADEQKYIK DHVEPDAIAAYLKDYKEGKLKPHVKSEPIPEVNDEPVKVVVRDTLQDMVYKSGKNVLLEFYAPW CGHCKSLAPILDEVAVSFESDPDVLIAKLDMTQPQMISRKVTLMFRDSLLCTSDPPLVTCHSTMVRE QKRLSSNSSRRIVASLLSQTLPKSIQQRMNFRGL
SlPDI1-4	Solyc05g056400	MKMEWSGVLVVLCVVFSVCVAEEEKEYVLTLDHTNFTDTITKYNFIVVEFYAPWCGHCKKLAPE YEKAASILSSHDPQIVLAKLDASDDASRELAIKYELQGFPTIKILRDGGKNVQEYKGPRDADGIVAY LKKQTGPASAEIKSKEDVASLIDEKKINVVGVFPELSGEKFDKFITLAEKLRADYAFAHTVDAKLLP RGEPVDKPTIRLLKPFDELFVDFEDFDVDAAEKFIAEATVPIVTIFDQEPENQVYVSKFFKTPNAKVL IFVNFSTEFDAFQSKYKDVAVSYKGDGLSFLLGDVEAGAGVFKYFGLEPEQAPVIIIMVNEGEKYIS THVQPDVLASWLKDYKDGKLKQFFKSEPIPEVNNEPVKVVVRDTLRDLVLNSGKNVLLEFYAPWC GHCKALAPILDEVALSFEKDSDVLIAKLDATANDIPKGEFDIKGFPTLYFKSASGNISQYEGDRTKE AIIEFIEKNRDKPAAHHSESSVKADSTKPESVTTDSAKDEFFGIICLHGGLKFCCGFAVLCLMFVILCI SCN

SlPDI2-1	Solyc04g049450	MAMKKYLILSIFLLNLIFFRTAFSSVVEEEEDLSFLEEEDSDAHSDSNPYGDHHDFENYEDLEDDSSD HGEDSYEPPAVDEKDVAVLKEGNFSEFISKNKYVMVEFYAPWCGHCQALAPEYAAAATELKGEN VMLAKVDATEEAELAQKYDVQGYPTVFFLIDGVHKTYNGERNKDAIISWIKKKTGPVLSNITTVEE AEQILKDEKKVVLGYLNDLVGDKSEELAAAAALEDDVNFYQTANPDVAKLFHIDSQAQRPALVII KKEAENINHFDGEFTKSAIAKFVFENKLPLVTNFTRESASEIFENPITKQLILFATSKDSEKFLPIFQEA VKAFKGKLICVFVEIDNEDVGKPVSEYFGVSGDAPRVLAYTGNEDGRKFILEGEITLDGVKSFGEKF LEDNLKPFYKSDPIPETNDGDVKIVVGNNFDEIVLDESKDVLLELYAPWCGHCQSLEPIYNKLGKH LRGIDSLVIAKMDGTTNEHPRAKSDGFPTLLFFPAGNKSFEPITVDTDRTVVAFYKFLKKHASTPFKI QKPVSTQRTTESDASLSHESTTNDAKDEL
SlPDI2-2	Solyc06g075210	KCNKIMPSRLIIFLCFSSLLFLTPSHAASSDDNDEFLSFLEDYEDSDEPDTPNHEDPPEVSSPPFDRAQ EAEDINIDDENVVALTDSNINDFLEDNKYVMLEFYTPWNGYCKALAPEYADAATELKTENVVLAK INAAKEVEAADSYDVRSFPTIFFFVNSDPELYKGRRTKNAIVSWIKKKIGSGVYNITTTEDAERVLTS EDKVVLAYLDSLVGSVTKQLAAVSKLENDVNFYQTTDPNVAKLFNIEENAKRPALVMLKKEPEKV VHYDGKFKKSSIAKFVSASKLPLVTTFTKETAPLIFASPIKKHILLFASENDTEKLFPTFQDAAKLFK GKMKLNFVFVKTNDEEVGRPVSDYFGVTGDGPQVIGYIEEDNRKKFRFNEDITLEKIKAFNEDFLE DKLKPFYKSDPIPETNDADVKIVVGNNFDEIILNESKDVFLEIYAPWCRQCQALEPMINKLAHHLHG VESLVIAKMEGTSNEHPRAKFIGFPSLQFFPAGNKSIDPILVDTEPTLVSLYKFIKKNAAIPFKIERPAS SKVGVKRENGNARDEL
SlPDI2-3	Solyc11g069400	MTSRLILLLFLSSLLIFSVLLTPSLAGTGADDDDDEDLSFLEADDVNDAAPEHTFPTETFSDDDDFDD DEDFENYDDFEVPSAFDDEKEEEGVKFDDKDVVVLTDRNFSDFVEDNKYVMVEFYAPWCGHCKE LAPEYAAAATELKSENVPLVKVDATVENELAESYEVQGFPTIYFFVDGEHKTYSGQRTKDAIVTWI KKKIGPGVYNITTTEDAERVLTSGDKVVLGFLESLVGPETDQLAAASKLEDDLNFYQTTNPNVAKL FNVEDTVKRPALVLLKKEEEKVVHYDGQFTKSAIVKFVSANKLPLVTVFTRESGASIFSSPIKKQVL LFARTNDTDKVFPTFQEAAKHFKGKLIFVFVNMDDEEVGKPVSDYFGATGDSPKVIGYTGNENPR KYIFDGEITVEKLKAFGEDFLADKLKAFYKSDPIPEDNEGDVKIVVGNNFDDIVLDESKDVLLEIYA PWCGHCQSLEPTYNKLAKHLRGIESLVIAKMDGTTNEHPQGKAEGFPTLLFFPAGNKTADPIPVDS DRTVVALYKFIKKHATIPFKLQKPASSTTTSESPEAKVGGNVESKNEKDEL
SlPDI3-1	Solyc03g120720	MFTPKPTSRFILFTLILLLLFSFLAPSISSELDLDDEEGDDMEGLEELMALDEEDDLQQKPQGGHHEK PSEAEFVSKAQRIVLELNNDNTKRAIDGNEYVLVLGYTPWCARSAELMPKFAEAATALKELGSPLL MAKIDAERYPKVASTLEIRGFPTLLLFVNGTSQPYTGGFSAEELVIWARKKTGVPIVRISSDAEARHF LKKHSIFVVGLFEKFEGPDYDAFTKAAEMDNEIQFVETNNAETAKHLYPDFKPTSLFLGLVKSEPEK YTEYEGIFSTDGILQFLDDNKFPLTTVLTELNAAKVYSNINKLQVLIIAETDDFKKLVEPLQDVARKF KSKIMFIFVDIREENLAKPFLSMVGLEESKDSVVVSFNYSSSLKYLLESDTTPTSIEEFCSGLLSGTVS PYYKSQPIPDNKNMSILTVVGKTFDELILNSPENILLEIYTPWCITCETTSKQMEKLAKHFKGLDNLIF ARIDASLNEHPKLQVDDYPTLLFYLADDKTNPIPLPTKSSTKELAALINKNLKEHSREIRDEL

SlPDI4-1	Solyc01g100320	MGRSQICSALAILALFFFSSALADDVVVLTEENFDKEIGQDRAALVEFYAPWCGHCKKLAPEYEKL GASFKKAKSVLIGKIDCDEHKSVCSKYGVQGYPTIQWFPKGSLEPKKYEGARSAEALAEYVNSEAG TNVKIASIPSSVVVLSSDNFDEIVLDETKDVLVEFYAPWCGHCKSLAPIYEKVATAFRQEEDVIIANL DADNHKDLAEKYGVSGYPTLKFFPKGNKAGEDYDGGRDADDFVNFINEKCGTSRDSKGQFTSKA GIVESLDTLVKEFVGATNEEKKAVFSKMEDEAGKLKGSAARYGKIYLKAAKSSMEKGADYANNEI QRLERMLAKSISPTKADEFTLKKNILATFA
SlPDI5-1	Solyc07g049450	MDRASVFPLFLIVLHFIATANALYGPTSPVVQLTESNFKSKVLNSKGIVLVEFFAPWCGHCQALTPA WEKAATILKGVATVAALDADAHKSLAQEYGIRGFPTIKVFAPGKPPVDYQGAREAKPIAEYALQQI KALLKERIHGKATGGSSESSEPSASVELNSRNFDENVLKSKDLWIVEFFAPWCGHCKKLAPEWKKA AKNLQGKVKLGHVDCDAEKSLMSRYNVQGFPTILVFGADKESPVTYEGARTASAIESFGLEQLETN VAPPEVVELTSPDVMEEKCNSAAICFVSFLPDILDSKAEGRNKYLEMLLAVAEKFKRNPYSFVWVG AGKQPDLEKHVGVGGYGYPAMVALNVKKGVYAPLKSAFQRQPIIDFVKEAGLGGKGNLPLAATP SIVKSEPWDGKDGEIIEEDEFSLEELMGDDTPNKDEL
SlPDI6-1	Solyc11g069690	MKRCMKPFSCISLCCIFLLSSIYIAEAEVITLTGDTFNDKISEKDTAWFVKFCVPWCKHCKNLGTLW DDLGKTMEREDEIEVGQVDCGTDKPVCNKVDIHSYPTFKLFYNGEEVAKYQGRRTERY
SlPDI7-1	Solyc06g065320	MNLTLMLQFPVLIIFLVDFYAPWCGHCKRLAPQLDKAASILADLKKPISIAKIDADKYKRVGSKYGI DGYPTLKIFMHGVPTEYYGPRKADLLVRFLKKFVAADVAILNSDSAISEFVEAAGTSFPIFIGFGLNE SAISHFAVKYKKRAWFSVAKDFSDKTMEFYDFDKVPALVARHPNYDEQSIFYGPFEENFVEDYIKQ SLLPLTLPITEETLRLLKDDERKVILTILEDETDDRSKKLLKLLKAAASANRDFVFVFVGFKQWQGF AESFDVSKKTKLPKMVVWDGDEEYFSVVGSESVEDEDQGPKLHILFKDTRRKYHTETY
SlPDI7-2	Solyc11g019920	MVPFISISIFTFGFLLLFRCSSSSAEQQQFAIDGKVLELDESNFEAAISTFDYMFVDFYAPWCGHCKR LSPELDKASANLAVLKQPIVIAKVDADKYSRLASKYEIDGFPTLKIFMHGVPTDYYGPRKADLLVR FLKKFVAPDVSVLNSDSAISEFIEEAGKNFPIFIGFGLNESVISHLAVKYKKSAWFSVAKDFSDTTME FYDFDKVPALVTLHLSYNEQSIFYGPFEEKFLEDYIKQSLLPLVLPINQDTLKSLKDDKRKIVLTIVE DEDDERSKRLVKLLKAAASANRDLVFAFVGFKQWQDFAESFEVSKKIKLPKMIVWDGDVEYFSVI GSDSVEDEDQGSQITRFLKGYRDGSVIQKHIISDDYKAFRNSMFLIGALILVLVVILVAMMMQAVK EEPSREQVDHPGSSTSLSEAREALRSGDKEEKID
SlPDI8-1	Solyc07g064250	MVSTSKIKSVDFYRKIPRDLTEASLSGAGLSIVAALCMMFLFGMELNNYLTVSTTTSVIVDKSSDAE FLRIDFNMSFPALSCEFASVDVSDVLGTNRLNITKTVRKHSIDKNLRPTGSEFHSGSTATELKHDAE DDEEYGEGSVSLNGHSFDRVTHHFPILVVNFFAPWCYWSNRLKPSWEKAANIIRERYDRESDGRIL VAKVDCTEEVDLCRRNHIQGYPSIRIFRKGTDVRDDHGHHDHESYYGDRDTDSLVKMMEDLVAPI KLDSQTITSDNSSTKLETGLKRPAPVTGGCRIEGFVRVKKVPGNLVISARSAAHSFDASQMNMSHVI SSFSFGKTITPKVMSDIKILLPHLGRSHDRLNGNSYVTNPRDSTENVTIEHFLQVVKTEVMTRSYKL VEEYEYTAHSSLVHSLHIPVAKFHYEPSPMQVLITENPKSFSHFLTNVCAIIGGVFTVAGILDSILHNT MRMVKKVELGKNF

SlPDI9-1	Solyc04g007610	MSCSIFLLIFALLVSILEQSVSVSVSNGSQRVILRAISGQDGEKPDFAVELNATNFDSVLKETPAPYAI<br>VEFFAHWCPACRNYKPQYEKVARLFNGADASHPGLILMTRVDCALKINSNLCDKFSVKYYPMLL<br>WGPPKKLVGWDPKQENNEILTIERGRTADILLGWINKQLGSSYGFDDGKYENEHLQRNFSDPGQIA<br>KAIYDIEEATSSAFGIILDQRMIKSGTRASLIKFLQLLVAHHPSKRCRKGSADILVDFDNLCPSEILLA<br>NNEADSCSKKGALGNYQICGKEVPRGYWMYCRGSKNDTRGFSCGLWVLLHSLSVRVEDGESDLA<br>FRTTCDFIYNFFVCEECRQHFHGMCSSVSSPFKKARDFALWLWSAHNQVNERLMKDEESLGTGDP<br>EFPKVTWPPKQLCPSCYLSPGKTSDKNSKIDWNENEVFKFLVSYYGKELVNLYKDKELQAGVGTE<br>KTVNEELVASTNAVVVPLGAALAIAVASCAFGALACFWRSQQKNRKPRRSWN
SlPDI10-1	Solyc04g074240	MGLLSAFAAGFVVILMVFEGMASPETANVSVRSVCPLKSVKDSIFMGFQDLTCPLDGIQSSYVAEV<br>IQGDELSLQRALSMIHRNTHDYVALLFYASWCPFSRSFRPKFSIMSSLYPSIPHFAIEESAVKPSTLSK<br>YGVHGFPTLFLLNSTMRMRYHGSRSLDSVIEFYGGTTGFHSASVDATSLGKMGCSSNLLKLHGSD<br>QENCPFSWARSPENLFQQETYLALATAFVLLKVLYMIFPALRKIAQIGLGGCILQIRIRSLWELPLLY<br>LNRAVQLCNSLKEPCKRSNLQEGAMNAKAWASKSLASVSFGESSASRVEPVSSTH
SlPDI11-1	Solyc03g031620	MALAFSSSAVIHGSLSSSSSYEQQQPKVSHMGITQPMDRSRIPLKAVNFTQRSLAMKPLVPLAATIV<br>APEVEQKAEADDYEKLAKELTNASPLKIMDKALEKFGSEIAIAFSGAEDVALLEYARLTGRPFRVFS<br>LDTGRLNPETYQLFDAVEKHYGIRIEYMFPDAVEVQALVRNKGLYSFYEDGHQECCRIRKVRPLRR<br>ALKGLRAWITGQRKDQSPGTRSEIPIVQIDPSFEGLDGGVGSLVKWNPVANVDGSDIWNFLRAMN<br>VPVNSLHSQGYVSIGCEPCTRAVLPGQHEREGRWWWEDSKAKECGLHKGNIKDESMNGNGNATV<br>HANGSAAHADIFDTNDIVSLSRPGIENLLKLENRREPWIVVLYAPWCRFCQAMEGSYVELAEKLAS<br>SGVKVAKFRGDGEQKTFAQEELQLGSFPTILFFPRQSSQPIKYPSEKRDVDSLLAFVNALR
SlPDI11-2	Solyc02g080640	MALTFTSSSAIHGSLSSSSSSYEQPKVSQLGTFQPLDRPQLLSSTVLNSRRRSAVKPLYAEPKRNDSI<br>VPSAATIVAPEVGESVEAEDFEKLAKELQNASPLEVMDKALEKFGDDIAIAFSGAEDVALIEYAHLT<br>GRPYRVFSLDTGRLNPETYQLFDTVEKHYGIRIEYMFPDSVEVQALVRTKGLFSFYEDGHQECCRV<br>RKVRPLRRALKGLRAWITGQRKDQSPGTRSEIPIVQVDPSFEGLDGGAGSLVKWNPVANVDGKDI<br>WNFLRAMNVPVNSLHSQGYVSIGCEPCTRPVLPGQHEREGRWWWEDAKAKECGLHKGNIKDETV<br>NGAAQTNGTATVADIFDTKDIVTLSKPGVENLVKLEDRREPWLVVLYAPWCQFCQAMEGSYVEL<br>AEKLAGSGVKVGKFRADGDQKAFAQEELQLGSFPTILFFPKHSSKAIKYPSEKRDVDSLLAFVNAL<br>R
SlPDI11-3	Solyc02g032860	MALAFTSSTAIHGSISSSSHQHPKVGNIQLLDQPKKSLNFTQRRCAVKPLYAEPKRNESIVPSAATFV<br>APEVAEKLIEAEDFEKLAKDLENASPLEIMDNALEKFGDDIAIAFSGAEDVALIEYAHLTGRPFRVFS<br>LDTGRLNPETYQLFDAVEKHYGIRIEYMFPDAVEVQALVRTKGLFSFYEDGHQECCRVRKVRPLR<br>RALKGLRAWITGQRKDQSPGTRSEVPVVQVDPSFEGLDGGSGSLVKWNPVANVEGKDIWNFLRA<br>MNVPVNSLHLKGYVSIGCEPCTRPVLPGQHEREGRWWWEDAKAKECGLHKGNIKAESVNGNGN<br>NATQTNDTVADIFDTKGIVTLSRPGVENLLKLEDGREPWLVVLYAPWCQFCQAMEGSYVELAEKL<br>GGSGVKVGKFRADGEQKTFAQQELQLGSFPTILFFPKHASQPIKYPSEKRDVNSLLAFVNALR

AtPDIL1-1	AT1G21750	MAMRGFTLFSILVLSLCASSIRSEETETKEFVLTLDHTNFTDTINKHDFIVVEFYAPWCGHCKQLAPE<br>YEKAASALSSNVPPVVLAKIDASEETNREFATQYEVQGFPTIKIFRNGGKAVQEYNGPREAEGIVTY<br>LKKQSGPASAEIKSADDASEVVSDKKVVVVGIFPKLSGSEFDSFMAIAEKLRSELDFAHTSDAKLLP<br>RGESSVTGPVVRLFKPFDEQFVDSKDFDGEALEKFVKESSIPLITVFDKDPNNHPYVIKFFESTNTKA<br>MLFINFTGEGAESLKSKYREVATSNKGQGLSFLLGDAENSQGAFQYFGLEESQVPLIIIQTADDKKY<br>LKTNVEVDQIESWVKDFKDGKIAPHKKSQPIPAENNEPVKVVVSDSLDDIVLNSGKNVLLEFYAPW<br>CGHCQKLAPILDEVAVSYQSDSSVVIAKLDATANDFPKDTFDVKGFPTIYFKSASGNVVVYEGDRT<br>KEDFISFVDKNKDTVGEPKKEEETTEEVKDEL
AtPDIL1-2	AT1G77510	MAFKGFACFSILLLLSLFVSSIRSEETKEFVLTLDHSNFTETISKHDFIVVEFYAPWCGHCQKLAPEY<br>EKAASELSSHNPPLALAKIDASEEANKEFANEYKIQGFPTLKILRNGGKSVQDYNGPREAEGIVTYL<br>KKQSGPASVEIKSADSATEVVGEKNVVAVGVFPKLSGDEFDSFMALAEKLRADYDFAHTLDAKFL<br>PRGESVEGPAVRLFKPFDELFVDSKDFNGEALEKFVKESSIPLVTVFDSDPNNHPYVAKFFESPATK<br>AMMFVNFTGATAEALKSKYREVATSNKDQSLAFLVGDAESSQGAFQYFGLEESQVPLIIIQTPDNK<br>KYLKVNVEVDQIESWFKDFQDGKVAVHKKSQPIPAENNEPVKVVVAESLDDIVFKSGKNVLIEFY<br>APWCGHCQKLAPILDEVALSFQNDPSVIIAKLDATANDIPSDTFDVKGFPTIYFRSASGNVVVYEGD<br>RTKEDFINFVEKNSEKKPTSHGEESTKSEEPKKTEETAAKDEL
AtPDIL2-1	AT3G54960	MASSSTSISLLLFVSFILLLVNSRAENASSGSDLDEELAFLAAEESKEQSHGGGSYHEEEHDHQHRDF<br>ENYDDLEQGGGEFHHGDHGYEEEPLPPVDEKDVAVLTKDNFTEFVGNNSFAMVEFYAPWCGACQ<br>ALTPEYAAAATELKGLAALAKIDATEEGDLAQKYEIQGFPTVFLFVDGEMRKTYEGERTKDGIVT<br>WLKKKASPSIHNITTKEEAERVLSAEPKLVFGFLNSLVGSESEELAAASRLEDDLSFYQTASPDIAKL<br>FEIETQVKRPALVLLKKEEEKLARFDGNFTKTAIAEFVSANKVPLVINFTREGASLIFESSVKNQLILF<br>AKANESEKHLPTLREVAKSFKGKFVFVYVQMDNEDYGEAVSGFFGVTGAAPKVLVYTGNEDMRK<br>FILDGELTVNNIKTLAEDFLADKLKPFYKSDPLPENNDGDVKVIVGNNFDEIVLDESKDVLLEIYAP<br>WCGHCQSFEPIYNKLGKYLKGIDSLVVAKMDGTSNEHPRAKADGFPTILFFPGGNKSFDPIAVDVD<br>RTVVELYKFLKKHASIPFKLEKPATPEPVISTMKSDEKIEGDSSKDEL
AtPDIL2-2	AT5G60640	MAFRVLLLFSLTALLIFSAVSPSFAASSSDDVDDEDLSFLEDLKEDDVPGADSLSSSTGFDEFEGGEE<br>EDPDMYNDDDDEEGDFSDLGNPDSDPLPTPEIDEKDVVVIKERNFTDVIENNQYVLVEFYAPWCG<br>HCQSLAPEYAAAATELKEDGVVLAKIDATEENELAQEYRVQGFPTLLFFVDGEHKPYTGGRTKETI<br>VTWVKKKIGPGVYNLTTLDDAEKVLTSGNKVVLGYLNSLVGVEHDQLNAASKAEDDVNFYQTV<br>NPDVAKMFHLDPESKRPALVLVKKEEEKISHFDGEFVKSALVSFVSANKLALVSVFTRETAPEIFES<br>AIKKQLLLFVTKNESEKVLTEFQEAAKSFKGKLIFVSVDLDNEDYGKPVAEYFGVSGNGPKLIGYT<br>GNEDPKKYFFDGEIQSDKIKIFGEDFLNDKLKPFYKSDPIPEKNDEDVKIVVGDNFDEIVLDDSKDV<br>LLEVYAPWCGHCQALEPMYNKLAKHLRSIDSLVITKMDGTTNEHPKAKAEGFPTILFFPAGNKTSE<br>PITVDTDRTVVAFYKFLRKHATIPFKLEKPASTESPKTAESTPKVETTETKESPDSTTKSSQSDSKDE<br>L

AtPDIL3-1	AT1G52260	MSLIPKPISKVSTFTFILLILLSFTIIIAYSSPDSNVESNEPGFDSDLDQLLAVDEQLQEDRPEQQSEAET<br>VSKAQRIVLELNGDYTKRVIDGNEFVMVLGYAPWCARSAELMPRFAEAATALKEIGSSVLMAKID<br>GDRYSKIASELEIKGFPTLLLFVNGTSLTYNGGSSAEDIVIWVQKKTGAPIITLNTVDEAPRFLDKYH<br>TFVLGLFEKFEGSEHNEFVKAAKSDDEIQFIETRDSDVAKLLFPDLKSNNVFIGLVKPEAERYTVYD<br>GSYKMEKILEFLGSNKFPLFTKLTETNTVWVYSSPVKLQVMLFSKADDFQKLAQPLEDIARKFKSK<br>LMFIYVDITNENLAMPFLILFGIEAGNKTVVAAFDNNLNSKYLLESDPSPNSIEEFCSGLAHGTVSRY<br>YRSEPVPDNENASIVTVVGKTFDGLVLNSRENVLLEVHTPWCVNCEALSKQIEKLAKHFKGFENLV<br>FARIDASANEHTKLQVDDKYPIILLYKSGEKEKPLKLSTKLSAKDIAVFINEELLKPKNGSAKDEL
AtPDIL3-2	AT3G16110	MLTKPKPNSKFSILFTFLLLLSFLIFVARSSDVAVEAGSEEELDDLEQLLAVDEQLQEERPEQQSEAE<br>TVSKAQRIVVELNGDNTKRLIDGNEYVMVLGYAPWCARSAELMPRFAEAATDLKEIGSSVLMAKI<br>DGERYSKVASQLEIKGFPTLLLFVNGTSQSYTGGFSSEEIVIWVQKKTGASTIKLDTVDEASGFLKK<br>HHTFILGLFEKSEDSSGHDEFVKAASLDNEIQFVETSSIDVAKLLFPNLKTNNVFVGLVKTEAEKYT<br>SYDGPCQAEKIVEFLNSNKFPLVTKLTESNTVRVYSSPVKLQVMVFSKTDDFESLAQPLEDIARKFK<br>SKLMLIYIDISNENLAMPFLTLFGIEDAKKTVVAAFDNNLNSKYLLESDPSPSNIEEFCFGLAHGTVS<br>AYYKSQPIPDNQNASVVAVVGRTFDEVVLRSSENVLLEVHTPWCINCEALSKQVEKLSQHFKGFEN<br>LVFARIDASANEHPKLTVDDYPTILLYKTGEKENPLKLSTKSSAKDMAVLINKELKWKDQSGKDEL
AtPDIL4-1	AT2G47470	MAKSQIWFGFALLALLLVSAVADDVVVLTDDSFEKEVGKDKGALVEFYAPWCGHCKKLAPEYEK<br>LGASFKKAKSVLIAKVDCDEQKSVCTKYGVSGYPTIQWFPKGSLEPQKYEGPRNAEALAEYVNKE<br>GGTNVKLAAVPQNVVVLTPDNFDEIVLDQNKDVLVEFYAPWCGHCKSLAPTYEKVATVFKQEEG<br>VVIANLDADAHKALGEKYGVSGFPTLKFFPKDNKAGHDYDGGRDLDDFVSFINEKSGTSRDSKGQ<br>LTSKAGIVESLDALVKELVAASEDEKKAVLSRIEEEASTLKGSTTRYGKLYLKLAKSYIEKGSDYAS<br>KETERLGRVLGKSISPVKADELTLKRNILTTFVASS
AtPDIL5-1	AT1G04980	MERKMYKSTVFPICCLLFALFDRGNALYGSSSPVLQLTPSNFKSKVLNSNGVVLVEFFAPWCGHCQ<br>SLTPTWEKVASTLKGIATVAAIDADAHKSVSQDYGVRGFPTIKVFVPGKPPIDYQGARDAKSISQFA<br>IKQIKALLKDRLDGKTSGTKNGGGSSEKKKSEPSASVELNSSNFDELVTESKELWIVEFFAPWCGHC<br>KKLAPEWKKAANNLKGKVKLGHVNCDAEQSIKSRFKVQGFPTILVFGSDKSSPVPYEGARSASAIE<br>SFALEQLESNAGPAEVTELTGPDVMEDKCGSAAICFVSFLPDILDSKAEGRNKYLEMLLSVADKFK<br>KDPYGFVWVAAGKQPDLEKRVGVGGYGYPAMVALNAKKGAYAPLKSGFEVKHLKDFVKEAAK<br>GGKGNLPIDGTMEIVKTEAWDGKDGEVVDADEFSLEDLMGNDDEASTESKDDL
AtPDIL5-2	AT2G32920	MYKSPLTLLTLLTICFGFFDLSSALYGSSSPVVQLTASNFKSKVLNSNGVVLVEFFAPWCGHCKALT<br>PTWEKVANILKGVATVAAIDADAHQSAAQDYGIKGFPTIKVFVPGKAPIDYQGARDAKSIANFAYK<br>QIKGLLSDRLEGKSKPTGGGSKEKKSEPSASVELNASNFDDLVIESNELWIVEFFAPWCGHCKKLAP<br>EWKRAAKNLQGKVKLGHVNCDVEQSIMSRFKVQGFPTILVFGPDKSSPYPYEGARSASAIESFASE<br>LVESSAGPVEVTELTGPDVMEKKCGSAAICFISFLPDILDSKAEGRNKYLEMLLSVAEKFKKQPYSF

		MWVAAVTQMDLEKRVNVGGYGYPAMVAMNVKKGVYAPLKSAFELQHLLEFVKDAGTGGKGN<br>VPMNGTPEIVKTKEWDGKDGELIEEDEFSLDELMGGDDAVGSKDEL
AtPDIL6-1	AT1G07960	MTLGARLVAPMIILLLFIPIELVKAEVITLTPETFSDKIKEKDTAWFVKFCVPWCKHCKKLGNLWED<br>LGKAMEGDDEIEVGEVDCGTSRAVCTKVEIHSYPTFMLFYNGEEVSKYKGKRDVESLKAFVVEET<br>EKAAEKAQLEDKEL
AtPDIL7-1	AT1G35620	MRSLKLLLCWISFLTLSISISASSDDQFTLDGTVLELTDSNFDSAISTFDCIFVDFYAPWCGHCKRLNP<br>ELDAAAPILAKLKQPIVIAKLNADKYSRLARKIEIDAFPTLMLYNHGVPMEYYGPRKADLLVRYLK<br>KFVAPDVAVLESDSTVKEFVEDAGTFFPVFIGFGLNESIISGLGRKYKKKAWFAVSKEVSEDTMVS<br>YDFDKAPALVANHPTYNEHSVFYGPFEDGFLEEFVKQSFLPLILPINHDTLKLLKDDERKIVLTIVED<br>ETHESLEKLYKALRAAAHANRDLVFGYVGVKQFEEFVDSFHVDKKTNLPKIVVWDGDEEYDQVT<br>GIETITQEEDHLTQVSRFLEGYREGRTEKKKINGPSFMGFINSMIGIRSVYILVFLVAVIMMLRSLGQ<br>VEEPTGVRTATAVRERVDQATTVPEDESSEHKPSDKKED
AtPDIL8-1	AT3G20560	MVSSTKLKSVDFYRKIPRDLTEASLSGAGLSIVAALFMMFLFGMELSSYLEVNTTTAVIVDKSSDG<br>DFLRIDFNISFPALSCEFASVDVSDVLGTNRLNITKTVRKFPIDPHLRSTGAEFHSGLALHNINHGEET<br>KEEFPDGAIPLTSASFEALSHHFPILVVNFNAPWCYWSNRLKPSWEKAANIIKQRYDPEADGRVLLG<br>NVDCTEEPALCKRNHIQGYPSIRIFRKGSDLREDHGHHEHESYYGDRDTDSIVKMVEGLVAPIHPET<br>HKVALDGKSNDTVKHLKKGPVTGGCRVEGYVRVKKVPGNLVISAHSGAHSFDSSQMNMSHVVS<br>HFSFGRMISPRLLTDMKRLLPYLGLSHDRLDGKAFINQHEFGANVTIEHYLQTVKTEVITRRSGQEH<br>SLIEEYEYTAHSSVAQTYYLPVAKFHFELSPMQILITENPKSFSHFITNLCAIIGGVFTVAGILDSIFHN<br>TVRLVKKVELGKNI
AtPDIL8-2	AT4G27080	MVSTSKIKSVDFYSDPEVVGFASRVCALSLSFDLSDIELFRCYFLGFKMIVSHFDFGIVAKRVASKKI<br>PRDLTEASLSGAGLSIIAALSMIFLFGMELNNYLAVSTSTSVIVDRSADGDFLRLDFNISFPSLSCEFA<br>SVDVSDVLGTNRLNVTKTIRKFSIDSNMRPTGSEFHAGEVLSLINHGDETGEEIVEDSVPLTGRNFD<br>TFTHQFPILVVNFYAPWCYWCNLLKPSWEKAAKQIKERYDPEMDGRVILAKVDCTQEGDLCRRN<br>HIQGYPSIRIFRKGSDLKDDNAHHDHESYYGDRDTESLVKMVVSLVEPIHLEPHNLALEDKSDNSSR<br>TLKKAPSTGGCRVEGYMRVKKVPGNLMVSARSGHSFDSSQMNMSHVVNHLSFGRRIMPQKFSE<br>FKRLSPYLGLSHDRLDGRSFINQRDLGPNVTIEHYLQIVKTEVVKSNGQALVEAYEYTAHSSVAHS<br>YYLPVAKFHFELSPMQVLITENSKSFSHFITNVCAIIGGVFTVAGILDSILHHSMTLMKKIELGKNF
AtPDIL8-3	AT1G50950	MVSTSKIKSVDFYRKIPRDLTEASLSGAGLSIVAALAMLFLFGMELSSYLAINTSTSVIVDKSSDGDF<br>LNIDFNISFPALSCEFASVDVSDVFGTHRLNISKTIRKVPIDPHLRATAEEFHSTSDLHLINHGDEDHG<br>DNSTYADIPLTGAAFEKFTHHFQILVVNFYAPWCYWSNRLKPSWVKASQITRERYNPGTDDRVLL<br>GSVDCTEEPTLCKSNHIQGYPSIRIFRRGSGLREDHGNHEHESYYGDRDTDSLVKMVEELLKPIKKE<br>DHKLALDGKSDNAASTFKKAPVSGGCRIEGYVRAKKVPGELVISAHSGAHSFDASQMNMSHIVTH<br>LTFGTMVSERLWTDMKRLLPYLGQSYDRLNGKSFINERQLDANVTIEHYLQIIKTEVISRRSGQEHS

		LIEEYEYTAHSSVARSYHYPEAKFHFELSPMQVLISENPKSFSHFITNVCAIIGGVFTVAGILDSIFQN<br>TVRMVKKIELGKNI
AtPDIL9-1	AT1G15020	MSLIHLFLLLGLLSLEAAASFSPGSRSILRDIGSNVADQKDNAIELNATNFDSVFQDSPAKYAVLEFF<br>AHWCPACRNYKPHYEKVARLFNGADAVYPGVVLMTRVDCAIKMNVKLCDKFSINHYPMLFWAP<br>PKRFVGGSWGPKQEKNEISVVNEWRTADLLLNWINKQIGSSYGLDDQKLGNLLSNISDQEQISQAIF<br>DIEEATEEAFDIILAHKAIKSSETSASFIRFLQLLVAHHPSRRCRTGSAEILVNFDDICPSGECSYDQES<br>GAKDSLRNFHICGKDVPRGYYRFCRGSKNETRGFSCGLWVLMHSLSVRIEDGESQFAFTAICDFIN<br>NFFMCDDCRRHFHDMCLSVKTPFKKARDIALWLWSTHNKVNERLKKDEDSLGTGDPKFPKMIWP<br>PKQLCPSCYLSSTEKNIDWDHDQVYKFLKKYYGQKLVSVYKKNGESVSKEEVIAAAEEMAVPTNA<br>LVVPVGAALAIALASCAFGALACYWRTQQKNRKYNYNPHYLKRYNSNYMVMNTFSNTESEREKE<br>R
AtPDIL9-2	AT2G01270	MSLVHLLLFAGLVIAASSSSPGSRLILREISDQKDKAVELNTTNFDSVLKDTPAKYAVVEFFAHWCP<br>ACRNYKPHYEKVARLFNGPDAIHPGIVLMTRVDCAMKTNTKLCDKFSVSHYPMLFWGPPTKFVSG<br>SWEPKKDKSEILVIDDGRTAERLLNWINKQIGSSYGLDDQKFKNEHALSNLTDYNQISQAVYDVEE<br>ATAEAFDIILAHKAIKSSETSASFIRFIQLLAAHHLSRRCRKGAAEILVNYDDLCPSGNCSYEKSGGN<br>DTLGNFPICGKDVPRGYYMFCRGSKNDTRGFSCGLWVLMHSLSVRIEDGESHFAFTTICDFVNNFF<br>MCDECRLHFNDMCLSVKTPFKKARDFVLWVWSTHNKVNERLLKDEASLGTGDPKFPKIIWPPKEL<br>CPLCYLSSNQKSIEWDHEHVYKFLKNYYGPKLVSLYKEKSVSRSKEETVSATEDLTVATNALVVPI<br>GAALAIAIASCAFGALACYWRTQQKNRKPRRR
AtPDIL10-1	AT3G03860	MDSRVSILFVCAIAVSCFTSGSASSPVDFSVCNYEFELFRFDLEAKCPPSLYPTPPIEVDGDSLDRLM<br>ASQHGNAYMSVLFYASWCPFSRAVRPKFDMLSSMFPQIQHLAVEHSQALPSVFSRYGIHSLPSILM<br>VNQTLNARYHGRKDLISLIEFYEEATGLQPVQYVAEGEPTGLNAGDGNLITWLRKGTSIREIFKQDP<br>FLVLSLLFICLQMAILVFPIAESRMRALWASYVANLNLGRFGEISQLFNRGIHMVDVRRLWLKLSLV<br>KTRNFHERAKNAQAWASSLASVSLGQTSSDQS
AtPDIL10-2	AT1G34780	MEKEILLLLLVIMFLTVADVDAVRVPFCATKSAKDSIFGLRDQTCSVSGVESDERPRFVAVTEGDER<br>WLQIALDMIHKNKCDYVALLFYASWCPFSRSFRPSFDVISSLYSSIPHFAIKESSIKPSTLSKYGVHGF<br>PTLLLLNSTMRARYRGTRMLDSLVAFYSDVTGIETLDKTSLERSVSVPHLGNENNTEPENCPFTWA<br>RSPENMLRQETYLALAIVFVLLRLLHLIYPTLVVFMKFTWRRIAQNMRLESLLEHTVGFLSRAVQL<br>CMHRRSNLQGGAMNARAWASKSLATVSIGDSSSSNRRSSSSQ
AtPDIL11-1	AT4G21990	MALAINVSSSSSSAISSSSFPSSDLKVTKIGSLRLLNRTNVSAASLSLSGKRSSVKALNVQSITKESIV<br>ASEVTEKLDVVEVEDFEELAKRLENASPLEIMDKALEKFGNDIAIAFSGAEDVALIEYAHLTGRPYR<br>VFSLDTGRLNPETYRLFDTVEKHYGIRIEYMFPDAVEVQALVRNKGLFSFYEDGHQECCRIRKVRP<br>LRRALKGLRAWITGQRKDQSPGTRSEIPVVQVDPVFEGLDGGVGSLVKWNPVANVEGNDVWNFL<br>RTMDVPVNTLHAAGYVSIGCEPCTRAVLPGQHEREGRWWWEDAKAKECGLHKGNIKENTNGNA

		TANVNGTASVADIFNSENVVNLSRQGIENLMKLENRKEAWIVVLYAPWCPFCQAMEASFDELADK<br>LGGSGVKVAKFRADGDQKDFAKKELQLGSFPTILVFPKNSSRPIKYPSEKRDVDSLTSFLNLVR
AtPDIL11-2	AT1G62180	MALAVTSSSTAISGSSFSRSGASSESKALQICSIRLSDRTHLSQRRYSMKPLNAESHSRSESWVTRAS<br>TLIAPEVEEKGGEVEDFEQLAKKLEDASPLEIMDKALERFGDQIAIAFSGAEDVALIEYARLTGKPFR<br>VFSLDTGRLNPETYRLFDAVEKQYGIRIEYMFPDAVEVQALVRNKGLFSFYEDGHQECCRVRKVRP<br>LRRALKGLKAWITGQRKDQSPGTRSEIPIVQVDPVFEGLDGGVGSLVKWNPLANVEGADVWNFLR<br>TMDVPVNALHAQGYVSIGCEPCTRPVLPGQHEREGRWWWEDAKAKECGLHKGNIKEEDGAADS<br>KPAAVQEIFESNNVVALSKGGVENLLKLENRKEAWLVVLYAPWCPFCQAMEASYIELAEKLAGK<br>GVKVAKFRADGEQKEFAKQELQLGSFPTILLFPKRAPRAIKYPSEHRDVDSLMSFVNLLR
AtPDIL11-3	AT4G04610	MAMSVNVSSSSSSGIINSRFGVSLEPKVSQIGSLRLLDRVHVAPVSLNLSGKRSSSVKPLNAEPKTK<br>DSMIPLAATMVAEIAEEVEVVEIEDFEELAKKLENASPLEIMDKALEKYGNDIAIAFSGAEDVALIE<br>YAHLTGRPFRVFSLDTGRLNPETYRFFDAVEKHYGIRIEYMFPDSVEVQGLVRSKGLFSFYEDGHQ<br>ECCRVRKVRPLRRALKGLKAWITGQRKDQSPGTRSEIPVVQVDPVFEGLDGGVGSLVKWNPVAN<br>VEGNDVWNFLRTMDVPVNTLHAAGYISIGCEPCTKAVLPGQHEREGRWWWEDAKAKECGLHKG<br>NVKENSDDAKVNGESKSAVADIFKSENLVTLSRQGIENLMKLENRKEPWIVVLYAPWCPFCQAME<br>ASYDELADKLAGSGIKVAKFRADGDQKEFAKQELQLGSFPTILVFPKNSSRPIKYPSEKRDVESLTS<br>FLNLVR
BrPDI1-1	Bra016405	MAMRGYALFSILALSLLASSVRSEETATETTKEFVLTLDHTNFTDTVNKHDFIVVEFYAPWCGHCK<br>QLAPEYEKAASELSSHVPPVVLAKIDASEETNREFATQYEVQGFPTIKIFRNGGKAVQEYNGPREAD<br>GIVTYLKKQSGPASFEIKAAEDASEFDKKVIVVGVFPKLSGSEFDSFLATAEKLRSDYDFAHTSDAK<br>LLPRGESVTGPVVRLFKPFDELFVDSKDFDGEALEKFVKESSIPLITVFDKDPNNHPYVIKFFDSSNT<br>KAMLFINFTGEGAESLKSKYREVATSYKGQGLSFLLGDAENSQGAFQYFGLEESQVPLIIIQTVDDK<br>KYLKTNIEIDQIESWVKDFKDGKVAPHKKSQPIPTENNEPVKVVVAESLDEMVFNSGKNVLLEFYA<br>PWCGHCQKLVPILDEVAVSYQSDPSVVIAKLDATANDFPNDTFDVKGFPTIYLRSASGNIVLYDGD<br>RTKEDIISFIDKNKDTAGEPKKEETTTEAVKDEL
BrPDI1-2	Bra012293	MAAMRRGYALFSILALSLLASSVRSETKEFVLTLDHSNFTDTINKHDFIVVEFYAPWCGHCKQLAP<br>EYEKAASELSSNVPAVVLAKIDASEETNKEFATKYEVQGFPTIKIFRNGGKAVQEYKGPREADGIVS<br>YLKKQSGPASFEIKSGDDVVGDKKVVVVGVFPKLAGSEFDSFLATAEKLRSDYDFAHTSDAKLLPR<br>GESVTGPVVRLFKPFDELFVDSKDFDGEALEKFVKESSIPLITVFDKDPNNHPYVIKFFDSPNTKAMF<br>FINFTGESAETLKSKYREVATSNKGQGLSFLLGDAENSQGAFQYFGLEESQVPLIIIQTADDKKYLK<br>TNVEVDQIGSWIKDFKDGKVSPHKKSQPIPTENNEPVKVVVGESLDDMVFNSGKNVLLEFYAPWC<br>GHCQKLVPILVEVAVSYQSDPSVVIAKLDATANDFPRDTFDVKGFPTIYFRSASGNVVLYEGDRTK<br>EDFISFIDKNKDTAGEPKTEDKTAEATKDEL
BrPDI1-3	Bra017948	MAMKGYTLCSILVFSLFASCVRSKETKEFVLTLDHTNFTETINKHDFIVVEFYAPWCGHCKQLAPE<br>YEKAASELSSHVPPVVLAKIDASEETNKEFATKYSVQGFPTIKILRNGGKAVQEYNGPREADGIVTY

		LKKQSGPASLEIKSADAASEVVGDKNVVAVGVFPKLSGAEFDSFMATAEKLRSDYDFAHTTDAKL LPRGESVTGPVVRLFKPFDELFVDFRDFVGEALEKFVKESSIPLITVFDSDPNNHPYVLKFFEIPNTK ALFFLNFNGEGAETLKSKYREVAASNKGHGLSFLLGDAKNSEEALQHYGVEQRQLPLIILQTVDDK KYLKTNVEVDQIESWINDFKDGKASPYKKSQPIPGENNEPVKVVVAENLDEMVFSSGKNVLLEFY APWCGHCQNLVPILDEVAVSYQSDPSVVIAKFDATANDFPHDTFDVKGFPTIYLRSANGNIVLYKG DRTKEDIISFIDKNKDTAGETKTEEKKTKEVKDEL
BrPDI1-4	Bra008311	MAFKGFALFSIVVLSIFASSRSEETETKEFVLTLDHSNFTETINKHDFIVVEFYAPWCGHCKSLAPEY EKAASELITHNPPLVLAKIDASEESNKGIANEYKIQGFPTIKILRNGGKSIQDYNGPREAPGIVSYVKK QSGPASSEIKTAADAAEVVGEKNVVAVGVFPKLSGEEFDSFIALAEKLRGDYDFAHTLDAKLLPRG DSSVAGPVVRLFKPFDELFVDSKDFNGEALEKFLKESSIPLVTVFDSDPSNRPYVASFFDSSATKVM MFVNFTGESAESLKSKFRKVATSYKGQDLSFLVGDAEGGKGALEYFGVEESQVPLVIIQTPDSKKY LKANVVVEEIESWMKDFKDGKVDVFKKSQPIPAENNEPVKVVVAETLDDIVLKSGKNVLIEFYAP WCGHCQKIAPILDEVALAFKNDPSVIIAKLDATANDIPSEPFDVKGFPTIYFRSVSGTVVAYEGNRT KEDFISFIEKNKPTTSHVEDTTSSTKTEEPKKIDDASDTKDEL
BrPDI1-5	Bra015665	MASNGFAMLSILVLALFASSIRSEETETKEFVLTLDHSNFTDTINKHDFIVVEFYAPWCGHCKSLAPE YEKAAAELSSQSPPIFLAKIDASEESNKGIANDYKIQGFPTIKILRKGGKSIQDYNGPREAAGIVTYV KKQSGPASAEIKSADGAGEVIGEKSVVAVGVFPKLSGEEFDSFMALAEKLRADYDFAHTLDAKLLP RGDSSVAGPVVRLFKPFDELFVDSKDFNGEALEKFVKESSIPLVTVFDKDPSNHPYVSKFFDNPATK VMMFVNFTGETAESLKSKFREVATSSKGQDLAFLVGDAESSQGALQYFGLEESQVPLIIIQTPDSKK YLKANVVVDQIESWMKDFKDGKVAAHKKSQPIPAENNEPVKVVVAESLDEMVFNSGKNVLIEFY APWCGHCQKLAPILDEVALAFQNDPSVIVAKLDATANDIPSDTFDVKGFPTIYFRSADGKVVVYEG SRTKEDFISFIEKNKPASHSEESSTTVRSGEHKTEESAAKDEL
BrPDI2-1	Bra007120	MASSTSMSLLFLLSFLLLATSRAENAANGSDLDEELAFLAAEESKEEQQHHANSHHDQYRDFENYE DLEQGGEFHHGEHEGGGEYHEEEPQLPIVDEKDVAVLTKDNFTEFVGNNSFAMVEFYAPWCGAC QALAPEYAAAATELKGVAALAKIDATEEGDLAQKYEIQGFPTVFLFVDGEMRKTYEGERTKDGIV TWMKKKASPSIHNITTVEEAERVLSAEPKVVLAFLDSLVGSESAELAAASRLEDDLSFYQTTSPDIA KLFEIETEVKRPALVLLKKEEEKLARFDGNFTKAAISEFVSANKSPLVINFTREGASLIFENSVKNQLI LFATTNESEKHLPTLREVAKSFKGKFVFVYVQMDNEDYGEAVSGFFGVTGTAPKVLVYTGNEDM RKFILDGELTVNNIKTLAEDFLADKLKPFYKSDPVPETNDGDVKIIVGNNFDEIVLDESKDVLLEIYA PWCGYCQSFEPIYNKLGKYLKGIDSLVVAKMDGTTNEHPRAKADGFPTILFFPGGNKSFDPITVDV DRTVVELYKFLKKHASVPFKLAKPSATPEQVITTKKADEKTESDGAKDEL
BrPDI2-2	Bra002464	MAFRVFLLLSLTALLIFSAVSPSFSTSDVDDEDLSFLEDPKEEHDPTKPLTSTESELDEFNEGEEEDPE MYEGDDEEEGEDLSDLGNPDSDPFPTPDVDEKDVVVVKERNFTDVIENNQYVMVEFYAPWCGHC QSLAPEYAAAATELKGDGVVLAKIDATEENELAHQYSVQGFPTILFFVDGEHKPYTGGRTKDTIVT WVKKKIGPSVYNLTTLDDAEKVLTSGNKVVLGYLNSLVGVEHDQLAAASKAEDDVNFYQTVNPD

		VAKLFHIDPEAKRPAVVLVKREAEKISHFDGEFVKSDLASFVSANKLPLVSVFTRESAPEIFESAIKK<br>QILLFVTQNGSEKVLPEFEEAAKSFKGKLIFVSVDLDNEDYGKPVAEYFGVSGNGPKLIAYTGNEDP<br>KKHFFDGEIKSDKIKTFAEEFLSDKLKPFYKSDPIPEKNDGDVKIVVGDNFDDIVLDESKDVLLEVY<br>APWCGHCQALEPMYNKLAKHLREIDSLVIAKMDGTTNEHPKAKAEGFPTILFFPAGNKTAEPITVD<br>TDRTVVAFYKFLRKHATIPFKLEKPAASTESPKTAKSTPKVETTETKGNPQSTTKSTESDLKDEL
BrPDI2-3	Bra020239	MASRVFLLLSLTALLIFSAVSPSLSADVDDEEDLSFLEDLTEEVKAPAKPLTDDFEGGEDDDDEEED<br>GEHFSDVSNQDSDPFPLSDVDEKDVVVVKERNFTDVIENNEYVMVEFYAPWRGHCQSLAPEYAA<br>AATELKGDGVVLAKIDATVENELAHQYSVQGFPTILFFVDGEHKLYTGGRTKETIVTWVKKKIGPS<br>VYNLTTLDDAEKVLTSGNKVVLGYLNSLVGVEHDQLAAASKAEDDVNFYQTVNPDVAKMFHIDP<br>ESKRPALVLVKREEEKISHFDGEFVKSGLVSFVSANKLPLVTVFTPESSQEIFESAIKKQLLLFATEN<br>GSEKVLQEFEEAATLFKGKLIFVSVDVDNEDYGKPVAEYFGVSSSNAPKLVAFTGNEDPQKHYFEG<br>EIKSDKIKIFGEEFLSDKLKPFYKSDPIPEKNDGDVKIVVGDNFDEIVLDESKDVLLEVYAPWCGHC<br>QALEPMYNKLAKHLRSIDSVVIAKMDGTTNEHPKAKAEGFPTVLFFPAGNKTSSEPITVDADRTVV<br>AFYKFLRKHATIPFKLEKPAASTESPTAAESTPKVETTETKGKLESTTTKSTESDSKDEL
BrPDI3-1	Bra014319	MSMNPKLSVSTFILLLLLTFLLIPSHSSSSDEESDDDLEQLLAVDEQSQEDRPQHQQSEAETVSKAQR<br>IVLELTGDNAKRVVDGNEFVLVLGYAPWCARSADLMPRFSEAATGLKEIGSSVLMAKIDGDRYGK<br>VASELEIKGFPTLLLFVNGTSQPYSGGFSAEDIVIWVQKKTGSPIITVNTLDEAQIFLNKYHTFVVGL<br>FHKFEGSEYNEFVKAAKSDNEIQFVETSDNDVAKLLFPQLKTNTVFIGLVKPEAERYTAYDGPFKM<br>EKLLEFLGNNKFPLITRLTESNTVWVYSSPVKLQVMLFSKAYVFQSLAQPLEDLARKFKSKLMFIY<br>VDIANENLAMPFLTLFGIEHANKTVDFCSGLADGTVSRYYRSEPVPDNVDDFPTILLYKSGEKEKPV<br>ITFLIEQSYKPSM
BrPDI3-2	Bra018958	MSINPKPQSSLLTFILLLLLLTSAAYSSSNHPGSDEESDDLEQLLAVDEQLQQDLPLHHQQSEAETVSR<br>AQRIVLELSGDNARRVVGGNEFVMVLGYAPWCARSADLMPKFSEAATALKEIGSPVVMAKIDGD<br>RYGKVASEMEIKGFPTLLLFVNGTSKAYTGGFSAEEIVIWVQKKTGAPIVTVNTVDEAQRFLKKYH<br>TFVVGLFNKFEGSEYNEFVKAAKSDDEIQFVETSDSEVAKLLFPEIKTSDVFIGMVKTEAERYTSYA<br>GSYKMENILEFLSKNKFPLITKLSESNTAWVYSSPVKLQVMIFAKADDFQNMAQPLENFARRFKSK<br>LMFIYIDITNENLAMPFLTLFGIEHANKTVVAAFDNKLNSKYLLESDPSPTNIEDFCSGLADGTIPQY<br>YRSEPVPDNENASIVTVVGKTFDELVLNSQENVLLEVHTPWCVNCEAMSKQVVKLAKHFKGFENL<br>VFARIDASTNEHAKLQVNDYPTILLYKSGEKEKPLKISTKLSAKDMAVFINEELKPRGGSAKDEL
BrPDI4-1	Bra000454	MAKSQIWFGLASLVALLVVSAVADDVVVLTDDSFEKEVGKDRGALVEFYAPWCGHCKKLAPEYE<br>KLGASFKKAKSILIAKVDCDEHKSVCTKYGVSGYPTIQWFPKGSLEPQKYEGARNAEALAEYVNK<br>EGGTNVKLAAAPQNVVVLTPDNFDEIVLDQNKDVLVEFYAPWCGHCKSLAPVYEKVATVFKQEE<br>GVVIANLDADAHKSLGEKYGVSGFPTLKFFPKDNKAGQDYEGGRDLDDFVGFINEKVGTSRDSQG<br>QLTSKAGIVESLDALVKELVAASEDEKKTILSRIEEEASNLKGSTTRYGKLYSKLAKSYIEKGSAYA<br>TKEVERLGRVLGKSISPVKADELTLKKNILSTFVASS

BrPDI4-2	Bra004455	MAKSQIWFGLALVALLVVSAVADDVVVLTEDSFEKEVGKDKGALVEFYAPWCGHCKKLAPEYEK LAASFKKAKSVLIAKVDCDEHKGVCTKYDVSGYPTIKWFPKGSLEPQKYEGPRNAEALAEFVNKE GGTNVKLAAVPQNVVVLTPDNFDEIVLDQNKDVLVEFYAPWCGHCKSLAPVYEKVATVFKQEDG VVIANLDADAHKSLGEKYGVSGFPTLKFFPKDNKAGQDYDGGRDLDDFVTFINEKVGTSRDSKGQ LTSKAGVVESLDALVKELVAASEDEKKAILSRIEEEASNLKGSTARYGKLYSSLAKKYIEKGSGYA TKEAERLGRVLSKSMSPVKADELTLKRNILNTFVASS
BrPDI5-1	Bra015375	MNKTRVFTILSLVFAFSFDLSNALYGSSSPVLQLTPSNFKSKVINSNGVVLVEFFAPWCGHCKSLTP TWEKVATTLKGIATVAAIDADAHKSVSQDYGVRGFPTIKVFVPGKPPIDYQGARDAKAISQFAIKQI KALLKDRLDGKTTGTTTGGGSSEKKSEPSASVELNSSNFDELVTESKDLWIVEFFAPWCGHCKKLA PEWKKAAKNLKGKVKLGHVDCDADKAIQSRFKVKGFPTILVFGADKSSPLPYEGARSASAIESFAL EQLEANAGPAEVTELTGPDAMEEKCGPAAICFVSFLPDILDSKAEGRNKYLEMLLSVAEKFKKDPIS FVWVAAGKQPDLEKRVGVGGYGYPAMVALNAKKGAYAPLKSGFEVKHLIEFVKEAQKGGKGNL PIDGTLEIVKTEAWDGKDGEVVDADEFSLEELMADD
BrPDI5-2	Bra005546	MQNKSPLTLLTLLCLSLGFLNLTNALYGSSSPVVQLTASNFKSKVLNSNGVVLVEFFAPWCGHCKA LTPTWEKVASVLKGVATVAAIDADAHQSAAQDYGIQGFPTIKVFVPGKPPVDYQGARDAKSIANF AYKQIKALLSDRLEGKSKPSGGGSSEKKSEPSASVELNSSNFDELVIKSNDLWIVEFFAPWCGHCKK LAPEWKRAAKNLKGKVKLGHVNCDVEQSIMSRFKVQGFPTIMVFGVDKSSPYAYDGARSASAIES FATELVEASAGPVEVTELTGPDVMEKKCGSAAICFVSFLPDILDSKAEGRNKYLEMLLSVAEKFKR HPYSFVWVAAVTQPDLEKRVNVGGYGYPAMVAMNVKKGVYAPLKSAFELQHLLEFVKDAGAG GKGNVPMNGTPEIVETKAWDGKDGEVMEEDEFSLEELMGGDDDANVGTKDEL
BrPDI6-1	Bra018672	MKLGARLIAFILLLSLTIVLTKAEVITLTPETFSDKVKEKDTAWFVKFCVPWCKHCKKLGNLWEEL GNAMEGDDEIEIGEVDCGKSRDVCTKVEIHSYPTFKLFYNGEEVSKYQGKRDVESLKTFVVEETEK AAEKAQLEDKEL
BrPDI7-1	Bra034408	MRSLGLMYWWISFLALSISLSASSDDQFTIDGTVLELTDSNFESAISTFDCVFVDFYAPWCGHCKRL NPELDAAAPILAKLKQPIIIAKLNADKYSRLARKLEIDAFPTLMLYNHGVPMEYYGPRKADLLVRY LKKFVAPDVAVLESNSHVKDFVEDSGTSFPVFIGFGLNQSLISGLGRKYKKKAWFAVAKDASEDV MVSYDFDKAPALVAQHPAYNEHSVFYGPFEDGFLEEFVKQNFLPLILPINHDTLKLLKDDERKMVL TIVEDETHESMGKLIKALRAAAHANRDLVFGYVGVEQFEEFADSFHADKKAKLPKIVVWDGDEEY EQVNGIETVSHEEDHLTQVSRFLEGYREGKTEKKRIKGPSFMGFINSMIGIRSVYIIVFLVAVIMMLR SLGQVEEPARVRTAASDGQATSVLEGETSEHKPRDKED
BrPDI8-1	Bra001793	MVSPTKLKSVDFYRKIPRDLTEASLSGAGLSIVAALVMMLLFGMELSSYLEVTTTTAVVVDKSSDG DFLRIDFNISFPALSCEFASLDVNDVLGTNRLNITKTVRKFPIDPHLKATGGEFHSGLASHHINHGEEI KQEFPDGAIQLTNGGFQSLSHHFPLLIVNFNAPWCYWSNRLKPSWEKAATIIKQRYNPDTDGRVLL GSVDCTEEPALCRRNHIQGYPSIRIFRKGNDLKEDHGHHEHESYYGDRDTESIVKMVDELVAPIHPE THKLALDWGISNDTAKLLKKAPVTGGCRVEGYVRVKKVPGNLVISAHSGAHSFDSSQMNMSHVV

		THLSFGRMIDTRLLTDLKRLLPYLGQSHDKLDEKAFINQHEFGANVTIEHYLQIVKTEVITRRYGQEHSLTEEHEYTAHSSITQTYYLPVAKFHFELSPMQILITENPKSFSHFITNLCAIIGGVFTVAGIIDSVLHNTIRLIKKVELGKNI
BrPDI8-2	Bra035770	MVSPTKLKSMDFYRKIPRDLTEASLSGAGLSIVAALVMMLLFGMELSSYLAVNTTTAVVVDKSADGDFLRIHFNISFPALSCEFASVDVSDVLGTNRLNITKTIRKFPIDPHLKTTGEEFHSGHGSHDINHGEETKEEIPDGSVPLVSSSFDSFSKHFPLLIVNFNAPWCYWSNRLKPSWEKASSIIYHKYNPETDGRVLLGSVDCTEEAELCKRNHIQGYPSIRIFRKGSDLKEDHGHHEHESYYGDRDTDSIVKMVDELVAPIHPETHKLDLDGISNKTLKHLKKAPVTGGCRVEGYVRVKKVPGNLIISAHSGAHSFDSSKMNMSHVVSHLSFGRMFSPRLLTDMRRLLPYIGQSHDKLNEKAFINQHEFGANVTIEHYLQVVKTEVITRRTAQEHSLVEEYEYTAHSSIAQTYYLPVAKFHFELSPMQIMITENPKSFSHFITNLCAIIGGVFTVAGILDSIFHNTIRLVKKVELGKNF
BrPDI8-3	Bra019071	MVSTSRIKSVDFYRKIPRDLTEATLSGAGLSIVAALSMLFLFGMELNNYLAVSTTTSIIVDRSSDGDFLRMDFNISFPSLSCEFASVDVSDVLGTNRLNVTKTIRKFSIDSNLRPTGSEFHSGEVLSHVNHDEAGEEVVEDSVSLTSRNFDTLLHQFPISVVNFYAPWCYWCNLLKPSWEKAAKQIKERYDPEMDGRVILAKVDCTQEADLCRKNHIQGYPSIRIFRQGSDLKDNAHHDHESYYGDRDTESLVKMVIGLVEPIHLEPHKLALEDKSDNASKTLKKAPSTGGCRIEGYIRVKKVPGNLMVSARSGSHSFDSTQMNMSHVVNHLSFGRKILPQTFTDLKRLSPYLGQSHDRLNGRPFINQRDLGPNVTIEHYLQIVKTEVLKSNGHAMVEEYEYTAHSSVAHSYYLPVAKFHFELSPMQVLITENSRSFSHFITNVCAIIGGVFTVAGILDSILHQTMTLMKKIELGKNF
BrPDI8-4	Bra010413	MISPRKIKSVDFYRKIPRDLTEASLSGAGLSIVAALSMLLLFGMELSSYLTVSTTTSIIIDRSSDGDFLRMDFNISFPSVSCEFASVDVSDVLGTNRLNVTKTIRKFSIDSNLRPTGSEFHSGEFLSRVNHGDESAEELVEGSVSLGARNFDTFLHQYPISVVNFYAPWCYWCNLLKPSWEKAANQIKERYDPEMDGRVILAKVDCTQEADLCRRNHIQGYPSIRIFRKGSDLRDDNAHHDHESYYGDRDTESLVKMVIGLVEPIHLEPHKLALEDKSGNASKTLKKAPSTGGCRIEGYMRVKKVPGNLMVSARSESHSFDTSQMNMSHVVNHLSFGKRILPEAFSDLKRLAPYLGGSHNRLDDRSFINQHDLGPNVTIEHYLQIVKTEVLKSNGHAMIEEYEYTAHSSVAHTYYLPVAKFHFELSAMQVLITENSKSFSHFITNVCAIIGGVFTVAGILDSILHQTMTLMKKIELGKNF
BrPDI8-5	Bra030465	MVSTTKIKSVDFYRKIPRDLTEASLSGAGLSIIAALAMVFLFGMELSTYLAVTTNTSVIVDNSSDGDFLRIDFNVSFPSLSCEFASVDVSNVLGTKRLNLTKTIKKVPIDPYLRATGAEVHSTSGLHLINHGDEDHGNNTYAAIPLTGATFDKFSHHFQILVVNFYAPWCYWSNRLKPSWEKAAEITRQRYNPETDGRVLLGSVDCTEETTLCKRNHIQGYPSIRIFRKGSDLKEDHGHHEHESYHGDRDTESILKMVEELLKPIKKEDHKLALDGKTDNVVSGIKKAPVSGGCRIVGYVRAKKVPGEIIISAHSGAHSFDASQMNMSHYVSHLTFGKMISERLLTDMKRLMPYLGLSHDRLNSKWFVNEGQFAANVTIEHYLQVVKTEVVSRRFGQEHSVIEEYEYTAHSSVAHGYYYPVAKFRFDLSPMQVLISENPKSFSHFITNVCAIIGGVFTVAGILDSIFQNTFRLVKKIELGKNI

BrPDI8-6	Bra018881	MVSTTKIKSVDFYRKIPRDLTEASLSGAGLSIIAALAMMFLFGMELSTYLAVTTQTSVVVDNSSDDD<br>FLQIDFNVSFPALSCEFATFEVSDVLSTNRLNLTKTIKKVPIDPHLRDTGEEYHPTPDSDLINHGDEH<br>HDDNTYAAIPLSGGTFDKISHKFPILVVNFYAPWCYWSSRLRPSWEKAAEITRQKYGPENDGRVLL<br>GSVDCTEEPTLCTKYHIQGYPSIRIFHNGSDLRGDDGHQEHDSYHGNRDTESLVKMVEELLRPIKKF<br>DGTTNHAASRIRKAPVSGGCRIEGYVRAKKVPGELVISAVSGHSFDASRMNMTHFVNHLSFGRLI<br>SDRLLTDMKRLLPYLGLSHDRLNGKWFVNEGKFAANVTIEHYLQVVKTEVVSRRFGQEHSVIEEY<br>EYTAHSSVAHGYYYPVAKFHFDLSPMQVLISENPKSFSHFITNVCAIIGGVFTVAGILDSIFQSTYGI<br>MKKVELGKNF
BrPDI9-1	Bra026786	MSLIHLFLLVSLVSLEADATTSFSSGSRSILRDIGSNVIADHKDNAVELNATNFDSVFQDTSAKFAVL<br>EFFAHWCPACRNYKPHYEKVARLFNGPEAVHPGTVLMTRVDCAAKMNIKLCDKFSIKRYPMLFW<br>GPPSKFVGGSWEPKQEKSEILVVEEWRTADLLLGWINKQLGSSYGLDDQKVGNDHLLPNISDHEQI<br>SQAVFDIEEATEEAFDIILSLKAIKSSETGASFIRFLQLLVPHHPSKRCRKGSAEILMNFDDLCPAGEC<br>SYDSGVNNTLRNFHICGKDLPHGYYMFCRGSKNETRGFSCGLWILMHSLSVRIEDGESQFAFTTLC<br>DFINNFFMCDECRRHFHDMCLSVKTPFKKARDVVLWLWSTHNKVNERLKKDEDSLGTGDPKFPKI<br>IWPPKQLCPSCYLTSTGENIDWDHDEVYKFLKRYYGEKLVSSYKKNTGGVSKEEVVVAAAEEMSV<br>PRNALVVPVGAALAIALASCAFGALACYWRTQQKNRKHHHNPHYLRRYSSNYLVMNTFSNIESER<br>EKER
BrPDI9-2	Bra014330	MSLAHLVLFAGLLSLVILASSSSSSSSSPGSRSILRDISGENADQKDRAVELNSSNFDSVLSDTPAKY<br>AVVEFFAHWCPACRNYKPHYEKVARLFNGPDAIHPGIVLMTRVDCAMKTNTNLCDRFSVSHYPM<br>LLWGSPTKFVSGSGEPKKEKSEIVVIDDARTAERLLKWINKQTQSSYGLDDKKFENEHVRTNITDY<br>KQISQAVYDIEEATAEAFDIILSNKVIKSSETSASFIRFIQLLAAHHASRRCRKGAAEILVNYDDLCPS<br>GKCSYEASGGKDTLGSFPICGKDLPRGYYMFCRGSKNDTRGFSCGLWVLFHSLSVRIEDGESQFAF<br>NTICDFVNNFFMCDECRLHFNDMCLSVKTPFKKARDFVLWVWSTHNKVNERLMKDEASLGSGDP<br>EFPKIIWPPRALCPSCYLSSDEKSIEWDHDNVYKFLKSYYGPKLVSLYKEKSVVGSKEETVSATAED<br>LTVATNALVVPVGAALAIAVASCAFGALACYWRTQQKNRKYCRGEAGTEVLEAELLMFP
BrPDI10-1	Bra001092	MDLHLPILLLCVIAASCFPSGLASSPVDSSVCNHEFELFRFDLASKCPPSLRPSPPIEVDGDSLDRLMA<br>LNHDDGNAYVSVLFYASWCPFSRAVRTKFDMLSLMFPQIQHLAVEHSQALPSVFSRYGIHSLPSIL<br>MVNQTLKARYHGRKDLTSLIEFYDESTGLKPVQYVAEGEPATTLDATDGSLITWLRNGTSISEIFKR<br>DPFLVLSLLFICIQVAILVFPIAESRMKALWASYAPNLNLERFGEVSQVFSRALHMVDVRRLWLKLR<br>LVKTRSFHERAKNAQAWASSLASVSLGQTSSDQS
BrPDI10-2	Bra031969	MDLRVPILFFLLSTIYFPSVLASSSPVDFSVCNHEFELFRFDLDSKCPPSLHPAPPLQVDGETLDRLM<br>GLNYDANGYMSVLFYASWCPFSRAVRPKFDMLSSMFPLIQHLAVEHSQALPSVFSRYGIHSLPSILI<br>VNRTSKARYHGQKDLTSLIEFYEESTGLKPVQYVAEAEPTTSLDSTDGNLITWLRKGTSISEIFRQDP<br>FLVLSLLFICLQMAILVFPIAESRMKALWASYVSNLNLERFGEISQLFSRALHMVDVRRLWLKLRLV<br>KTRNFHERAKNAQAWASSLASVSLGQASSDQS

BrPDI10-3	Bra036758	MDLRVPFLLLIAVSCFPSSFASSSPSVCNHESELFRFDIHSKCPPSMYPTPPIEIDGDSLDRLMALHHD GNAYVSVLFYASWCPFSRALRSKFDTLSSMFPQIHHLALDHSQALPSVFSRYGIHSLPSILMVNQTS KARYHGRKDLTSLIEFYEESTGLKPVQYVSEPEPTTSVDATDGNMITWLRKGTSISEVFKGDPFLVL SLLFVCLQAAILVFPMAEQRLKALWASYVPNLNLERFGEVSQVFRRAVHMVDVRRLWLKLTLVK TRSFHERAKNARAWASSLASVSLGQTSSNQS
BrPDI10-4	Bra036429	MEKGILLLVLVILFGNLMFTAVDGVSVRAPICAMRSVKDYALGFREQSCPFYGDELADRPHFVVVT EGDERWLQTALDMIHKNKCDYVALLFYASWCPFSRSFTPSFDLISSLYSSIPHFAIKESSVKPSTLSK YGVHGFPTLLLMNSTMRARYRGTRMLDSLVAFYRDVTVSELTRKLLGIETLDKTSLEKSLLVPHLG NENNTEPENCPFTWARSPENMLRQETYLTLATVFVLLRLLYFVFPALVVFAKFTWPRIAQNMRLES LQEHTVGFLSRLCMYLKEPCKRSNLQGGAMNARAWASKSLATVSIGESSSSNSRASSASQ
BrPDI11-1	Bra019406	MALAINVSSSSSSISTSSFPSSDLKAPQIGSLRLSDRINVSSASLSLSGKRSSVKALNVQSITKESMVPP QAASMVASEIREKVDVIEVEDFEELAKKLETASPLEIMDKALENFGNDIAIAFSGAEDVALIEYAHL TGRPYRVFSLDTGRLNPETYRLFDTVEKHYGIRIEYMFPDAVEVQALVRSKGLFSFYEDGHQECCRI RKVRPLRRALKGLRAWITGQRKDQSPGTRSEIPVVQVDPVFEGLDGGAGSLVKWNPVANVEGND VWSFLRTMDVPVNTLHAAGYVSIGCEPCTRAVLPGQHEREGRWWWEDAKAKECGLHKGNIKESS NGNNAAVNGNGTTSTVDDIFKSENVVSLSRQGIENLMKLENRKEAWIVVLYAPWCPFCQAMEASF DELADKLRGGDGVKVAKFRADGDQKEFAKSELQLGSFPTILVFPKNSSRPIKYPSEKRDVDSLTSFL NLVR
BrPDI11-2	Bra013579	MALAINVSSSSSSAISTSSFPSSELKAPRIGSLRLSDRVNVSTASLSLSGKRSSSVKPLNVQSIAKESFV PSQAASVVASEVTEKLDVVEVEDFEELAKSLETASPLEIMDKALEKFGNDIAIAFSGAEDVALIEYA HLTGRPFRVFSLDTGRLNPETYRLFDTVEKHYGIRIEYMFPDAVEVQALVRNKGLFSFYEDGHQEC CRIRKVRPLRRALKGLRAWITGQRKDQSPGTRSEIPVVQVDPVFEGLDGGAGSLVKWNPVANVEG NDVWNFLRTMDVPVNTLHAAGYVSIGCEPCTRAVLPGQHEREGRWWWEDAKAKECGLHKGNIK ENSNGNANANVNGTSSTVADIFKSENVVSLSRQGIENLMKLENRKEAWIVVLYAPWCPFCQAMEG SFDELADKLGGSGVKVAKFRADGDQKEFAKRELQLGSFPTILVFPKNSSRPIKYPSEKRDVDSLTSF LNLVR
BrPDI11-3	Bra034466	MALAVTSSSTAISGSSFSRSGPCSDRKALQICSFRLSDLSHVSQRRYSLKAESPPTRNDSLVTRASTLI TPGVEEKEEDVEDFEQLAKKLEEASPLEIMDKALQRFGSNIAIAFSGAEDVALIEYARLTGRPFRVFS LDTGRLNPETYRLFDAVEKQYGIRIEYTFPDAVEVQALVRNKGLFSFYEDGHQECCRVRKVRPLRR ALKGLKAWITGQRKDQSPGTRSEIPIVQVDPVFEGLDGGVGSLVKWNPLANVEGGDVWNFLRTM DVPVNALHAQGYVSIGCEPCTRPVLPGQHEREGRWWWEDAKAKECGLHKGNIKKEDDSTTADLA PAIVHDIFESSNVVALSRGGIENLLKLGNRKEPWLVVLYAPWCPFCQAMEASYVELAEKLAVKGIK VAKFRADGDQKEFAKQELQLGSFPTILLFPKSAPRAIKYPSEHRDVDSLMSFVNLLRSVRKHDDKE LVLELMATLTRGRESY

BrPDI11-4	Bra029505	MAMAATVSSSGITSSAFSPSVISSEPKVSQIGSLKLLDRVSLTTPMSLKKRSSVKPLNAEPKRNDSMVPLAATMVAQVAEEVVETEDFAELAEKLENASPLEIMDKALEMFGNDIAIAFSGAEDVALIEYAHLTGRPYRVFSLDTGRLNPETYRFFDAVEKHYGIRIEYMFPDSVEVQGLVRSKGLFSFYEDGHQECCRVRKVRPLRRALKGLRGWITGQRKDQSPGTRSEIPVVQVDPVFEGLDGGAGSLVKWNPVANVEGSDVWSFLRTMDVPVNTLHAAGYVSIGCEPCTRAVLPGQHEREGRWWWEDAKAKECGLHKGNIKENANVNGESKPVVEDIFKSENVVALSRQGIENLVKLENRKEPWIVVLYAPWCPFCQAMEASYDEMADKLAGSGVKVAKFRADGEQKEFAKQELQLGSFPTILVFPKNSSRPIKYPSEKRDVDSLTSFLNLVR
PtPDIL1-1	POPTR_002G082100v3	MASTVSFWSCIFLLSLIVALSAGEDESKEYVLTLDHSNFNETVSKHDFIVVEFYAPWCGHCKKLAPEYEKAASILSSNDPQVVLAKVDANEDANKEIASQYDVKGFPTIVILRKGGKSVQEYKGPREADGIVEYLKKQSGPASAELKSDDDATGFIGDKKVVIVGVFPKFSGEEFENFLAVAEKLRSDYEFGHTLDAKYLPRGESSVSGPLVRLFKPFDELFVDSKDFNVDALEKFVEESSIPIVTLFNKDPSNHPFVVKYFDSPLAKAMLFMNFSSENGDSIRTKYQEVAGLHKGDGLVFLLGDVEASQGALQYFGLKEDQVPLIVIQTTDGQKYLKPNLVSDQIAPWLKEYKEGKVPPFKKSEPIPEVNDEPVKVVVADSLDELVTKSGKNVFLEFYAPWCGHCQKLAPILEEVAISFQSDADVVIAKLDATANDIPSDTYDVKGFPTIFFRSATGKLVQYEGDRTKQDIIDFIEKNRDKIGQQEPAKEEEPAKEQETAKDEL
PtPDIL1-2	POPTR_005G179000v3	MMASKVSLWSCIFVFSLVVALSTGEDESKEYVLTLDHSNFTETVTKHDFVVVEFYAPWCGHCQNLAPEYEKAASILSSNDPQIVLAKVNADEKVNQEISEKYEVQGFPTIKILRKGGTSVNEYKGPRDADGIAEYLKKQTGPASAELKSADDATSFIGDNKVVIVGVFPKFSGEEFESFLAVADKLRSDYEFAHTLDAKHLPRGESSVSGPLVRLFKPFDELFVDSKDFNVDALEKFIEESSAPIVTVYDDEPSNHPYIVKYFDSPLDKAMLFLNFSGDSADSIKTNYQEVAEQHKGDGLIFLLGDLEASQSALQYFGLKEDQAPLLVIQTTDGKKYLKSNLESDHIAPWVKEYKEGKVPPFIKSEPIPEANEEPVKVVVADSLDDLVTKSGKNVLLEFYAPWCGHCQKLAPILEEIAVSYQSDADVLLAKLDATANDIPGDTYDVKGFPTVYFRSASGKLVQYEGDKTKQDIIDFIEKNRDKVAQQEPAKDEL
PtPDIL2-1	POPTR_009G013600v3	MSTRFIFLLSLTALLLFSHLSPSLSKLQNAAAEDDDEDLSFLEEETDAVPHGQGHGHDHDHDHYPDPDQFDEEFDNEDDLDNYSDLDDSELDSYKEPEIDDKDVVVLKEGNFSDFVTKNKFVMVEFYAPWCGHCQSLAPEYAAAATELKAEEVMLAKVDATEENELAQEYDIQGFPTVYFFVDGVHRPYPGPRNKDGIVTWIKKKIGPGIYNITTVDDAERLLTSETKLVLGFLNSLVGPESEELAAASRLEDEVSFYQTVNPDVAKLFHLDPQAKRPALVMLKKEAEKLSVFDGNFSKSEIAEFVFANKLPLVTIFTRESAPLIFESTIKKQLLLFAISNDSEKVVPIFQEAARLFKGKLIFVYVEMDNEDVGKPVSEYFGISGTAPKVLAYTGNDDAKKFVFDGDVTLDKIKAFGEDFIEDKLKPFFKSDPVPESNDGDVKIVVGNNFDEIVLDESKDVLLEIYAPWCGHCQSLEPTYNKLATHLRGIESIVIAKMDGTTNEHPRAKSDGFPTLLFFPAGNKSFDPITVDTDRTVVAFYKFIKKHASIPFKLQKPASASKAESSDAKDGIESSTRDVKDEL
PtPDIL3-1	POPTR_001G183500v3	MFPAKPTSRSMLFTFTILLLLSSTIFVTANEDPTVETDNDGADSDLQELIAIDEQEGGGGEEQQQGDQQKEAEVLSKAQRIVLELNSDNARRVIDQNEFVLILGYAPWCARSAELMPQFAEAANKLKELGSPVLMAKLDAERYPKVASTLGIKGFPTLLLFVNGTSQVYTGGFSGEDIVIWARKKTGPVPVIRISSSVEAE

		DFQKKYHLFVLGLFDKFEGHDYEEFIKAATIDNEIQFVEVSSSAVAKILFPNINAKDNFIGIVKSEPEK<br>YTAYGGIFEKDTILQFLEYNKFPLVTILTELNSARVYSSPVKLQVIVFADADDFKNLIRPLQEVARKF<br>ISKIMFIYIDIADENQAKPFLTLFGIEDSENTVVTAFDNRMSSKYLLESNPTSSNIEEFCSRLLHGSLSP<br>YFKSQPIPDNKEKILQVVVGKTLDDLVLSSPKNVLLEVYTPWCISCETTTKQIEKLAKHFKGVDNLV<br>FARIDASANEHPKLLVDDYPTLLFYPVGDKENPVKLSTKSSSKDLATVIKSLLRAKEDVPKDEL
PtPDIL4-1	POPTR_002G198300v3	MERCNQIWYAFGTMALLAVSALADDVVVLTEDNFEKEVGQDKGALVEFYAPWCGHCKKLAPEY<br>EKLGSSFKKAKAVLIGKVDCDEHKGVCSKYGVSGYPTLQWFPKGSLEPKKYEGPRTAEALAEFVN<br>NEGGSNVKIAAVTSSVVVLTADNFNDIVLDENKDVLVEFYAPWCGHCKNLAPIYEKVATAFKSEE<br>DVVVANLEADKYRDLAEKYGVSGFPTLKFFPKGNKAGEEYEGGRDLDDFVAFINEKAGTSRDGK<br>GQLTSKAGIVESLDALVKEFVAAGDDEKKAVFSRIEEEVEKLKGSTARHGKIYLKAAKTCMVKGA<br>GYAKNEIERLQRMLEKSISPAKADEFTLKKNILSTFA
PtPDIL4-2	POPTR_014G122800v3	MEKYHQIWFAFGTLALLAVSALADDVVVLTEDNFEKEVGQDRGALVEFYAPWCGHCKKLAPEYE<br>KLGSSFRKAKTVLIGKVDCDEHKGVCSKYGVSGYPTLQWFPKGSLEPKKYEGPRTAEALTEYVNT<br>EGGTNVKIAAVPSNVAVLTADNFNNIVLDETKDVLVEFYAPWCGHCKNLAPTYEKVATAFKSEED<br>VVVANLDADKHKDLAEKYGVSGFPTLKFFPKGNKAGEDYEGGRDLDDFVAFINEKSGSSRDGKG<br>QLTSKAGIVESLDALVKEFVAAGDDEKKAVFSQIEEEVEKLKGSAARYGKIYSKAAKNCMAKGDY<br>AKNEIERLQRMLQKTISPAKADEFTLKKNILSTFA
PtPDIL5-1	POPTR_014G160000v3	MRTQSELLLLALSILFFQSNLFCYALYGPSSPVLQLNPSNFKSKVLNSNGVVLVEFFAPWCGHCKALT<br>PTWEKAAAVLKGVATVAALDADAHQSLAQEYGIRGFPTIKVFVPGNPPVDYQGARDVKPIAEYAL<br>KQIKALLKDRLNGKSTGGSSEKSETSLSVELNSRNFDELVLKSKELWIVEFFAPWCGHCKKLAPEW<br>TKAANNLQGKVKLGHVDCDSEKSLMSRFNVQGFPTILVFGADKDTPIPYEGARTASAIESFALEQL<br>ESNVAPPEVTELTGPDVMEEKCGSAAICFVAFLPDILDSKAEGRNKYLEQLLSVAEKFKRSPYSYV<br>WAAAGKQPDLENRVGVGGYGYPALVALNAKKGAYAPLKSAFELEHIVEFVKEAGRGGKGNLPLN<br>GNPEIVKTEPWDGKDGEIIEEDEFSLEELMGEDAGSKDEL
PtPDIL6-1	POPTR_009G004500v3	MINLNRSVSIWIVLSLFLSLFLSKAEVITLTPETFSDKVKEKDTAWFVKFCVPWCKHCKNLGTLWEE<br>VGKAMEGEDEIEVGEVDCGASKSVCSKADIHSYPTFKLFFDGEEVAKYQGPRDVESLKAFVLDEA<br>EKAAAKAQLGYDKDL
PtPDIL7-1	POPTR_019G082400v3	MKTRRSPLILLNTTPLLLLVLLSSSIISSAESTTPPEKINTVLELDESNFDSTISTYDYVFVDFYAPWCG<br>HCKRLAPELDVAAPILAELKKPIVIAKVNADKYTRLARKHEVDGFPTLKIYMHGVPTDYYGPRKAE<br>LLVCFLRKFVAPDVTILNSDSAIREFVEEAGTHFPIFIGFGLNETVMSNLAIKYKKKAWFSVASDFSD<br>DVMVQYDFDKIPALVSIHPSYNDHTVFYGPFEEEFMEEFITQNSLPLAVPINSETLKVLKDDQRKIVL<br>TILEDDSEEKSQNLIKILKAAASANRDLVFGFVGVKQWEEFTETFGANKETKLPKMIVWDGDEEYL<br>SVIGSESIEEEDQGSQISQFLAGYRGGRTERNRVSGPSLLGYISSLIGIRTVYIIVFLVAMLMFIQHISK<br>EEPLRVGTRDQAEPATSSKAESSEYRPEDKQD

PtPDIL7-2	POPTR_013G111400v3	MKTTRSSLILILSIIISATESTRADKINTVLELDESNFDSTIAAYDYVFVDFYAPWCTHCKRLAPELDV<br>AAPILAELKKPIVIAKVNADKYTRLARKHEVDGYPTLKIYMHGVPTEYYGPRKAELLVRFLRKFVA<br>PDVVVLNSDSAIREFVEEAGTHFPIFIGFGLNETLISNLAIKYKKKAWFSVASDFSDDVMVQYDFDK<br>IPTVVSIHPSYDDHSIFYGPFEEEFLEEFIEQNFLPLAVPINYDTLKVLKDDQRKIVLTILEDESEEKSQ<br>KLIKTLKAAASANRNLVFGYVGVKQWAEFAETFGAKGTKLPKMIVWDGGEEYLSVIGSESIEEED<br>QGSQISQFLAGYREGKTERNRISGPSLMGYLNSLIGVRTVYIIVFLVAMLILIRHISKEEPLTVGTGDQ<br>VEHATSSEAESSDYRPGDKQD
PtPDIL8-1	POPTR_001G419300v3	MVSTNKLKSVDFYRKIPRDLTEASLSGAGLSIVAALAMVFLFGMELNNYLTVNTSTSVIVDNSSDG<br>EFLRIDFNLSFPSLSCEFASVDVSDVLGTNRLNITKTIRKFSIDHDLKPTGSEFHSGPVLHHINHGDEV<br>HEEGSEGSVSLKAHNFDQYTHQYPILVVNFYAPWCYWSNRLKPSWEKAAKIIRERYDPEIDGRILL<br>AKVDCTEEGDLCRRNHIQGYPSIRIFRKGSDLRDDHGHHDHESYYGDRDTDSLVKTMEGLVAPIA<br>MESQRHALEHKPENATEHVKRPAPSAGGCRIEGYVRVKKVPGNLVISARSGAHSFDSAQMNLSHV<br>ISHFSFGMKVLPRVMSDVKRLIPHIGRSHDKLNGRSFINHRDVGANVTIEHYLQVVKTEVVTRRSSA<br>EHKLIEEYEYTAHSSLAQTVYMPTAKFHFELSPMQVLITENPKSFSHFITNVCAIIGGVFTVAGILDSI<br>LHNTFRMMKKVELGKNF
PtPDIL8-2	POPTR_011G135500v3	MVSTNKLKSVDFYRKIPRDLTEASLSGAGLSIVAALAMMFLFGMELNNYLTVNTSTTVIVDNSSDG<br>EFLRIDFNISFPSLSCEFASVDVSDVLGTNRLNITKTIRKFSIDHDLKPTGSEFHSGPVLHQIKHGDEV<br>DEEGGEGSVSLKAHNFDQYSHQYPILVVNFFAPWCYWSNRLKPSWEKAAKIIRERYDPEMDGRILL<br>AKVDCTEEGDLCRRNHIQGYPSIRIFRKGSNLREDHGRHDHESYYGDRDTESLVKTMEALVAPIAM<br>ESQRQALEHKPENATQHVKRPAPSAGGCRIEGYVRVKKVPGNLMISALSGAHSFDSKQMNLSHVIS<br>HFSFGMKVLPRVMSDVKRLLPYIGRSHDKLNGRSFINHRDVGANVTIEHYLQVVKTEVVTRRSSSE<br>RKLIEEYEYTAHSSLSQTVYMPTAKFHFELSPMQVLITENSKSFSHFITNVCAIIGGVFTVAGILDSIL<br>HHTVRMMKKVELGKNF
ZmPDIL1-1	Zm00001d049099	MAIRSKAWISLLLALAVALSARAEEEPAAAAEGEAVLTLDVDSFDEAVAKHPFMVVEFYAPWCGH<br>CKKLAPEYENAAKALSKHDPPIVLAKVDANEEKNRPLATKYEIQGFPTIKIFRDQGKNIQEYKGPRE<br>ADGIVDYLKKQVGPASKEIKSPEDATALIDDKKIYIVGIFAEFSGTEFTNFMEVAEKLRSDYDFGHT<br>LHANHLPRGDAAVERPLVRLLKPFDELVVDSKDFDVAALMKFIDASTIPRVVTFDKNPDNHPYLM<br>KFFQSSAPKAMLFLNFSTGPFDSFKSAYSAAAEEFKDKEIKFLIGDIEASQGAFQYFGLKEDQTPLILI<br>QDGDSKKFLKVHVEADQIVAWLKEYFDGKLTPFRKSEPIPEVNNEPVKVVVADNVHDFVFKSGKN<br>VLIEFYAPWCGHCKKLAPILDEAATTLQSDEEVVIAKMDATANDVPSEFDVQGYPTLYFVTPSGKV<br>TSYDSGRTADDIVDFIKKSKETAGAATTTTTQAPPASEKAAAAEPVKDEL
ZmPDIL1-2	Zm00001d007301	MAIRSKAWISLLLALAAVLSARAEEPAAAEAEAVLTLDVDSFDEAVAKHPFMVVEFYAPWCGHC<br>KNLAPEYENAAKELSKHDPPIVLAKVDANEEKNRPLATKYEIQGFPTLKIFRNQGKNIQEYKGPRE<br>ADGIVDYLKKQVGPASKEIKSAEGVAAHFDDKKIYIVGIFKEFSGTEFTNFMELAEKLSSDYDFGHT<br>LHANHLPRGDASVEGPLIRLLKPFDDLVVDSKDFDVAALEKFIDASSTPRVVTFDNNPDNHPYLMK

		FFQSSAPKAMLFLNFSTGPLDSFKSVYYAAAEEFKDKEIKFLIGDIEASQGAFQYFGLKEDQTPLILI QDGDSKKFLKDHIEADQIVSWLKEYFDGKLTPFKKSEPIPEVNNEPVKVVVADNIHDVVFKSGKNV LIEFYAPWCGHCKKLAPILEEAATTLLSDEEVVIAKMDATANDVPSEFEVQGYPTMYFVTPSGKVT SYDSGRTADDIVDFINKSKETASAVQATATASGKAADAAEKTEPVKDEL
ZmPDIL2-1	Zm00001d054106	MGSTTMSPPSFPVVLLLLLLATIAAAAGSNMDEEVVDDLQYLIDNSDDIPTNDPDGWPEGDYDDD DLLFQDQDQDLTGHQPEIDETHVVVLAAANFSSFLASSHHVMVEFYAPWCGHCQELAPDYAAAA AHLAAHHHQAHLALAKVDATEETDLAQKYDVQGFPTILFFIDGVPRGYNGARTKEAIVDWINKKL GPAVQNVTSVDEAQSILTGDDKAVLAFLDTLSGAHSDELAAASRLEDSINFYQTSTPDVAKLFHID AAAKRPSVVLLKKEEEKLTFYDGEFKASAIAGFVSANKLPLVTTLTQETSPSIFGNPIKKQILLFAVA SESTKFLPIFKEAAKPFKGKLLFVFVERDSEEVGEPVADYFGITGQETTVLAYTGNEDARKFFLDGE VSLEAIKDFAEGFLEDKLTPFYKSEPVPESNDGDVKIVVGKNLDLIVFDETKDVLLEIYAPWCGHCQ SLEPTYNNLAKHLRSVDSLVVAKMDGTTNEHPRAKSDGYPTILFYPAGKKSFEPITFEGERTVVDL YKFIKKHASIPFKLKRQESRTESTRAEGVKSSGTNSKDEL
ZmPDIL2-2	ZEAMMB73_444288	MGSTRTSHPSFPVLLLFLLATAAAAGSNKAEEVDDLQYLIDNSEDIPPNDPDGWPEGGGGGDYDD DLLFQDQDQDLPDYEPQIDETHVVVLTAANFSSFLAATRHVMVEFYAPWCGHCRELAPEYAAAA AHLAVHHNQTDLALAKADATEETDLAQRYDVQGFPTIILFIDGVPKDYNGARTKDAIVDWINKKL GPAVQDVTSVHEAERILTGDDKAVIAFLDTLTGAHSDELAAASRLEDSINFYQTSIPDVAKLFHIDP AAKRPSIVLLKKEEEKLTFYDGKFKASAIADFVSANKLPLVTTLTQETSPSIFGNAIKKQILLFAVAS ESSKFLSIFKEAAKPFKGKLLFVFVERDNDEVGEPVANYFGLTGQETTVLAYTGNEDARKFFLDGE VSLEAIKDFAEGFLEDKLTPFYKSEPVPESNDGDVKIVVGKSLDVIVLDESKDVLLEIYAPWCGHCQ SLEPTYNKLAKHLSGVDSLVIAKMDGTTNEHPRAKSDGYPTILFYPAGKKSFEPVTFEGERTVVDM YRFIKKHASIPFKLKRQESRRESIQTDGVKDEL
ZmPDIL3-1	Zm00001d045218	MRARWAVTLLLLAVLALTASAARLDLDDDDDSGVLDELLAIDEEAERGGLLDAEGAGEAVRRAQ SMVLALDNDNARRAVEDHAELLLLGYAPWCERSAQLMPRFAEAAAALRAMGSAVAFAKLDGER YPKAAAAVGVKGFPTVLLFVNGTEHAYHGLHTKDAIVTWVRKKTGVPIIRLQSKDSAEEFLKKDM TFVIGLFKNFEGADHEEFVKAATTDNEVQFVETSDTSVAKVLFPGITSEEKFVGLVKSEPEKFEKFD GKFEEKEILRFVELNKFPLITVFTELNSGKVYSSPIELQVFTFAEAYDFEDLESMVEEIARAFKTKIMF IYVDTAEENLAKPFLTLYGLESEKKPTVTAFDTSNGAKYLMEADINANNLREFCLSLLDGTLPPYH KSEPLPQEKGLIEKVVGRTFDSSVLESHQNVFLEVHTPWCVDCEAISKNVEKLAKHFSGSDNLKFA RIDASVNEHPKLKVNNYPTLFLYLAEDKSNPIKLSKKSSVKDMAKLIKEKLQIPDVETVAAPDNVK DEL
ZmPDIL4-1	Zm00001d040766	MAISQISRIFLAILLLAAAFAAAPAALADGDDVVALTESTFEKEVGKDRGALVEFYAPWCGHCKKL APEYERLGASFKKAKSVLIAKVDCDEHKSLCSKYGVSGYPTIQWFPKGSLEPKKYEGQRTAEALAE FLNTEGGTNVKLATIPSSVVVLTPETFDSIVLDETKDVLVEFYAPWCGHCKSLAPTYEKVASVFKLD EGVVIANLDADKHRDLAEKYGVSGFPTLKFFPKGNKAGEDYDGDRDLVDFVKFINEKSGTSRDTK

		GQLTSEAGRIASLDVLAKEFLGASGDKRKEVLSSMEEEADKLSGSAARHGKVYVTIAKKILEKGNE<br>YTEKETKRLDRILEKVGNAYLARCLMKHPLLGQLTVQI
ZmPDIL4-2	Zm00001d037590	MAFPQISRRALGLLLVIAAAAAIVSPATADEVVALTEADFEKEVGQDRGALVEFYAPWCGHCKKL<br>APEYEKLGASFKKAKSVLIAKVDCDEHKSVCSKYGVSGYPTIQWFPKGSLEPKKYEGQRSVEALAE<br>FVNSEAGTNVKIAAIPSSVVVLTSETFDSIVLDETKDVLVEFYAPWCGHCKHLAPIYEKLASVFKQD<br>DGVVIANIDADKHTDLAEKYGVSGFPTLKFFPKGNKAGEDYDGGRDLDDFVKFINEKCGTSRDPK<br>GHLNQEAGLVPSLNPLVKEFLNAADDKRKEVLSKIEEDVAKLSGSAAKHGKIYVTAAKKIIDKGSD<br>YTKKETERLHRMLEKSISPSKADEFIVKKNILSIFSS
ZmPDIL5-1	Zm00001d020687	MRPAVVTVLLLVAAAASPAAALYSAGSPVLQLNPNNFKSKVLNSNGVVLVEFFAPWCGHCKQLA<br>PAWEKAAGVLKGVATVAALDADAHQALAQEYGIKGFPTIKVFSPGKPPVDYQGARDVKPIVEFAL<br>SQVKSLLRDRLSGKASAGSNGKTSGGSSEKSEPSASVELNSRNFDELVVKSKDLWIVEFFAPWCGH<br>CKKLAPEWKKAAKNLKGQVKLGHVDCDAEKSLMSKYKVEGFPTILVFGADKESPFPYQGARVAS<br>AIESFALEQLEANSGPAEVSELTGPDVMEEKCASAAICFVSFLPDILDSKAEGRNKYLELLLSVAEKF<br>KKSPYSFVWTAAGKQANLENQVGVGGYGYPAMVALNVKKGAYAPLRSAFQRDEIIEFVKEAGRG<br>GKGNLPLNDAPTVVASEPWDGKDGEVIEEDEFSLDELMGDSSSANDEL
ZmPDIL6-1	Zm00001d028726	MDLGAPARRRLPIRLLLVSLTVLVVLTARSSAEVITLTEETFSDKIKEKDTVWFVQFCVPWCKHCK<br>NLGTLWEDLGKVMEGADEIEIGQVDCGVSKPVCSKVDIHSYPTFKVFYEGEEVVKYKASMDYTRT<br>NCSVVGTLRTRTSPAATRSRTKCRSISTCFVR
ZmPDIL7-1	Zm00001d025131	MAARVLPPPPLPLVLLLLLLPLSARDTVAAGEDFPRDGRVIDLDESNFEAALGVIDFLFVDFYAPWC<br>GHCKRLAPELDEAAPMLAGLSEPIVVAKVNADKYRKLGSKYGVDGFPTLMLFIHGVPIEYTGSRK<br>ADQLVRNLKKFVAPDVSILESDSAIKNFVENAGTSFPMFLGFGVNDSLIAEYGRKYKKRAWFAVA<br>KDFSEDVMVAYEFDKVPALVAIHPKYKEQSLFYGPFEENFLEDFVRQSLLPLVVPINTETIKMLNDD<br>QRKVVLTILEDDSDENSTQLVKILRSAASANRDLVFGYVGIKQWDEFVETFDVSKSSQLPKLLVWD<br>RNEEYELVDGSERLEEGTDQASQISQFLEGYRAGRTTKKKISGPSFMGFLNSLVSLTSLYILIFVIALL<br>FVMVYFAGQDDTPQPRRIHEE
ZmPDIL7-2	Zm00001d016786	MAMALRRLLLPLLLLVLLGLRPQSCVASGGGGGEPAEFEIPRDGSVLELDESNFEAAVRAAEFLFV<br>DFYAPWCGHCKRLAPQLDEAAAVLAGLSTPVLVAKVNADKYKKLGSKYGVDGFPTLMFFDHGV<br>PSEYTGSRKADVLVENLKKLVAPDVSVLESDSSINGFVQAAGINFPLFIGFGMDESLIVEYGAKYKK<br>KAWFSTAKDFSEDVMVVYDFDKVPALVSVNPKYNEQSVFYGPFEGTFLEDFIRQSLLPATVPINRE<br>TVKLLKDDGRKVVLTILEDESDESSLQLIKVLRSAANANHDLVFGYVGVKQWEEFTETFDVKVSQ<br>LPKIVVWDTKEEYEVVEGSESFIEGDYGSQVSRFLEGYREGRTTKKKVGRGSPTLLGLNAVYILVL<br>LVAVLVVLMYFSAQGEEDHQPRRAHED
ZmPDIL8-1	Zm00001d006512	MISSSKLKSVDFYRKIPRDLTEASLSGAGLSIVAALAMVFLFGMELSSYLAVNTTTSVIVDRSSDGEF<br>LRIDFNMSFPALSCEFASVDVSDVLGTNRLNITKTVRKYSIDRNLVPTGSEFHPGPIPILNKHGDDVE<br>EDHVDGAFSLSSRNFDSFSHQYPVLVVNFYAPWCYWSNRLKPSWEKTAKIMRERYDPEMDGRILL

		GKVDCTEEVELCRRNHIQGYPSIRVFRKGSDIKENQGHHDHESYYGERDTESLVAAMETYVANIPK<br>EAHALEDKSNKTVDPAKRPAPMASGCRIEGFVRVKRVPGSVVISARSGSHSFDPSQINVSHYVTQFS<br>FGKRLSPRMLHEFIRLTPYLRGYHDRLAGQSYTVKHGEVNANVTIEHYLQVVKTELVTQRSSKELK<br>VLEEYEYTAHSSLVHSFYVPVVKFHFEPSPMQVLVTEVPKSFSHFITNVCAIIGGVFTVAGILDSIFH<br>NTLRMVKKIELGKNI
BdPDIL1-1	Bradi4g23180	MAICNKAWISLLLALAVVLAAPAARAEEAAAAEEAAPAAGEEAVLTLGTDNFDDAIAKHPFIVVEF<br>YAPWCGHCKSLAPEYEKAAQLLSKHDPPIVLAKVDANDEKNKPLAAKYEIQGFPTLKIFRNQGKNI<br>QEYKGPREAEGIVDYLKKQVGPASKEIKAPEDASHLEDGKIHIVGVFAELSGPEFTNFLEVAEKLRS<br>DYDFGHTVHANHLPRGETAVERPLVRLFKPFDELVVDTKNFEVSALEAFIEASSTPKVVTFDKNPD<br>NHPYLLKFFQGNSAKVMLFLNFSTGPYESFKSAYYGAVEDFKDKEVKYLIGDIEASQGALQYFGLN<br>ADQAPLILIQDAESKKFLNSNIEADQIVSWLKEYFDGKLTPFRKSEPIPEANNEPVKVVVADNLDDV<br>VFKSGKNVLIEFYAPWCGHCKKLAPILDEAATTLQSEADVVIAKMDATANDVPGDFDVQGYPTLY<br>FVTPSGKKVAYDGGRTADDIVEYIKKNKETAGQEAAAATEKAADPAATESLKDEL
BdPDIL1-2	Bradi5g10610	MAVPLALPSAAIIIVVLLLSSGLTTAEVEVAAVLEEAVLTLDVSNFSEVVGKLQFIVVEFYAPWCGH<br>CKELAPEYEKAASMLRKHDPPVVLAKVDAYDEGNKELKDKYEVHGYPAIKIIRNGGSDVSGYAG<br>ARNADGIVEYLKKQVGPASIELRSALDATRSIGDKGVVLVGIFPEFAGVEYENFMAVADKMRSDY<br>DFFHTSDASILPHGDQNVKGPLVRLFKPFDELFVDSQDFDKDAIKKFIEVSGFPTVVTFDDEPTNHK<br>FLERYYSTPSAKAMLFLRFSDDRVEAFKSQMHEAARQLSGNNISFLIGDVSAAERAFQYFGLKESDI<br>PLLLVIASTGKYLNPTMDPDQLIPWMKQYIYGNLTPYVKSEPIPKVNDQPVKVVVADNIDDIVFNS<br>GKNVLLEFYAPWCGHCRKLAPILEEVAVSFRNDEDIVIAKMDGTANDVPTDFVVEGYPALYFYSSS<br>GGEILSYKGARTAEEIISFIKKNRGPKAGALEEVTQTDAVQEEVTSTSSPSESVKDEL
BdPDIL2-1	Bradi3g00210	MVSPRSLLLLLLLASPFLLLLLHASSDEDLDYIIHNAAADDLPADDEWLQEGSDDDQEESDPFHQG<br>DIDETHVFLLTAANFSDFLSSRRHVMVEFYAPWCGHCQALAPDYAAAASQLALLHQDVVALSKV<br>DATEDADLAQKYDVQGFPTILFFIDGVPKDYTGERTKEAIVAWINKKLGPGVHNVTTVDEAEKIIT<br>GEDKAVLAFLDSLSGAHSNELAAASRLEDTINFYQTSNPDVAKLFHIDPAAKRPSVVLLKKEEEKL<br>TIYEGEFRASAIADFVSANKLPLITILTQETGPSIFDNPIKKQILLFAVANESSEFLPIFKEVAKPFKGKL<br>LFVFVERDNEEVGEPVANYFGITGQETTVLAYTGNEDAKKFFLDGEMSLDNIKKFAQDFLEDKLTP<br>FYKSEPIPEPNDEDVKIIVGKNLDQIVLDESKDVLLEIYAPWCGHCQSLEPTYNKLAKHLRGIDSLVI<br>AKMDGTTNEHPRAKPDGFPTILFYPAGKKSFEPMTFEGDRTVVEMYKFIKKHASIPFKLKRPDSSA<br>ARTERAESSGSTEGEKSSGSNLKDEL
BdPDIL3-1	Bradi1g48460	MRAWRRLAVVALLAALLAVSSAAARLDLGEDDDSEVLEALLAVDEEEEDEAPEGAKRAGGAEAV<br>RRTQSMVLVLDNENARRAVEEHAELLLLGYAPWCERSAQLMPRFAEAAAALRAMGSAVAFAKL<br>DGERYPKAAADVGVSGFPTVLLFVNGTEHAYTGLHTKDALVTWVRKKTGAPVIRLQSRDSAEEFL<br>KKDQTFAIGLFKNYEGADHEEFVKAATTENEVQFVETNDRNVAKILFPGIASEEQFLGLVKSEPEKF<br>EKFDGAFEENAILQFVELNKFPLITVFTDLNSGKVYGSPIKLQVFTFAEAYDFEDLESLLQEVARGFK

		TKIMFIYVDTAEEKLAKPFLTLYGLEGDKPTVTAFDTSKGSKYLMEADINAKNLKEFCSGLLDGTL<br>PPYFRSEPVPQEKGLIGKVVGRTFDSSVLESPHNVFLEAHAPWCVDCEAISKNVEKLAKHFSGLDN<br>LKFARIDASVNEHPKLQVNDYPTLLLYPAEDKSNPIKVSKKLSLKDMAKFIKVKLHISDVDIKEKEP<br>ASDVEAVAATDSVKDEL
BdPDIL4-1	Bradi2g12560	MAIRQISRQTLALLFVVAAASFAALVFADGDDVVVLTEGTFEKEVGQDRGALVEFYAPWCGHCK<br>KLAPEYEKLGASFKKARSVMIAKVDCDEHKSVCSKYGVSGYPTIQWFPKGSLEPKKYEGQRTAEA<br>LAEFVNKEGGTNVKLATIPSSVVVLTPETFDSVVLDETKDVLVEFYAPWCGHCKHLAPIYEKLASA<br>FKLDDGVVIANVDADKYKDLGEKYGVTGFPTLKFFPKGNKAGEDYDGGRDLGDFTKFINEKCGTS<br>RDTNGQLTSEAGRIASLDTLAKEFLSVASDKRKEVLSSIEEEVAKLSGSAAKHGKVYVTIAKKILDK<br>GNDYTKKETERLHRILEKSISPSKADEFIIKKNVLSTFSS
BdPDIL4-2	Bradi2g35020	MATPQISRKTLALVLLLVAAAAAALSPAAAEGDEVLALTESTFDKEVGQDRAALVEFYAPWCGHC<br>KKLAPEYEKLAASFKKAKSVLIAKVDCDEHKSVCSKYGVSGYPTIQWFPKGSLEPKKYEGQRTAE<br>ALAEYVNSEAATNVKIAAVPSSVVVLTEETFDSVVLDETKDVLVEFYAPWCGHCKSLAPVYEKVA<br>SAFKLEDGVVIANLDADKHTSLAEKYGVSGFPTLKFFPKGNKAGEEYEGGRDLEDFVKFINEKSGT<br>SRDSKGQLTSEAGLVASLDALVKEFHSAADDKRKEVLSKIEEEAAKLSGSAAKHGKIYVNAAKKII<br>EKGSDYTKKETERLHRMLEKSISPSKADEFVIKKNILAIFSS
BdPDIL5-1	Bradi4g31830	MHPAFLAALLLLFAAAASPAAALYSAGSPVLQLNPNNFKKVLNANGVVLVEFFAPWCGHCKQLTP<br>TWEKAAGVLKGVATIAALDADAHKELAQQYGIQGFPTIKVFIPGKPPVDYEGARDVKPIVNFALQQ<br>VKSLLKDRLDGKTSGGSSGKTSGGSSEKKTDTNESIELNSSNFDELVIKSKDLWIVEFFAPWCGHCK<br>KLAPEWKRAAKNLKGQVKLGHVDCDSDKSLMSKYKVEGFPTILVFGADKESPFPYQGARAASAIE<br>SFALEQLEANSAPPEVSELTSSDVMEEKCASAAICFVSFLPDILDSKAEGRNKYLELLLSVAEKFKKS<br>PYSFVWTGAGKQADLEKQVGVGGYGYPAMVALNVKKGAYAPLRSAFQRDEIIEFVKEAGRGGKG<br>NLPLDGAPTVVQSGPWDGKDGEVIEEDEFSLEELMGDNSPPNDEL
BdPDIL6-1	Bradi1g65710	MDPALRRRSRLPIHMLVLVVAVLVVLAARSGAEVITLTEETFTDKVKEKDTVWFVQFCVPWCKHC<br>KSLGTLWEDLGKVIEGTDEIEIGKVDCGASKPVCSKVDIHSYPTFKVFYDGEEVAKYKGPRNVESL<br>KNFVLNEAEKAGEARLQDEL
BdPDIL7-1	Bradi5g10380	MATTLPPLPLLCLLPLLLVAFATAGGGSGGGGGENFPRDGRVIDLDDSNFEAALSSIDFLFVDFYAP<br>WCGHCKRLAPELDEAAPVLAGLSEPIMVAKVNADKYRKLGSKYGVDGFPTLMLFIHGVPIEYTGS<br>RKADLLVRNLKKFVAPDVSILESDSAIKSFVENAGTSFPMFIGFGVNESLITEYGGKYKKRAWFAIA<br>QDFSEELMMAYGFDKAPALVALHPKYNEQSVFYGPFEGRFLEDFIRQSLLPLTVPINTETLKLLDDD<br>DRKVVLAILEDDSDENSAQLVTVLRSAANANRDLVFGYVGVKQWEEFVETFDVSKSSQLPKLLV<br>WDRNEEYEQVDGSERLEEGDQASQISQFLEGYRAGRTTKKKVSGPSFMGFMHSLVSMNSLYILMF<br>VVALLGVMLYFSGQDDTPQLRRIHDE
BdPDIL7-2	Bradi3g45540	MAAGKPLSLRRLLPLLALVLVLVLLPTTCVSSGGGEPAGFQIPQDGSVVELDDSNFEAAAAAVDFL<br>FVDFHAPWCGHCKRLSPQLDEAAPVLAGLSTPVVVAKVDAEKYKKLGSKYGVDGFPTLMLFDHG

		VPTEYTGSRKADLLIQSLKKLVAPDFSVLGSDSAIKSFVQDAGVGFPLFIGFGVDESSIVEYGTRYKR<br>KAWFAAAKDFSEDMMVVYDFDKIPALVSLNPKYNEQSVFYGPFEGTFLEDFIRQSLLPLTVPINAE<br>TVKMLKDDERKVVLTVLEDESDENSMQLIKVLRSAANANHDLVFGYVGVKQWEEFTEPFHDSESS<br>RLPRMVVWDRNEEYEVVQGSENLEDGDHGSQISRFLEGYRAGRTTKKKLGGRSPTILGVNAMYIL<br>LFLVAVLVVLMYFSGQGEEDRWPARAHQE
BdPDIL8-1	Bradi1g25977	MISSSKLKSVDFYRKIPRDLTEASLSGAGLSIVAALAMVFLFGMELSSYLAVNTTTSVIVDRSSDGEF<br>LRIDFNMSFPALSCEFASVDVSDVLGTNRLNITKTVRKFSIDRNLVPTGSEFHSGPIPTVNKHGDDVE<br>EYHADGSVALSSRNFDSYSHQYPILVVNFYAPWCYWSNRLKPSWEKTAKIIKERYDPEMDGRILLA<br>KVDCTEEGELCKRHHIQGYPSIRIFRKGSDMKENQGHHDHESYYGERDTDSLVAAMETYVGNLPK<br>EAHMLALDDKSNKTVDPAKRPAPMTSGCRVEGFVRVKKVPGSVIISARSGHSFDPSQINVSHYVT<br>QFSFGNRLSPNMFSELKRLIPYVGGHHDRLAGQSYIVKHGDNNANVTIEHYLQIVKTELVTLRSSKE<br>LKVFEEYEYTAHSSLVHSFYVPVVKFHFEPSPMQVLVTELPKSFSHFITNVCAIIGGVFTVAGILDSIL<br>HNTLRLVKKVELGKDI

Table S4. Primers used for qRT-PCR assay to analyse the expression of the *SlPDI* genes

Target Genes	Primer sequence (5' to 3')
SLPDI1-1	AAGCCAGAATGATCCACCAG
	AACAATACCATCCGCCTCAC
SLPDI1-2	GCCCTGCATCTCTTGAAATC
	GGAAGGAGTTTGGCATCAAG
SLPDI1-3	GGTAATTGTTTCAGCTCTTG
	GTCCACACCAAGGTGCATAG
SLPDI1-4	AACCTGATGTTCTTGCTTCATG
	TTCACTGGCTCGTTGTTAACC
SLPDI2-1	GGCAACAAGAGCTTTGAACC
	TGCTCTCGTGGCTTAAACTG
SLPDI2-2	GTTCTAGCATATCTCGACTCC
	TAGCTTAGCCACGTTAGGATC
SLPDI2-3	GTCCTACTCACTCCATCACT
	ACGGAACTTCAAAATCGTCG
SLPDI3-1	TACCACGCCAACAAGCATAG
	TGTTTTCGGGACTGTTCAGG
SLPDI4-1	CTTCAAAGGCGGGTATTGTC
	CCCTTCAGTTTTCCAGCTTC
SLPDI5-1	TCACATACGAAGGTGCAAGG
	CAAAGCAGATTGCAGCAGAG
SLPDI6-1	CAATGGAAAGGGAGGATGAG
	CTTGGTACTTGGCGACTTCTTC
SLPDI7-1	CAAGGATACAAAGACGGAAA
	TCGTCCCTTGTACCAACAGT
SLPDI7-2	GGATACAGAGATGGAAGTGT
	GCTCTTCTTTAACAGCCTGC
SLPDI8-1	TTCAGGCTCAACTGCAACAG
	TCTCCCATGAAGGTTTCAGC
SLPDI9-1	TATCTCAGCCCAGGCAAAAC
	CACCTGCTTGCAACTCTTTG
SLPDI10-1	ATGCGGTATCATGGCTCTCG
	CAGGAGAATGGACAGTTTTC
SLPDI11-1	TGGCTTTGGCTTTCAGTTCT
	CCTCTGAGTAAAATTCACTGC
SLPDI11-2	TGAACAACCCAAAGCCATTG
	CATTCCTCTTAGGTTCAGCA
SLPDI11-3	ATGGCTTTGGCTTTCACTTC
	CAGCACAACGCCTCTGAGTA
Le18S (internal control gene)	CGGCTACCACATCCAAGGAAGG
	GAGCTGGAATTACCGCGGCTG
